# Individual variation in the emergence of anterior-to-posterior neural fates from human pluripotent stem cells

**DOI:** 10.1016/j.stemcr.2024.07.004

**Published:** 2024-08-15

**Authors:** Suel-Kee Kim, Seungmae Seo, Genevieve Stein-O’Brien, Amritha Jaishankar, Kazuya Ogawa, Nicola Micali, Victor Luria, Amir Karger, Yanhong Wang, Hyojin Kim, Thomas M. Hyde, Joel E. Kleinman, Ty Voss, Elana J. Fertig, Joo-Heon Shin, Roland Bürli, Alan J. Cross, Nicholas J. Brandon, Daniel R. Weinberger, Joshua G. Chenoweth, Daniel J. Hoeppner, Nenad Sestan, Carlo Colantuoni, Ronald D. McKay

**Affiliations:** 1 Lieber Institute for Brain Development, 855 North Wolfe Street, Baltimore, MD 21205, USA; 2 Department of Neuroscience, Yale School of Medicine, New Haven, CT 06510, USA; 3 Departments of Genetics, Psychiatry, and Comparative Medicine, Kavli Institute for Neuroscience, Program in Cellular Neuroscience, Neurodegeneration and Repair, Child Study Center, Yale School of Medicine, New Haven, CT 06510, USA; 4 Department of Systems Biology, Harvard Medical School, Boston, MA 02115, USA; 5 Division of Genetics and Genomics, Boston Children’s Hospital, Harvard Medical School, Boston, MA 02115, USA; 6 IT-Research Computing, Harvard Medical School, Boston, MA 02115, USA; 7 Departments of Cell Biology, Johns Hopkins School of Medicine, Baltimore, MD 21205, USA; 8 Departments of Neurology, Johns Hopkins School of Medicine, Baltimore, MD 21205, USA; 9 Departments of Oncology, Biomedical Engineering, and Applied Mathematics and Statistics, Johns Hopkins School of Medicine, Baltimore, MD 21205, USA; 10 Departments of Psychiatry, Johns Hopkins School of Medicine, Baltimore, MD 21205, USA; 11 Departments of Neuroscience, Johns Hopkins School of Medicine, Baltimore, MD 21205, USA; 12 McKusick-Nathans Institute of Genetic Medicine, Johns Hopkins School of Medicine, Baltimore, MD 21205, USA; 13 Division of Preclinical Innovation, Nation Center for Advancing Translational Science, NIH, Bethesda, MD 20892, USA; 14 Astra-Zeneca Neuroscience iMED., 141 Portland Street, Cambridge, MA 01239, USA; 15 Institute for Genome Sciences, University of Maryland School of Medicine, Baltimore, MD 21201, USA

**Keywords:** pluripotent stem cells, cell line variation, anterior or posterior neural fates, SOX21, retinoic, acid signaling, early embryo, human population

## Abstract

Variability between human pluripotent stem cell (hPSC) lines remains a challenge and opportunity in biomedicine. In this study, hPSC lines from multiple donors were differentiated toward neuroectoderm and mesendoderm lineages. We revealed dynamic transcriptomic patterns that delineate the emergence of these lineages, which were conserved across lines, along with individual line-specific transcriptional signatures that were invariant throughout differentiation. These transcriptomic signatures predicted an antagonism between SOX21-driven forebrain fates and retinoic acid-induced hindbrain fates. Replicate lines and paired adult tissue demonstrated the stability of these line-specific transcriptomic traits. We show that this transcriptomic variation in lineage bias had both genetic and epigenetic origins, aligned with the anterior-to-posterior structure of early mammalian development, and was present across a large collection of hPSC lines. These findings contribute to developing systematic analyses of PSCs to define the origin and consequences of variation in the early events orchestrating individual human development.

## Highlights

• Cell imaging and transcriptomics reveal stable lineage bias across hPSC lines
• Antagonism between SOX21 and RA signaling regulates regional neural lineage bias
• Lineage biases are present in large collections of public hPSC lines
• Lineage bias landscape across hPSC lines corresponds to mammalian development

## Graphical abstract

**Figure:**
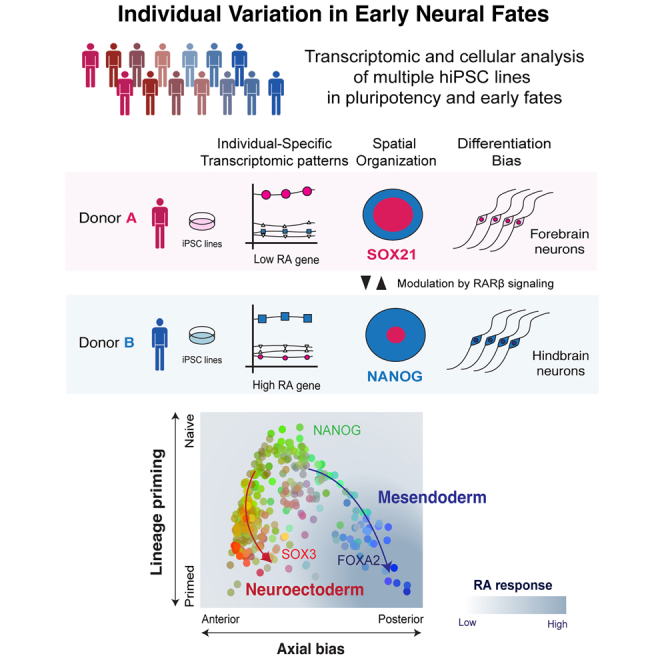

## Teaser

Variation in the lineage bias of pluripotent cells from different humans can be mapped onto the primary axes of *in vivo* mammalian development. This variation in cell potential is driven by stable, individual-specific transcriptomic signatures. These gene expression traits likely influence health, disease, and therapeutic responses throughout our lifetime.

## Introduction

During mammalian embryonic development, pluripotent epiblast cells undergo spatially constrained cell state transitions to form distinct tissues (Arnold and Robertson, 2009). Human pluripotent stem cells (hPSCs) represent the epiblast state, primed to diversify into the embryonic germ layers, ultimately forming major organs (Bao et al., 2009; Brons et al., 2007; Tesar et al., 2007). Previous studies have examined genomic and transcriptomic variations in hPSC lines (Carcamo-Orive et al., 2017; Choi et al., 2015; Cuomo et al., 2020; DeBoever et al., 2017; Kilpinen et al., 2017; Rouhani et al., 2014). However, we lack a detailed understanding of variation in the transitions from pluripotent cells to neural stem cells with distinct brain regional identities, complicating stem cell applications for neurological and psychiatric disorders. Methods to direct hPSCs to various tissues *in vitro* are advancing, focusing on reproducible cellular output from diverse lines. It is now of great interest to develop assays that further explore the origins of inherent variation in cellular phenotypes among hPSC lines.

Recent studies report developmental differences among hPSC lines in generating regionally specified neural precursors and their possible implications for neurodevelopmental disorders (Jourdon et al., 2023; Kanton et al., 2019; Mariani et al., 2015; Micali et al., 2020; Paulsen et al., 2022; Wang et al., 2020). Here, we employ cellular and genomic approaches to define functional variation in hPSC lines as they progress through neuroectoderm versus mesendoderm and fore- versus hindbrain development. High-resolution decomposition of gene expression during hPSC differentiation revealed dynamic transcriptomic changes in lineage emergence that were conserved across lines. In addition, these tools defined gene expression specific to individual hPSC lines and donors that remained stable throughout prolonged cell culture and repeated differentiation. These line-specific signatures are regulated by both genetic and epigenetic mechanisms that act through SOX21 and retinoic acid (RA) signaling to control fore- and hindbrain trajectories. Our extensive multi-omics data, combined with relevant public datasets, are accessible at https://nemoanalytics.org/p?l=Kim2024.

## Results

### Cell line variation in the emergence of neural fate from pluripotency

Previous studies have defined diverse lineage-competent states generating distinct embryonic and extraembryonic fates on unconstrained (Guo et al., 2021; Hough et al., 2014), surface-patterned (Warmflash et al., 2014), and microfluidic-directed (Rifes et al., 2020) hPSC colony organizations. Here, we measured spatial dynamics of early cell fate emergence in unconstrained monolayer culture by monitoring the expression of key lineage regulators and signaling targets. One day after the passage of dissociated single cells (day 0, D0), rho-kinase (ROCK) inhibitor was removed to allow undifferentiated cells to form epithelial sheets. To induce differentiation, hPSCs were exposed to bone morphogenetic protein (BMP)/transforming growth factor β (TGF-β) signaling agonists or antagonists to generate mesendodermal or neural fates, respectively (Chambers et al., 2009; Faial et al., 2015). BMP4 treatment on D0 (D0T) induced phosphorylation of SMAD1/5 and expression of early primitive streak and extraembryonic fate regulators TBXT and CDX2 across the cell population (Figure S1A). When BMP4 was applied on D2 (D2T), the induction of these markers was restricted to the edge of the epithelium (Figures S1A and S1B), while cells in the core remained competent to respond to BMP4, as indicated by SMAD2/3 phosphorylation and expression of SOX17 and GATA4, drivers of primitive and definitive endoderm differentiation (Figure S1B). BMP/TGF-β signaling antagonists Noggin and SB431542 (NSB) induced neural differentiation, with SOX2, SOX21, and OTX2 expressed in the epithelium core. In contrast, edge cells maintained a high level of NANOG (Figures S1C and S1D). These findings indicate that this unconstrained two-dimensional system defines spatial domains in early neuroepithelial lineage emergence.

**Figure 1:**
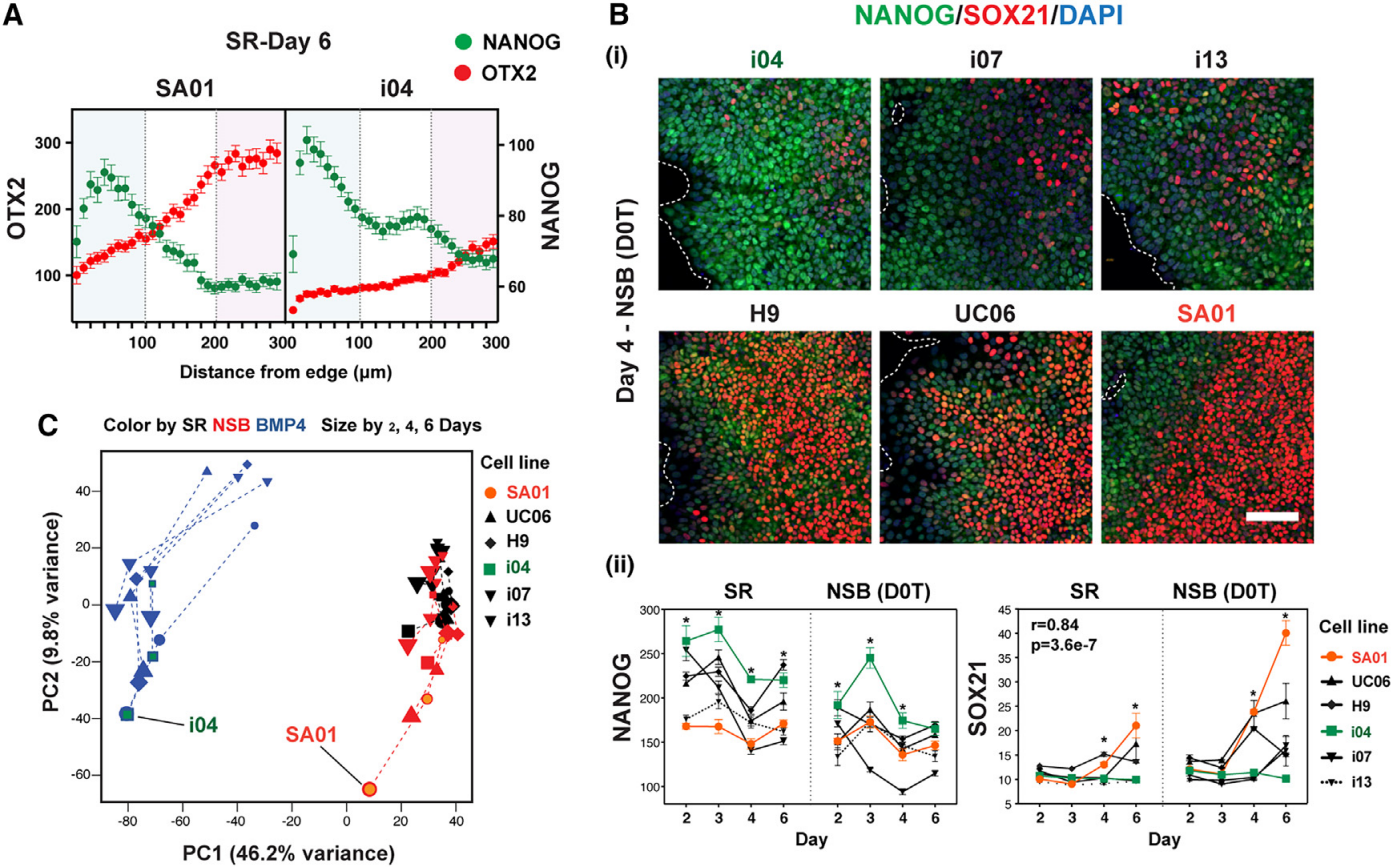
Cell line variation in the emergence of neural fate from pluripotency (A) Spatial expression on day 6 in SR condition in SA01 and i04 lines. (B) Variation in NANOG and SOX21 expression across PSC lines. (i) Representative images. Scale bar, 100 μm. (ii) Expression levels in each line across time (*n* = 5, independent experiments; ^∗^, Comparison between SA01 and i04: *p* < 0.001; r and *p* value refer to the Pearson correlation coefficient between SOX21 levels in SR and NSB). (C) PCA showing differentiation trajectories. See also Figure S1.

To measure variation in this spatial organization across multiple hPSC lines, we compared hESC line SA01 with hiPSC line i04 (Mallon et al., 2013). SA01 displayed larger core zones with higher SOX21 and OTX2 expression, while i04 showed larger edge zones with greater NANOG expression (Figures 1A and S1E). These differences persisted under various doses of neuroectodermal inducers and cell-plating densities (Figures S1F and S1G) and were not due to different proliferation rates. In response to BMP4, i04 rapidly induced CDX2, while SA01 predominantly expressed TBXT (Figure S1G). These data suggest that cell line variation in early fate bias can be defined in this system.

Variation in early neural fate was indicated by differential SOX21 expression among six hPSC lines in both self-renewal (SR) and neuroectoderm (NSB) conditions (Figure 1B; Table S1). SA01 line showed the lowest NANOG level and the highest SOX21 induction under neuroectoderm conditions, while line i04 showed the opposite, suggesting an inverse correlation between NANOG and SOX21 expression during neuroectoderm emergence. Principal-component analysis (PCA) of RNA sequencing (RNA-seq) data at 2, 4, and 6 days of SR, NSB, or BMP4 conditions for all lines (Table S1) showed that principal component (PC) 1 corresponded to mesendodermal differentiation and PC2 to temporal changes in differentiation in all conditions (Figure 1C). All cell lines followed similar trajectories along PC1 and PC2, while differences were also evident. SA01 advanced furthest along the NSB trajectory, aligning with SOX21 levels (Figures 1C and S1H). This bias in differentiation was evident in the PCA of NSB samples alone (Figure S1I). Projecting SR data into the NSB dataset revealed that the same ranking of cell lines was present in SR, indicating that the gene expression differences in differentiation were already present in pluripotency (Figure S1I). Projection of SR data into BMP4 PC1 also showed a consistent inverse ranking. These observations suggest that SOX21 expression marks early neuroectodermal specification and that heterogeneity within pluripotency is linked to the emergence of early cell fate bias between lines.

### Decomposing dynamic and stable transcriptomic modules in early differentiation

**Figure 2:**
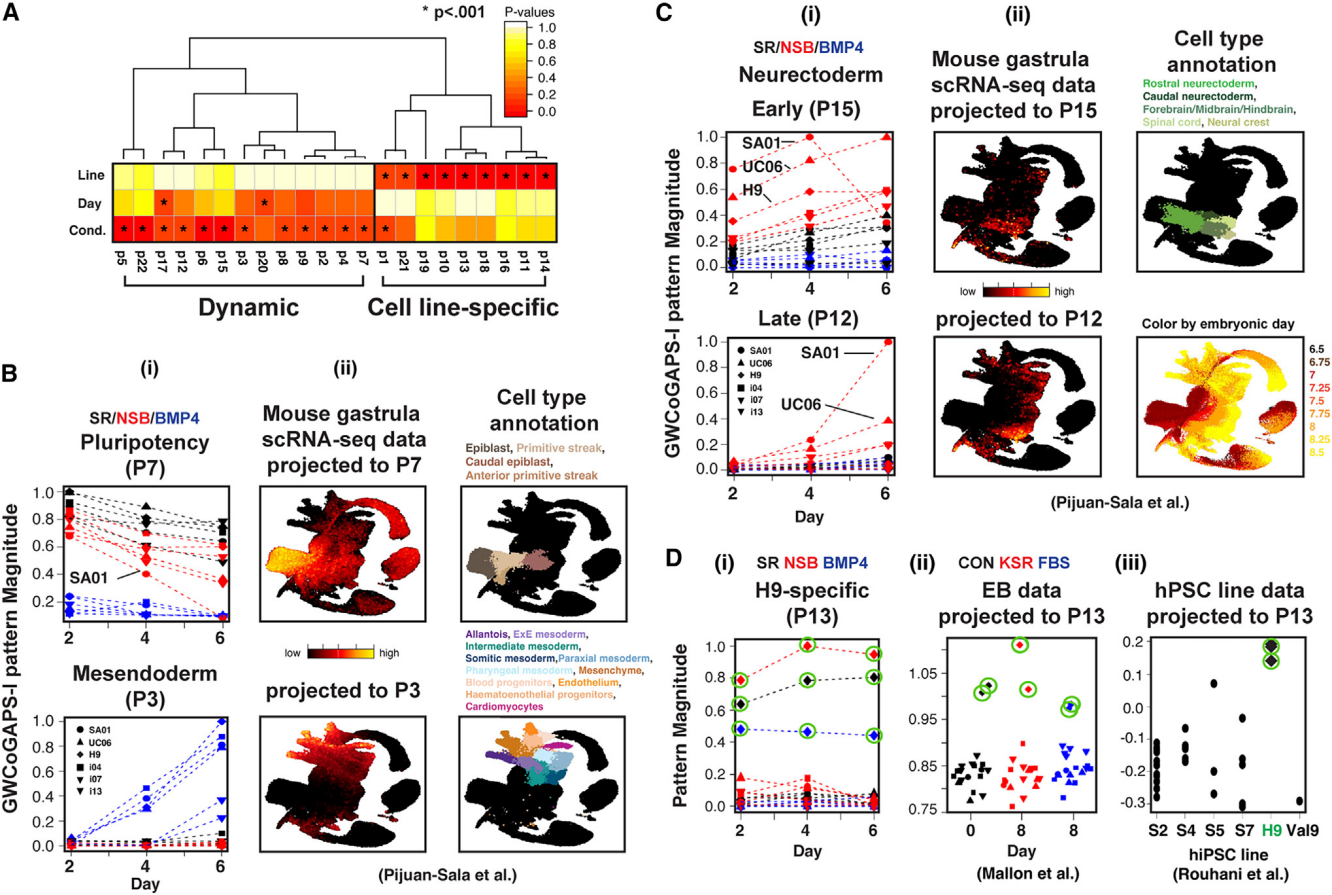
Decomposing dynamic and cell line-specific transcriptomic modules (A) Hierarchical clustering of GWCoGAPS-I patterns and *p* values from ANOVA analysis of effects of line, day, and condition in each pattern. (B) (i) GWCoGAPS-I patterns representing loss of pluripotency (P7) or mesendoderm induction (P3). (ii) Projections of mouse gastrula data. (C) (i) NSB patterns delineating early and later stages of neuroectoderm differentiation. (ii) Projection of mouse gastrula data. (D) (i) H9-specific transcriptomic signature. (ii) Projection of embryoid body (EB) data from the same lines (Comparison between H9 and other lines, *p* = 3.0e−4). (iii) Projection of multiple hPSC line data. H9 samples are circled in green. See also Figure S2.

To further dissect this low-dimensional transcriptomic change across cell lines and conditions, we employed the Genome-Wide Coordinated Gene Activity in Pattern Sets (GWCoGAPS) non-negative matrix factorization algorithm (Stein-O'Brien et al., 2017) and identified 22 transcriptomic patterns (GWCoGAPS-I, Table S1). GWCoGAPS patterns decompose multiple signals within individual gene expression. Hence, a combination of patterns represents the complete expression of each gene (Figure S2A). This analysis revealed two classes of patterns (Figure 2A): dynamic patterns, which characterized transcriptomic trajectories that changed over time or conditions, and cell line-specific patterns, which defined consistent differences over time and conditions but varied between cell lines.

Among the dynamic patterns (Figure 2A), three patterns, including P7, related to pluripotency; 6 patterns, including P3 and P9, represented a response to BMP4; and three patterns, including P15 and P12, captured different temporal phases of response to NSB. P7 included the core pluripotency genes *POU5F1*, *SOX2*, and *NANOG*. BMP4-induced patterns P3 and P9 included the early mesendodermal and extraembryonic fate regulators *TBXT*, *EOMES*, and GATA family members. NSB patterns P12 and P15 contained the neuroectodermal regulators *OTX2*, *SOX21*, and *PAX6* (Figures S2B; Table S1).

To relate these *in vitro* transcriptomic dynamics with *in vivo* development, we projected single-cell RNA-seq (scRNA-seq) data from developing mouse gastrula (Pijuan-Sala et al., 2019) into the GWCoGAPS-I patterns. The pluripotency module P7 showed the highest levels in epiblast cells and decreased in early germ layer populations (Figures 2B and S2C). In contrast, BMP4 pattern P3 genes increased in mesodermal lineages and posterior primitive streak derivatives. Patterns P15 and P12 genes peaked in neuroectodermal cells (Figures 2C and S2D). Sequential induction of these early neural expression modules was also found in cortical neuron differentiation from multiple hiPSC lines (Burke et al., 2020) (Figure S2Diii).

Genes with high weights in cell line-specific patterns showed higher expression in specific cell lines (Figures 2A, 2Di, and S2E). In general, genes with high weights in dynamic patterns exhibited low weights in cell line-specific patterns (Figure S2E). However, some dynamic pattern genes, such as *OTX2*, *SOX21*, *and ZIC3*, had higher weights in cell line-specific patterns of lines showing more efficient generation of neuroectoderm, while *GBX2* and *FABP7* had higher weights in those of lines more efficient at generating mesendoderm. This suggests that cell line-specific and dynamic transcriptomic signatures interact to influence the early differentiation of cell lines.

Projection of our previous microarray data from the differentiation of the same lines (Mallon et al., 2013) showed that line-specific patterns were stable across vastly different laboratory conditions (Figures 2Dii and S2Fii). Projection of RNA-seq data from other studies using multiple lines, including H9 (Choi et al., 2015; Kyttala et al., 2016; Rouhani et al., 2014), into the H9-specific pattern showed this line with the strongest signal (Figures 2Diii and S2G). Additionally, projection of DNA methylation data indicated that cell line-specific patterns correlate with hypomethylation at promoters of genes highly expressed in the corresponding cell lines (Figure S2Fiii). These findings suggest that cell line-specific patterns define stable transcriptomic and epigenetic signatures in individual hPSC lines.

### SOX21 regulates early forebrain fate by inhibiting mesendoderm and neuromesoderm specification

**Figure 3:**
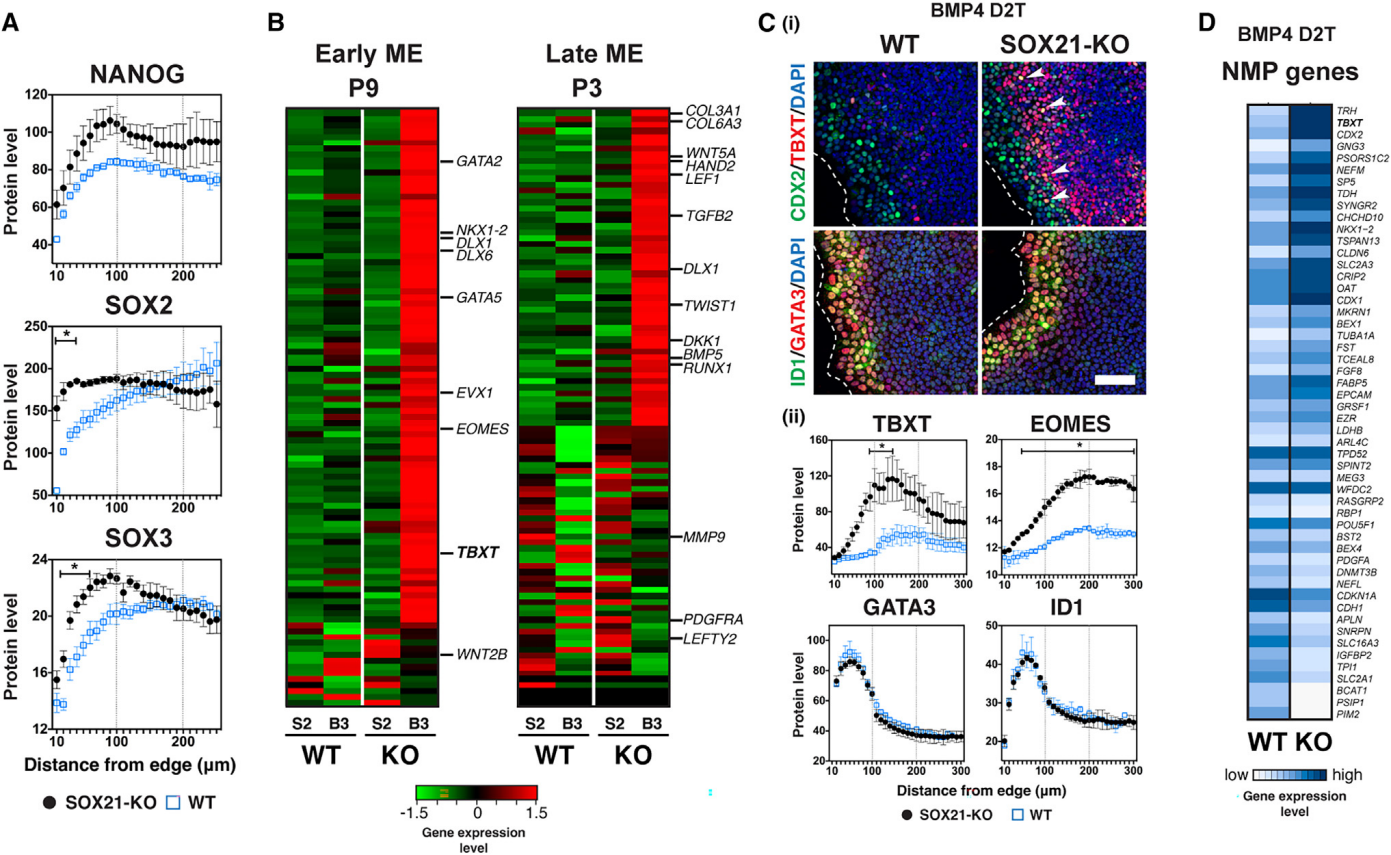
SOX21 mediates early forebrain fate (A) Spatial expression on D3 in NSB condition. (B) Expression of the top 100 genes in P9 and P3 on D2 SR (S2) and 24 h after BMP4 treatment on D2 (B3). (C) (i) Expression of mesendodermal regulators 24 h after BMP4 treatment on D2. Arrowheads indicate coexpression of TBXT and CDX2. Scale bar, 100 μm. (ii) Spatial expression. ^∗^, Comparison between WT (*n* = 3, replicate cell lines) and SOX21-KO (*n* = 3): *p* < 0.05. (D) Expression of NMP genes 24 h after BMP4 D2T. See also Figure S3.

While the neuroectoderm pattern P15 represents the early transcriptomic changes following NSB treatment, it also exhibited differential responses across lines (Figures 2A and 2C). The high rank of *SOX21* in P15 (Figure S2B) and its induction with OTX2 in NSB condition (Figure S1D) suggest a role in the variation of the cell lines in early forebrain specification. We previously suggested that SOX21 interacts with SOX2 to regulate antero-posterior identity in the adult mouse intestine by repressing CDX2 (Kuzmichev et al., 2012). To define SOX21’s role in early neural specification, we generated SOX21-knockout (KO) lines from the SA01 line (Figure S3A). After NSB treatment, SOX21-KO lines showed increased SOX2 and SOX3 expression in the epithelial edge and increased NANOG in both edge and core zones (Figure 3A). Transcriptomic changes in SOX21-KO lines (Figures S3B and S3C) further supported that the loss of SOX21 resulted in sustained pluripotency and delayed transition to neural fates.

To test SOX21’s relative role in the emergence of mesendodermal and neural fates, SOX21-KO cells were treated with BMP4 on D2T after the core zone had formed. While wild-type (WT) cells showed minimal induction of mesendodermal genes, SOX21-KO cells exhibited strong upregulation of BMP4-responsive genes (Figure 3B). Immunostaining showed no difference in GATA3 and ID1 expression between WT and SOX21-KO lines in the edge zone. However, TBXT and EOMES were significantly induced in the core zone of SOX21-KO cells (Figure 3C). Notably, CDX2 expression extended in the core zone of SOX21-KO cells, resulting in increased TBXT and CDX2 coexpression. In the posterior region of embryos, neuromesodermal precursors (NMPs) generate both spinal cord and trunk mesoderm (Henrique et al., 2015). Consistent with CDX2’s role in specifying NMP (Guibentif et al., 2021), the NMP transcriptomic signature and SOX21 expression were mutually exclusive in the mouse gastrula (Figure S3D). Many NMP genes showed higher expression in SOX21-KO cells when treated with BMP4 on D2 (Figure 3D). These results indicate that SOX21 restricts mesendoderm and NMP during the early stages of anterior-to-posterior (A-P) neural fate determination.

SOX21’s role in the early specification of rostral and caudal neuroectoderm was supported by the observation that genes upregulated in SOX21-KO cells under NSB condition showed high expression in the epiblast and anterior primitive streak of developing mice (Figure S3Dvi). Interestingly, the restriction of caudal identity was evident from genes upregulated in the SOX21-KO cells under BMP4 D2T condition and in the forebrain of the gastrulating mouse embryo. *TBXT*, which specifies pro-mesoderm and neural crest in the caudal epiblast (Gogolou et al., 2022), was among the targets of SOX21 repression (Figures 3B–3D and S3Dv). Previous studies have reported SOX21’s role in promoting extraembryonic fates (Goolam et al., 2016), late forebrain development (Fang et al., 2019), and adult neurogenesis (Matsuda et al., 2012). Our findings support an early inhibitory role for SOX21 during the transition from pluripotency, restricting posterior neuroectodermal fates and promoting anterior fates in the emerging forebrain.

### Cell line-specific transcriptomic signatures underlie variation in forebrain versus hindbrain fate bias

**Figure 4:**
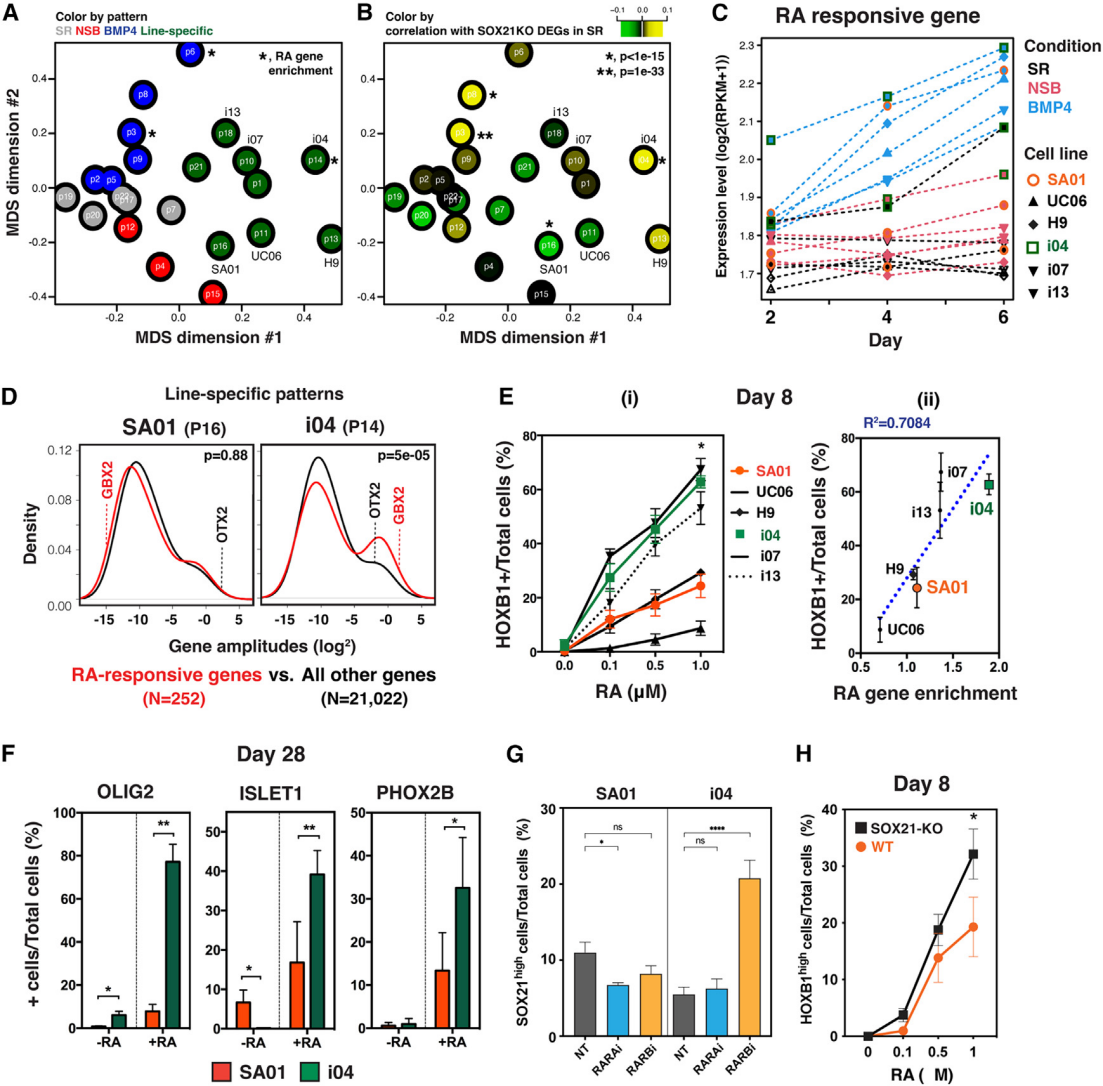
Cell line-specific transcriptomic signatures underlie variation in forebrain versus hindbrain fate bias (A) MDS plot of gene amplitudes showing correlation between GWCoGAPS-I patterns. ^∗^, patterns with RA-responsive gene enrichment (P3, *p* = 9.7e−08; P6, *p* = 0.014; P14, *p* = 0.008). (B) MDS plot colored by correlation of each pattern’s gene weights with DEGs in SOX21-KO cells in SR condition. (C) Average expression of RA genes. (D) Distribution of gene weights of RA genes in cell line-specific patterns. (E) (i) Proportion of HOXB1^hi^ cells after RA treatment in NSB condition. ^∗^, comparison between SA01 and i04 (*p* < 0.05). (ii) Correlation of HOXB1^hi^ cell proportions and RA gene enrichment in each cell line-specific pattern. *n* = 3, independent experiments. (F) Different production of hindbrain neurons in response to RA Comparison between SA01 and i04 (∗, *p* < 0.05; ^∗∗^, *p* < 0.01). *n* = 3, independent experiments. (G) Proportion of SOX21^hi^ cells after RAR inhibitor treatment on D4 in NSB condition (^∗^, *p* < 0.05; ^∗∗∗∗^, *p* < 0.0001). *n* = 6, technical replicates. (H) Proportion of HOXB1^hi^ cells in SOX21-KO lines after RA treatment in NSB condition. Comparison between WT and SOX21-KO (^∗^, *p* < 0.05). *n* = 3, replicate cell lines. See also Figure S4.

We used multidimensional scaling (MDS) to relate cell line-specific signatures to lineage emergence (Figure 4A). The first MDS dimension distinguished dynamic patterns from cell line-specific signatures. The second dimension separated the NSB patterns from the BMP4 patterns, positioning the SR patterns between the lineage-related patterns. Gene expression changes by SOX21-KO in pluripotency correlated with BMP4 patterns P3 and P8 (Figure 4B), aligning with SOX21’s role in repressing mesendoderm specification. In addition, these changes positively correlated with the i04 line-specific signature and negatively with the SA01 line-specific signature. These opposing correlations suggest that these line-specific signatures interact with SOX21, influencing the lineage bias of these lines.

To further examine the differential A-P axis patterning in these lines, we interrogated the expression of genes known to be upregulated by RA signaling (Balmer and Blomhoff, 2002), which plays a role in posterior mesendodermal, neural, and neuromesodermal development (Ghyselinck and Duester, 2019). Consistently, RA-responsive genes were highly expressed in caudal epiblast and posterior neural fates of mouse gastrula (Figure S4A). These genes were significantly enriched in two BMP4 patterns (Figure 4A) and in SOX21-KO cells in BMP4 condition (Figures 4B and S4B). RA exposure reduced SOX21 expression in a dose-dependent manner (Figure S4C). Furthermore, i04 line exhibited the highest expression of RA-responsive genes across all conditions (Figure 4C). Notably, the i04 line-specific signature was significantly enriched with RA-responsive genes (Figure 4D) and aligned with BMP4 patterns in MDS dimension 2 (Figure 4A). In contrast, the SA01 line’s signature showed no RA-responsive gene enrichment and aligned with NSB patterns. These results suggest the lower SOX21 expression in i04 line might be related to its high RA signaling activity.

Opposing interactions between OTX2 and GBX2 establish the mid-hindbrain boundary, with the RA-responsive GBX2 expressed in the hindbrain (Millet et al., 1999). These were differentially expressed between SA01 and i04 lines (Figures S4D and S4E), with GBX2 showing higher gene weights in the i04-specific pattern (Figure 4D). Since cell line-specific patterns define stable transcriptomic features, the differential enrichment of RA-responsive genes in these patterns could predict the A-P differentiation efficiency of individual cell lines. To test this hypothesis, we assessed RA dose response in neural differentiation across cell lines and observed that i04, i07, and i13 produced more hindbrain cells than SA01, H9, and UC06 (Figures 4Ei and S4F). RA gene enrichment in the cell line-specific patterns strongly correlated with hindbrain fate potential (Figure 4Eii). Further differentiation showed higher production of hindbrain neurons in i04 (Figure 4F). Thus, cell line-specific signatures associated with RA signaling can predict the differential emergence of anterior versus posterior neuronal fates in hPSC lines.

Next, we modulated RA signaling by applying RA receptor (RAR) inhibitors to the cells. Both *RARA* and *RARB* genes were upregulated during mesendodermal differentiation, with *RARB* gene expression highest in SR condition, particularly in the i04 line (Figure S4G). Inhibiting RARβ in neuroectoderm condition increased SOX21 expression in i04 cells (Figure 4G), suggesting that high RARβ-driven signaling in this line contributes to inefficiency in generating anterior neuroectoderm. We further examined RA response in SA01 line-derived SOX21-KO lines and observed a higher RA response (Figure 4H), indicating that SOX21 can suppress RA-responsive gene expression. These results demonstrate an interplay between SOX21 expression and endogenous RA signaling activity, underlying cell line variation in their response to RA and subsequent A-P identity.

### Cell line-specific signatures involve evolutionarily recent genes under genetic control

**Figure 5:**
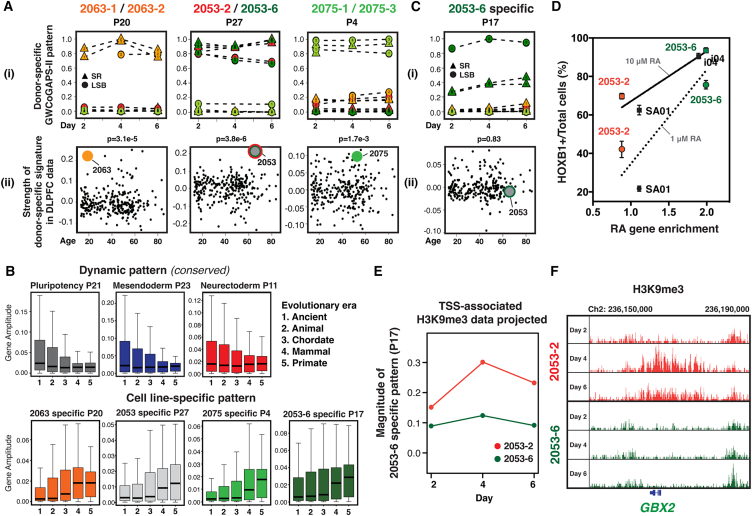
Genetic and epigenetic elements contribute to donor- and line-specific transcriptomic signatures (A) (i) Donor-specific patterns. (ii) Projection of 260 human brain data. Significance confirmed by permutation: 2,053, *p* = 3.8e−6; 2075, *p* = 1.7e−3; 2063, *p* = 3.1e−5. (B) Contribution of genes of different evolutionary eras to GWCoGAPS-II patterns. Ancient genes (era 1) show high gene amplitudes in conserved dynamic patterns (compared to era 5, Wilcoxon rank-sum test: *p* < 1e−16 for all 3 dynamic patterns). Primate-specific genes (era 5) show higher gene amplitudes in cell line-specific patterns (*p* < 1e−16 for all 4 line-specific patterns). (C) 2053-6 line-specific pattern and projection of brain data. (D) Correlation of HOXB1^hi^ cell proportions and RA gene enrichment in cell line-specific patterns (R^2^ = 0.75 in 1 μM RA and R^2^ = 0.87 in 10 μM RA). The proportion in line 2053-2 was correlated with 2053 donor-specific pattern. *n* = 3, independent experiments. (E) Projection of ChIP-seq data from lines 2053-2 and 2053-6 in SR into the 2053-6 line-specific pattern. (F) H3K9me3 ChIP-seq data at the *GBX2* locus. See also Figure S5.

We next generated six new hiPSC lines, including two replicate lines each from three adult donors whose postmortem brain RNA-seq data were previously generated (Jaffe et al., 2018)(Table S1). The dynamic differentiation trajectories observed in the original hPSC lines (GWCoGAPS-I) were recapitulated in the new analysis (GWCoGAPS-II, Table S2) also identifying transcriptomic signatures that were stable across time and conditions for each donor (Figure 5Ai). Projection of RNA-seq data from the cerebral cortex of 260 individuals into these donor-specific patterns showed that the expression traits were elevated in the corresponding postmortem tissue (Figure 5Aii). Moreover, the donor-specific signatures were also highest in the parental fibroblasts of the same donors (Figure S5A). These expression profiles across replicate lines, fibroblasts, and mature brain tissue suggest that donor-specific signatures comprise stable gene expression traits potentially persisting across an individual’s cells throughout their lifespan.

The Genotype-Tissue Expression (GTEx) Project analyzed gene expression and genetic variation across multiple tissues from numerous donors, identifying multi-tissue expression quantitative trait loci (eQTLs) (GTEx Consortium, 2015). Genes with high amplitudes in our donor-specific signatures were significantly enriched in these eQTLs (p = 1e−6 to p = 5e−21), suggesting their stable expression. To assess the genetic origins of these stable signatures, we compared the strength of donor-specific signatures in brain RNA-seq data with genetic similarity among donors, using single-nucleotide polymorphism genotypes in these brains (Jaffe et al., 2018)(Figure S5B). We observed significant correlations (*p* = 4.5e−7 and *p* = 2.5e−8) between genetic similarity and the strength of the projected donor-specific signatures, indicating that genetic factors influence donor-specific transcriptomic signatures.

Next, we analyzed the distribution of evolutionary gene ages across the gene weights of GWCoGAPS-II patterns. This revealed that dynamic patterns shared across all lines had stronger weights in ancient genes (Figure 5B). In contrast, dynamic patterns varying among lines exhibited a higher contribution of newer genes (Figure S5C). Importantly, evolutionarily recent genes showed the highest contribution in cell line-specific patterns (Figures 5B and S5D). These findings suggest that conserved dynamic changes in pluripotency and differentiation are ancient and part of the “Waddington landscape” (Ferrell, 2012) that constrains cellular differentiation paths. In contrast, stable transcriptomic patterns related to individual human variation are newer and influence how cells of individual hPSC lines follow particular paths within this cellular landscape.

Additionally, estimates of gene dosage sensitivity (Collins et al., 2022) indicate that genes in cell line-specific signatures are less crucial to survival, reflecting their recent evolutionary origin. We found that the top 1% of each GWCoGAPS-I pattern had lower gene dosage sensitivity in cell line-specific patterns (average probability of haploinsufficiency, pHaplo; 0.32–0.47) compared to other patterns (pHaplo; 0.53–0.72).

### Cell line-specific signatures can also be driven by early epigenetic mechanisms

In addition to donor-specific signatures (Figure 5A), GWCoGAPS-II revealed a 2053-6 line-specific signature, which was not present in the brain data of the same donor (Figure 5C). Projection of the 2053-6 line-specific signature showed no correlation with genetic distance between donors (Figure S5B), suggesting that cell line-specific patterns can dissect distinct genetic and epigenetic origins.

Projection of the new cell line RNA-seq data into the GWCoGAPS-I neuroectodermal patterns revealed that all new lines showed similar forebrain fate induction, except 2053-6 (Figure S5E). Line 2053-6 showed less SOX21 induction than 2053-2 (Figure S5F), similar to the differences between SA01 and i04. RA-responsive genes were enriched in the 2053-6 line-specific pattern, and 2053-6 line generated more hindbrain cells following RA treatment (Figure S5G). These data indicate that the decision to preferentially form fore- versus hindbrain fates between 2053-2 and -6 lines may be regulated by stable epigenetic differences.

Genome sequencing was performed on 2053-2 and -6 lines and brain tissues from donors 2053 and 2075 (Figure S5H). For both donors, most copy-number variations were shared in all donor tissues and cell lines, indicating minimal genetic changes during reprogramming. Therefore, the discordant expression traits and lineage bias between 2053-2 and -6 lines were not due to large-scale genome differences.

Notably, we found that Kruppel-associated box domain zinc finger (KRAB-ZNF) genes were significantly enriched in all line- and donor-specific signatures (Table S2) and expressed at distinct levels among lines (Figures S5I and S5J). KRAB-ZNF genes repress transposable elements and establish persistent H3K9me3-mediated heterochromatin to regulate gene expression during early development (Ecco et al., 2016). These observations suggest that early KRAB-ZNF-driven H3K9me3 heterochromatin mechanisms in pluripotency may shape persisting transcriptomic phenotypes that influence cell function.

To explore this further, we generated chromatin immunoprecipitation sequencing (ChIP-seq) data for histone modification from 2053-2 and 2053-6 lines in SR condition. Projection into the 2053-6 line-specific pattern showed enrichment of the H3K9me3 repressive mark in 2053-2 at promoters of genes overexpressed in 2053-6, while H3K4me3 and H3K27me3 showed no such difference (Figures 5E and S5K). This suggests that many genes in 2053-6 have been de-repressed via specific loss of H3K9me3, leading to stable expression phenotypes and posterior fate bias. In particular, *GBX2* was highly represented in the 2053-6 line-specific signature and showed higher H3K9me3 levels in 2053-2 (Figures 5F and S5L; Table S3). This distinct heterochromatin setting between the lines in pluripotency underscores the importance of exploring how early epigenetic mechanisms contribute to divergent transcriptomic phenotypes and lineage fate bias.

### Early lineage bias and RA signaling define hPSC variation in the wider human population

**Figure 6:**
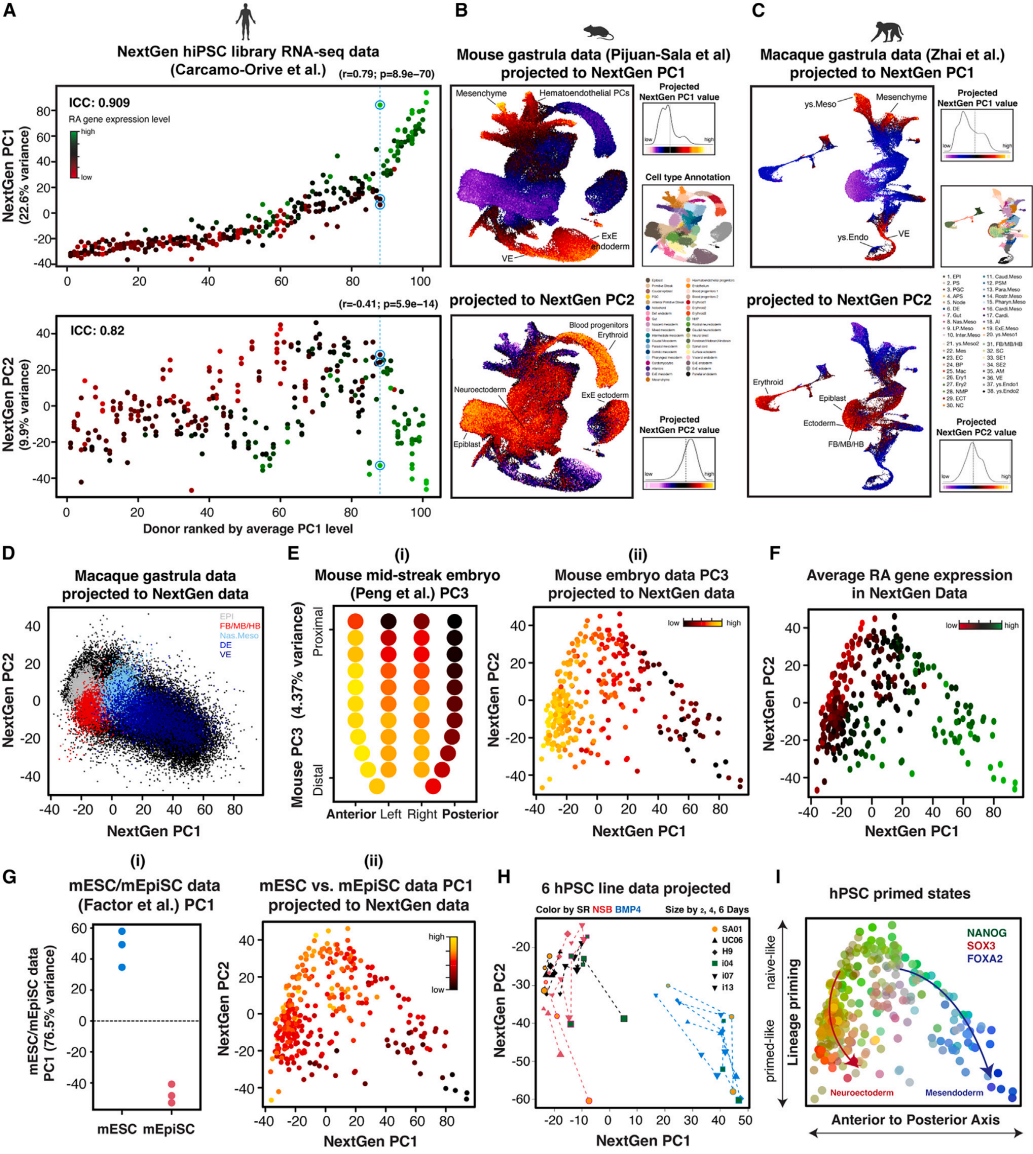
Early developmental bias and expression of RA-responsive genes define hPSC variation in the human population (A) PCs of the NextGen RNA-seq data. Donors are ordered along the X axis by the average PC1 level of all replicates. Pearson’s R and *p* values indicate correlations of PC1 and PC2 with mean RA gene expression. Blue circles highlight lines from one donor with high variance in PCs and RA gene expression. Intraclass correlation coefficient (ICC) estimates proportion of transcriptomic variation across lines attributed to the donor of origin. (B) NextGen PC projection into mouse gastrula data. (C and D) NextGen PC projections into macaque gastrula data. (E) (i) PC3 of the mouse (embryonic day 7) embryo data. (ii) Projection into the NextGen PCs. (F) Average RA gene expression in NextGen PCs. (G) (i) PC1 represents higher gene expression in mESCs. (ii) Projection into NextGen PCs. (H) Projection of 6 hPSC line data into NextGen PCs. (I) A model illustrating how biased gene expression in hPSCs drives (arrows) anterior/neuroectodermal or posterior/mesendodermal differentiation. See also Figure S6.

To explore the generality of these features in the human population, we performed PCA on RNA-seq data from 317 undifferentiated hiPSC lines derived from 101 donors generated by the NextGen Consortium (Carcamo-Orive et al., 2017). PC1 exhibited a strong positive correlation with RA-responsive gene expression, while PC2 showed a negative correlation, indicating that the differences in RA response observed in a small number of lines reflect broader systematic variation (Figure 6A). A significant proportion of variance in PC1 (91%) and PC2 (82%) was derived from inter-donor differences, suggesting that genetic factors play a dominant role in the transcriptomic variation across hPSC lines. In contrast, certain hiPSC lines from the same donor displayed significantly divergent positions along PC1 and PC2, as well as distinct RA-responsive gene expression levels, suggesting that epigenetic mechanisms also contribute to this transcriptomic variation.

Projection of the NextGen PCs onto early mouse development revealed a strong lineage bias (Figure 6B). High PC1 values corresponded to mesodermal and endodermal identities. RA-responsive genes with the highest PC1 loadings were highly expressed in self-renewing cells at the edge of pluripotent colonies (Hough et al., 2014) and in pluripotent founder cells with primitive endoderm identities (Nakanishi et al., 2019). High PC2 values were associated with epiblast, primitive streak, and hematopoietic lineage identities. These lineage associations were also observed when projecting the NextGen PCs onto macaque gastrula data (Zhai et al., 2022) (Figure 6C). Projecting macaque epiblast and early lineage data into NextGen PCs revealed a systematic distribution of lineage bias within hPSC variation (Figures 6D and S6A). Cell lines with high PC1 values displayed high expression of mesenchyme, erythroid, and endothelium-specific genes in human embryos, while cell lines with low PC1 and PC2 values exhibited high expression of neural progenitor genes (Figure S6B) (Zeng et al., 2023).

These early lineage features were further explored by projecting GWCoGAPS-I dynamic patterns (Figure S6C). Cell lines with low PC1 values exhibited higher levels of pluripotency patterns, while those with high PC1 values showed higher levels of mesendoderm patterns. Lines with low PC1 and PC2 values displayed higher levels of neuroectoderm patterns. The strong associations with A-P regional identities were validated by projecting mouse embryo data (Peng et al., 2016) (Figure 6E; Table S4). Consistently, the lines showing posterior bias exhibited higher expression levels of RA-responsive genes (Figure 6F; Table S4). These findings indicate that the transcriptomic heterogeneity in hPSC lines aligns with the developmental A-P axis and lineage emergence in mammalian embryos.

Interestingly, projecting mouse inner cell mass-derived embryonic stem cell (mESC) and epiblast-derived stem cell (mEpiSC) data (Factor et al., 2014) revealed that cell lines with high NextGen PC2 values displayed a higher mESC identity (Figure 6G; Table S4). This suggests that these PSC line variations exhibit gradients of naive and primed pluripotent transcriptomic signatures (Figures 6G and 6I).

Furthermore, we projected cell line-specific patterns into the NextGen data (Figure S6D). The transcriptomic signatures of SA01 and 2053-2 lines, which exhibited neuroectoderm and forebrain bias, were more pronounced in cell lines with lower NextGen PC1 values, aligning with their anterior bias. In contrast, the signatures of lines i04 and 2053-6, displaying mesendoderm and hindbrain bias, were elevated in lines with higher NextGen PC1 values, reflecting their posterior bias. This analysis further supports that cell line-specific signatures contain transcriptomic traits predicting lineage bias.

Projection of the initial six-line data, including early differentiation conditions (Figure 1C), into NextGen PCs confirmed the lineage structure within the pluripotency landscape (Figure 6H). This analysis clearly separated SR and neuroectoderm from mesendoderm differentiation along NextGen PC1. The progression from SR toward early lineages aligned with Nextgen PC2. These findings, summarized in Figure 6I, emphasize the broad utility of mapping lineage bias in hPSCs as these early cellular assays within pluripotency have the potential to predict functional differences across extensive hPSC line collections.

## Discussion

Studies are increasingly revealing the genetic origins of heterogeneity in transcription and differentiation potential of hPSCs (Bonder et al., 2021; Carcamo-Orive et al., 2017; Choi et al., 2015; Cuomo et al., 2020; DeBoever et al., 2017; Jerber et al., 2021; Kilpinen et al., 2017; Kyttala et al., 2016; Merkle et al., 2022; Ortmann et al., 2020; Puigdevall et al., 2023; Rouhani et al., 2014; Strano et al., 2020). Our analysis identifies dynamic lineage-driving transcriptomic modules and cell line-specific gene expression traits. We demonstrated that the relationship between cell line-specific and dynamic patterns enables the identification of key regulators predicting functional phenotypes of PSC lines during differentiation. The observed bias in neural and mesendodermal lineage in a small number of hPSC lines aligned with the transcriptomic variation across hundreds of hPSC lines prior to differentiation, suggesting this variation in lineage bias is a general feature of the human population. Low variance within donors and persistent donor-specific expression traits in adult tissues suggest that major aspects of this transcriptomic variation in pluripotency are long-lasting and genetically controlled. In addition, a difference in bias toward fore- versus hindbrain fates in replicate lines from the same donor indicates that epigenetic mechanisms also contribute to variation in early neural fate choice. These data support a model where, in addition to genetic variation, alternate epigenetically predisposed states exist within pluripotency prior to the implementation of anterior or posterior regional fate choice.

In contrast to the conserved dynamic expression patterns, cell line-specific patterns were enriched with recently evolved genes. KRAB-ZNF gene enrichment and H3K9me3-mediated regulation in these cell line-specific signatures suggest a potential epigenetic mechanism controlling their stable expression phenotypes. Recent studies have revealed subtle variations in H3K9me3 that function in cellular reprogramming and early lineage plasticity (Buckberry et al., 2023; Hoetker et al., 2023). Understanding how H3K9me3, associated with individual signatures, interacts with other cell type-specific histone modifications will provide deeper insight into the epigenetic regulation of human variation in lineage bias.

Recent scRNA-seq data from cerebral organoids (Kanton et al., 2019; Rosebrock et al., 2022) revealed striking differential forebrain fate bias across human donors. Our previous work (Micali et al., 2020) defined early variation in transcriptomic patterns resulting in a neural fate bias along the dorsoventral telencephalic axis. Variation in dorsoventral specification has been observed across many lines and linked to risk for neuropsychiatric diseases (Mariani et al., 2015; Paulsen et al., 2022; Strano et al., 2020). In this current study, we focused on differential RA signaling, which drives lineage bias along the A-P axis. Dysregulation of RA signaling has been associated with the risk for schizophrenia and autism (Shibata et al., 2021). Further dissecting the consequences of variation in early RA response and other morphogenetic regulators is important to better understand potential clinical implications.

Advances in selecting specific hPSC lines for stem cell therapeutics (Andrews et al., 2022; Merkle et al., 2022) highlight the importance of defining genetic and epigenetic controls influencing human cellular variation during differentiation. Our work contributes to understanding how variation in early cellular states influences human brain development to modify complex traits and disease risk. As PSC-derived models of early mammalian development become increasingly sophisticated (Pera, 2023), using synthetic embryos from diverse PSC lines holds great promise to systematically explore the consequences of this early inherent variation at later steps of human brain development and function.

## Experimental procedures

### Resource availability

#### Lead contact

Further information and requests should be directed to and will be fulfilled by the lead contact Ronald D. McKay (ronaldmckay@mac.com).

#### Materials availability

Cell lines generated in this study are available from the lead contact upon request.

#### Data and code availability

The accession number for the RNA-seq data reported in this paper is deposited in GEO Database: GSE164055, with linked raw read files in the National Library of Medicine Sequence Read Archive under BioProject PRJNA688712.

(https://www.ncbi,nlm.nih.gov/sra)

GWCoGAPS decompositions:

(https://www.bioconductor.org/packages/release/bioc/html/CoGAPS.html).

Projection analyses:

(https://www.bioconductor.org/packages/release/bioc/html/projectR.html).

Multi-omics data at NeMO Analytics:

Single gene query:

https://nemoanalytics.org/p?l=Kim2024.

Projection of GWCoGAPS-I:

https://nemoanalytics.org/p?p=p&l=Kim2024&c=Kim2024_GWCoGAPS_I_p24&algo=nmf.

Projection of GWCoGAPS-II:

https://nemoanalytics.org/p?p=p&l=Kim2024&c=Kim2024_GWCoGAPS_II_p30&algo=nmf.

Projection of NextGen Consortium PCA:

https://nemoanalytics.org/p?p=p&l=Kim2024&c=Kim2024_NextGenPCs&algo=pca.

### Methods

#### hPSC culture and differentiation

hPSCs were dissociated with Accutase (A11105, Life Technologies), plated at 1 × 10^5^ cells/cm^2^ on Matrigel (354277, BD)-coated plates, and cultured in mTeSR1 (05850, Stem Cell Technology) with 5 μM Y27632 (Y0503, Sigma-Aldrich), which was removed after 24 h. Neuroectodermal differentiation was induced with Noggin (500 ng/mL, 719-NG, R&D Systems) and SB431542 (2 μM, S4317, Sigma-Aldrich) in mTeSR1; mesendodermal differentiation was induced with BMP4 (100 ng/mL, 314-BP, R&D Systems) upon Y27632 removal (D0) and cultured for 6 days.

#### Generation of CRISPR-Cas9-mediated SOX21-KO line

SOX21-KO lines were generated using CRISPR-Cas9. Oligonucleotides were cloned into pSpCas9(BB)-2A-Puro (px459; Addgene), producing plasmid pX459-Sox21NHEJ4 and pX459-Sox21NHEJ5, synthesized by Integrated DNA Technologies. SA01 hESCs were transfected with 2.5 μg of either plasmid using DNA-In Stem (MTI-GlobalStem, gifted from Dr. Jessee).

#### RNA-seq data processing

After sequencing, Illumina Real Time Analysis (RTA) module was used to perform image analysis and base calling, and BCL Converter (CASAVA v1.8.2) was used to generate FASTQ files. Sequencing depth was over 80 million (40 million paired-end) (Table S1). Read-level Q/C was performed by FastQC (v0.10.1). Pair-end reads of cDNA sequences were aligned back to the human genome (UCSC hg19 from Illumina iGenome) by spliced read mapper TopHat (v2.0.4). Reads were counted by htseq-count v0.5.3 according to gene annotation (Illumina iGenome), and Reads Per Kilobase per Million mapped reads (RPKM) was calculated. This provided 23,368 gene-level expression profiles.

## Supplemental information

Document S1. Supplemental experimental procedures and Figures S1–S6

Table S1. Information on hPSC lines, RNA-seq data, and individual gene amplitudes for GWCoGAPS-I patterns, related to Figure 2 Sheet 1: information on hPSC lines used, Sheet 2: summary of experimental times and conditions for RNA-seq data generation, Sheet 3: mapping and annotation details of RNA-seq, Sheet 4: individual gene amplitudes for GWCoGAPS-I patterns, Sheet 5: list of top 100 genes in 22 GWCoGAPS-I patterns.

Table S2. Individual gene amplitudes for GWCoGAPS-II and genes ranked by their contribution to the line- and donor-specific patterns, related to Figure 5 Submitting the top 250 genes from the line- and donor-specific patterns to the Enrichr webtool (https://maayanlab.cloud/Enrichr/enrich?dataset=3f14ebb0f1889f3a5acb6d940326bebb; https://maayanlab.cloud/Enrichr/enrich?dataset=cb12b784682d4aed17f5b17d6cdbee3b) revealed enrichment in (1) coexpression with many KRAB-ZNF genes (p = 7.8e−9 to p = 5.8e−6) from the ARCHS4 database, (2) H3K9me3 (p = 1.8e−19 to p = 6.4e−7) and SETDB1 (p = 9.7e−8) ChIP-seq peaks from the ENCODE Histone modifications and TF ChIP-seq databases, and (3) TRIM28/KAP1 protein-protein interactions from the BioPlex database (p = 6.8e−6). These enrichments represent a canonical mechanism of transcriptional repression: KRAB-ZNF genes bind repetitive genomic DNA derived from TEs and TRIM28/KAP1, recruiting SETDB1 to deposit the H3K9me3. We also found enrichment of protocadherin (PCDH) genes among the GWCoGAPS gene amplitudes in line-and donor-specific patterns (p = 2e−17 and p = 0.00046, in GWCoGAPS-I and -II, respectively). Y chromosome genes in these lists serve as a control positive in that their expression patterns are described primarily as a combination of the male line-specific patterns. Genes in line- and donor-specific expression patterns can be explored in our NeMO Analytics portal, where examples of both epigenetic (e.g., ZNF genes in Figures S5I and S5J) and genetic (e.g., NOMO3) regulation can be observed in our hPSC lines and the NextGen Consortium lines: https://nemoanalytics.org/p?l=Kim2024&g=NOMO3.

Table S3. Normalized H3K9me3 levels in 2053-2 and 2053-6 lines in self-renewal, related to Figure 5 In particular, GBX2 and other hindbrain fate genes IRX1/2/4, ZIC1, and OLIG3 were highly represented in the 2053-6 line-specific signature and showed higher H3K9me3 levels in 2053-2. Although HOX genes were not expressed in SR condition, 28 HOX genes were among the top 100 with increased H3K9me3 levels in line 2053-2.

Table S4. Gene sets used to explore pluripotency states and anterior-to-posterior bias in NextGen hiPSC library dataset, related to Figure 6 Sheet 1: gene weights of PC3 of mouse mid-streak embryo data. Sheet 2: values of retinoic acid response genes in PC1 and PC2 of NextGen RNA-seq data. Sheet 3: gene weights of PC1 of mouse ESC and EpiSC data.

Document S2. Article plus supplemental information

## References

[bib1] Andrews P.W., Barbaric I., Benvenisty N., Draper J.S., Ludwig T., Merkle F.T., Sato Y., Spits C., Stacey G.N., Wang H., Pera M.F. (2022). The consequences of recurrent genetic and epigenetic variants in human pluripotent stem cells. Cell Stem Cell.

[bib2] Arnold S.J., Robertson E.J. (2009). Making a commitment: cell lineage allocation and axis patterning in the early mouse embryo. Nat. Rev. Mol. Cell Biol..

[bib3] Balmer J.E., Blomhoff R. (2002). Gene expression regulation by retinoic acid. J. Lipid Res..

[bib4] Bao S., Tang F., Li X., Hayashi K., Gillich A., Lao K., Surani M.A. (2009). Epigenetic reversion of post-implantation epiblast to pluripotent embryonic stem cells. Nature.

[bib5] Bonder M.J., Smail C., Gloudemans M.J., Frésard L., Jakubosky D., D'Antonio M., Li X., Ferraro N.M., Carcamo-Orive I., Mirauta B. (2021). Identification of rare and common regulatory variants in pluripotent cells using population-scale transcriptomics. Nat. Genet..

[bib6] Brons I.G.M., Smithers L.E., Trotter M.W.B., Rugg-Gunn P., Sun B., Chuva de Sousa Lopes S.M., Howlett S.K., Clarkson A., Ahrlund-Richter L., Pedersen R.A., Vallier L. (2007). Derivation of pluripotent epiblast stem cells from mammalian embryos. Nature.

[bib7] Buckberry S., Liu X., Poppe D., Tan J.P., Sun G., Chen J., Nguyen T.V., de Mendoza A., Pflueger J., Frazer T. (2023). Transient naive reprogramming corrects hiPS cells functionally and epigenetically. Nature.

[bib8] Burke E.E., Chenoweth J.G., Shin J.H., Collado-Torres L., Kim S.K., Micali N., Wang Y., Colantuoni C., Straub R.E., Hoeppner D.J. (2020). Dissecting transcriptomic signatures of neuronal differentiation and maturation using iPSCs. Nat. Commun..

[bib9] Carcamo-Orive I., Hoffman G.E., Cundiff P., Beckmann N.D., D'Souza S.L., Knowles J.W., Patel A., Papatsenko D., Abbasi F., Reaven G.M. (2017). Analysis of Transcriptional Variability in a Large Human iPSC Library Reveals Genetic and Non-genetic Determinants of Heterogeneity. Cell Stem Cell.

[bib10] Chambers S.M., Fasano C.A., Papapetrou E.P., Tomishima M., Sadelain M., Studer L. (2009). Highly efficient neural conversion of human ES and iPS cells by dual inhibition of SMAD signaling. Nat. Biotechnol..

[bib11] Choi J., Lee S., Mallard W., Clement K., Tagliazucchi G.M., Lim H., Choi I.Y., Ferrari F., Tsankov A.M., Pop R. (2015). A comparison of genetically matched cell lines reveals the equivalence of human iPSCs and ESCs. Nat. Biotechnol..

[bib12] Collins R.L., Glessner J.T., Porcu E., Lepamets M., Brandon R., Lauricella C., Han L., Morley T., Niestroj L.M., Ulirsch J. (2022). A cross-disorder dosage sensitivity map of the human genome. Cell.

[bib13] Cuomo A.S.E., Seaton D.D., McCarthy D.J., Martinez I., Bonder M.J., Garcia-Bernardo J., Amatya S., Madrigal P., Isaacson A., Buettner F. (2020). Single-cell RNA-sequencing of differentiating iPS cells reveals dynamic genetic effects on gene expression. Nat. Commun..

[bib14] DeBoever C., Li H., Jakubosky D., Benaglio P., Reyna J., Olson K.M., Huang H., Biggs W., Sandoval E., D'Antonio M. (2017). Large-Scale Profiling Reveals the Influence of Genetic Variation on Gene Expression in Human Induced Pluripotent Stem Cells. Cell Stem Cell.

[bib15] Ecco G., Cassano M., Kauzlaric A., Duc J., Coluccio A., Offner S., Imbeault M., Rowe H.M., Turelli P., Trono D. (2016). Transposable Elements and Their KRAB-ZFP Controllers Regulate Gene Expression in Adult Tissues. Dev. Cell.

[bib16] Factor D.C., Corradin O., Zentner G.E., Saiakhova A., Song L., Chenoweth J.G., McKay R.D., Crawford G.E., Scacheri P.C., Tesar P.J. (2014). Epigenomic Comparison Reveals Activation of "Seed" Enhancers during Transition from Naive to Primed Pluripotency. Cell Stem Cell.

[bib17] Faial T., Bernardo A.S., Mendjan S., Diamanti E., Ortmann D., Gentsch G.E., Mascetti V.L., Trotter M.W.B., Smith J.C., Pedersen R.A. (2015). Brachyury and SMAD signalling collaboratively orchestrate distinct mesoderm and endoderm gene regulatory networks in differentiating human embryonic stem cells. Development.

[bib18] Fang Z., Liu X., Wen J., Tang F., Zhou Y., Jing N., Jin Y. (2019). SOX21 Ensures Rostral Forebrain Identity by Suppression of WNT8B during Neural Regionalization of Human Embryonic Stem Cells. Stem Cell Rep..

[bib19] Ferrell J.E. (2012). Bistability, bifurcations, and Waddington's epigenetic landscape. Curr. Biol..

[bib20] Ghyselinck N.B., Duester G. (2019). Retinoic acid signaling pathways. Development.

[bib21] Gogolou A., Souilhol C., Granata I., Wymeersch F.J., Manipur I., Wind M., Frith T.J.R., Guarini M., Bertero A., Bock C. (2022). Early anteroposterior regionalisation of human neural crest is shaped by a pro-mesodermal factor. Elife.

[bib22] Goolam M., Scialdone A., Graham S.J.L., Macaulay I.C., Jedrusik A., Hupalowska A., Voet T., Marioni J.C., Zernicka-Goetz M. (2016). Heterogeneity in Oct4 and Sox2 Targets Biases Cell Fate in 4-Cell Mouse Embryos. Cell.

[bib23] GTEx Consortium (2015). The Genotype-Tissue Expression (GTEx) pilot analysis: Multitissue gene regulation in humans. Science.

[bib24] Guibentif C., Griffiths J.A., Imaz-Rosshandler I., Ghazanfar S., Nichols J., Wilson V., Göttgens B., Marioni J.C. (2021). Diverse Routes toward Early Somites in the Mouse Embryo. Dev. Cell.

[bib25] Guo G., Stirparo G.G., Strawbridge S.E., Spindlow D., Yang J., Clarke J., Dattani A., Yanagida A., Li M.A., Myers S. (2021). Human naive epiblast cells possess unrestricted lineage potential. Cell Stem Cell.

[bib26] Henrique D., Abranches E., Verrier L., Storey K.G. (2015). Neuromesodermal progenitors and the making of the spinal cord. Development.

[bib27] Hoetker M.S., Yagi M., Di Stefano B., Langerman J., Cristea S., Wong L.P., Huebner A.J., Charlton J., Deng W., Haggerty C. (2023). H3K36 methylation maintains cell identity by regulating opposing lineage programmes. Nat. Cell Biol..

[bib28] Hough S.R., Thornton M., Mason E., Mar J.C., Wells C.A., Pera M.F. (2014). Single-Cell Gene Expression Profiles Define Self-Renewing, Pluripotent, and Lineage Primed States of Human Pluripotent Stem Cells. Stem Cell Rep..

[bib29] Jaffe A.E., Straub R.E., Shin J.H., Tao R., Gao Y., Collado-Torres L., Kam-Thong T., Xi H.S., Quan J., Chen Q. (2018). Developmental and genetic regulation of the human cortex transcriptome illuminate schizophrenia pathogenesis. Nat. Neurosci..

[bib30] Jerber J., Seaton D.D., Cuomo A.S.E., Kumasaka N., Haldane J., Steer J., Patel M., Pearce D., Andersson M., Bonder M.J. (2021). Population-scale single-cell RNA-seq profiling across dopaminergic neuron differentiation. Nat. Genet..

[bib31] Jourdon A., Wu F., Mariani J., Capauto D., Norton S., Tomasini L., Amiri A., Suvakov M., Schreiner J.D., Jang Y. (2023). Modeling idiopathic autism in forebrain organoids reveals an imbalance of excitatory cortical neuron subtypes during early neurogenesis. Nat. Neurosci..

[bib32] Kanton S., Boyle M.J., He Z., Santel M., Weigert A., Sanchís-Calleja F., Guijarro P., Sidow L., Fleck J.S., Han D. (2019). Organoid single-cell genomic atlas uncovers human-specific features of brain development. Nature.

[bib33] Kilpinen H., Goncalves A., Leha A., Afzal V., Alasoo K., Ashford S., Bala S., Bensaddek D., Casale F.P., Culley O.J. (2017). Common genetic variation drives molecular heterogeneity in human iPSCs. Nature.

[bib34] Kuzmichev A.N., Kim S.K., D'Alessio A.C., Chenoweth J.G., Wittko I.M., Campanati L., McKay R.D. (2012). Sox2 acts through Sox21 to regulate transcription in pluripotent and differentiated cells. Curr. Biol..

[bib35] Kyttala A., Moraghebi R., Valensisi C., Kettunen J., Andrus C., Pasumarthy K.K., Nakanishi M., Nishimura K., Ohtaka M., Weltner J. (2016). Genetic Variability Overrides the Impact of Parental Cell Type and Determines iPSC Differentiation Potential. Stem Cell Rep..

[bib36] Mallon B.S., Chenoweth J.G., Johnson K.R., Hamilton R.S., Tesar P.J., Yavatkar A.S., Tyson L.J., Park K., Chen K.G., Fann Y.C., McKay R.D.G. (2013). StemCellDB: the human pluripotent stem cell database at the National Institutes of Health. Stem Cell Res..

[bib37] Mariani J., Coppola G., Zhang P., Abyzov A., Provini L., Tomasini L., Amenduni M., Szekely A., Palejev D., Wilson M. (2015). FOXG1-Dependent Dysregulation of GABA/Glutamate Neuron Differentiation in Autism Spectrum Disorders. Cell.

[bib38] Matsuda S., Kuwako K.i., Okano H.J., Tsutsumi S., Aburatani H., Saga Y., Matsuzaki Y., Akaike A., Sugimoto H., Okano H. (2012). Sox21 promotes hippocampal adult neurogenesis via the transcriptional repression of the Hes5 gene. J. Neurosci..

[bib39] Merkle F.T., Ghosh S., Genovese G., Handsaker R.E., Kashin S., Meyer D., Karczewski K.J., O'Dushlaine C., Pato C., Pato M. (2022). Whole-genome analysis of human embryonic stem cells enables rational line selection based on genetic variation. Cell Stem Cell.

[bib40] Micali N., Kim S.-K., Diaz-Bustamante M., Stein-O’Brien G., Seo S., Shin J.-H., Rash B.G., Ma S., Wang Y., Olivares N.A. (2020). Variation of Human Neural Stem Cells Generating Organizer States In Vitro before Committing to Cortical Excitatory or Inhibitory Neuronal Fates. Cell Rep..

[bib41] Millet S., Campbell K., Epstein D.J., Losos K., Harris E., Joyner A.L. (1999). A role for Gbx2 in repression of Otx2 and positioning the mid/hindbrain organizer. Nature.

[bib42] Nakanishi M., Mitchell R.R., Benoit Y.D., Orlando L., Reid J.C., Shimada K., Davidson K.C., Shapovalova Z., Collins T.J., Nagy A., Bhatia M. (2019). Human Pluripotency Is Initiated and Preserved by a Unique Subset of Founder Cells. Cell.

[bib43] Ortmann D., Brown S., Czechanski A., Aydin S., Muraro D., Huang Y., Tomaz R.A., Osnato A., Canu G., Wesley B.T. (2020). Naive Pluripotent Stem Cells Exhibit Phenotypic Variability that Is Driven by Genetic Variation. Cell Stem Cell.

[bib44] Paulsen B., Velasco S., Kedaigle A.J., Pigoni M., Quadrato G., Deo A.J., Adiconis X., Uzquiano A., Sartore R., Yang S.M. (2022). Autism genes converge on asynchronous development of shared neuron classes. Nature.

[bib45] Peng G., Suo S., Chen J., Chen W., Liu C., Yu F., Wang R., Chen S., Sun N., Cui G. (2016). Spatial Transcriptome for the Molecular Annotation of Lineage Fates and Cell Identity in Mid-gastrula Mouse Embryo. Dev. Cell.

[bib46] Pera M.F. (2023). Seven days in the life cycle of Homo sapiens. Cell.

[bib47] Pijuan-Sala B., Griffiths J.A., Guibentif C., Hiscock T.W., Jawaid W., Calero-Nieto F.J., Mulas C., Ibarra-Soria X., Tyser R.C.V., Ho D.L.L. (2019). A single-cell molecular map of mouse gastrulation and early organogenesis. Nature.

[bib48] Puigdevall P., Jerber J., Danecek P., Castellano S., Kilpinen H. (2023). Somatic mutations alter the differentiation outcomes of iPSC-derived neurons. Cell Genom..

[bib49] Rifes P., Isaksson M., Rathore G.S., Aldrin-Kirk P., Møller O.K., Barzaghi G., Lee J., Egerod K.L., Rausch D.M., Parmar M. (2020). Modeling neural tube development by differentiation of human embryonic stem cells in a microfluidic WNT gradient. Nat. Biotechnol..

[bib50] Rosebrock D., Arora S., Mutukula N., Volkman R., Gralinska E., Balaskas A., Aragonés Hernández A., Buschow R., Brändl B., Müller F.J. (2022). Enhanced cortical neural stem cell identity through short SMAD and WNT inhibition in human cerebral organoids facilitates emergence of outer radial glial cells. Nat. Cell Biol..

[bib51] Rouhani F., Kumasaka N., de Brito M.C., Bradley A., Vallier L., Gaffney D. (2014). Genetic background drives transcriptional variation in human induced pluripotent stem cells. PLoS Genet..

[bib52] Shibata M., Pattabiraman K., Lorente-Galdos B., Andrijevic D., Kim S.K., Kaur N., Muchnik S.K., Xing X., Santpere G., Sousa A.M.M., Sestan N. (2021). Regulation of prefrontal patterning and connectivity by retinoic acid. Nature.

[bib53] Stein-O'Brien G.L., Carey J.L., Lee W.S., Considine M., Favorov A.V., Flam E., Guo T., Li S., Marchionni L., Sherman T. (2017). PatternMarkers & GWCoGAPS for novel data-driven biomarkers via whole transcriptome NMF. Bioinformatics.

[bib54] Strano A., Tuck E., Stubbs V.E., Livesey F.J. (2020). Variable Outcomes in Neural Differentiation of Human PSCs Arise from Intrinsic Differences in Developmental Signaling Pathways. Cell Rep..

[bib55] Tesar P.J., Chenoweth J.G., Brook F.A., Davies T.J., Evans E.P., Mack D.L., Gardner R.L., McKay R.D.G. (2007). New cell lines from mouse epiblast share defining features with human embryonic stem cells. Nature.

[bib56] Wang M., Wei P.C., Lim C.K., Gallina I.S., Marshall S., Marchetto M.C., Alt F.W., Gage F.H. (2020). Increased Neural Progenitor Proliferation in a hiPSC Model of Autism Induces Replication Stress-Associated Genome Instability. Cell Stem Cell.

[bib57] Warmflash A., Sorre B., Etoc F., Siggia E.D., Brivanlou A.H. (2014). A method to recapitulate early embryonic spatial patterning in human embryonic stem cells. Nat. Methods.

[bib58] Zeng B., Liu Z., Lu Y., Zhong S., Qin S., Huang L., Zeng Y., Li Z., Dong H., Shi Y. (2023). The single-cell and spatial transcriptional landscape of human gastrulation and early brain development. Cell Stem Cell.

[bib59] Zhai J., Guo J., Wan H., Qi L., Liu L., Xiao Z., Yan L., Schmitz D.A., Xu Y., Yu D. (2022). Primate gastrulation and early organogenesis at single-cell resolution. Nature.

