# Supplementary material for: Individual variation in the emergence of anterior-to-posterior neural fates from human pluripotent stem cells
Source: Stem Cell Reports. 2024 Aug 15;19(9):1336–50. doi: 10.1016/j.stemcr.2024.07.004 (PMC11411333; doi:10.1016/j.stemcr.2024.07.004)
Supplement: Document S1. Supplemental experimental procedures and Figures S1–S6 [file mmc1.pdf]

Stem Cell Reports, Volume *19*

Supplemental Information

Individual variation in the emergence of anterior-to-posterior neural fates from human pluripotent stem cells

Suel-Kee Kim, Seungmae Seo, Genevieve Stein-O'Brien, Amritha Jaishankar, Kazuya Ogawa, Nicola Micali, Victor Luria, Amir Karger, Yanhong Wang, Hyojin Kim, Thomas M. Hyde, Joel E. Kleinman, Ty Voss, Elana J. Fertig, Joo-Heon Shin, Roland Bürli, Alan J. Cross, Nicholas J. Brandon, Daniel R. Weinberger, Joshua G. Chenoweth, Daniel J. Hoeppner, Nenad Sestan, Carlo Colantuoni, and Ronald D. McKay

Supplemental Materials

Supplemental Figures

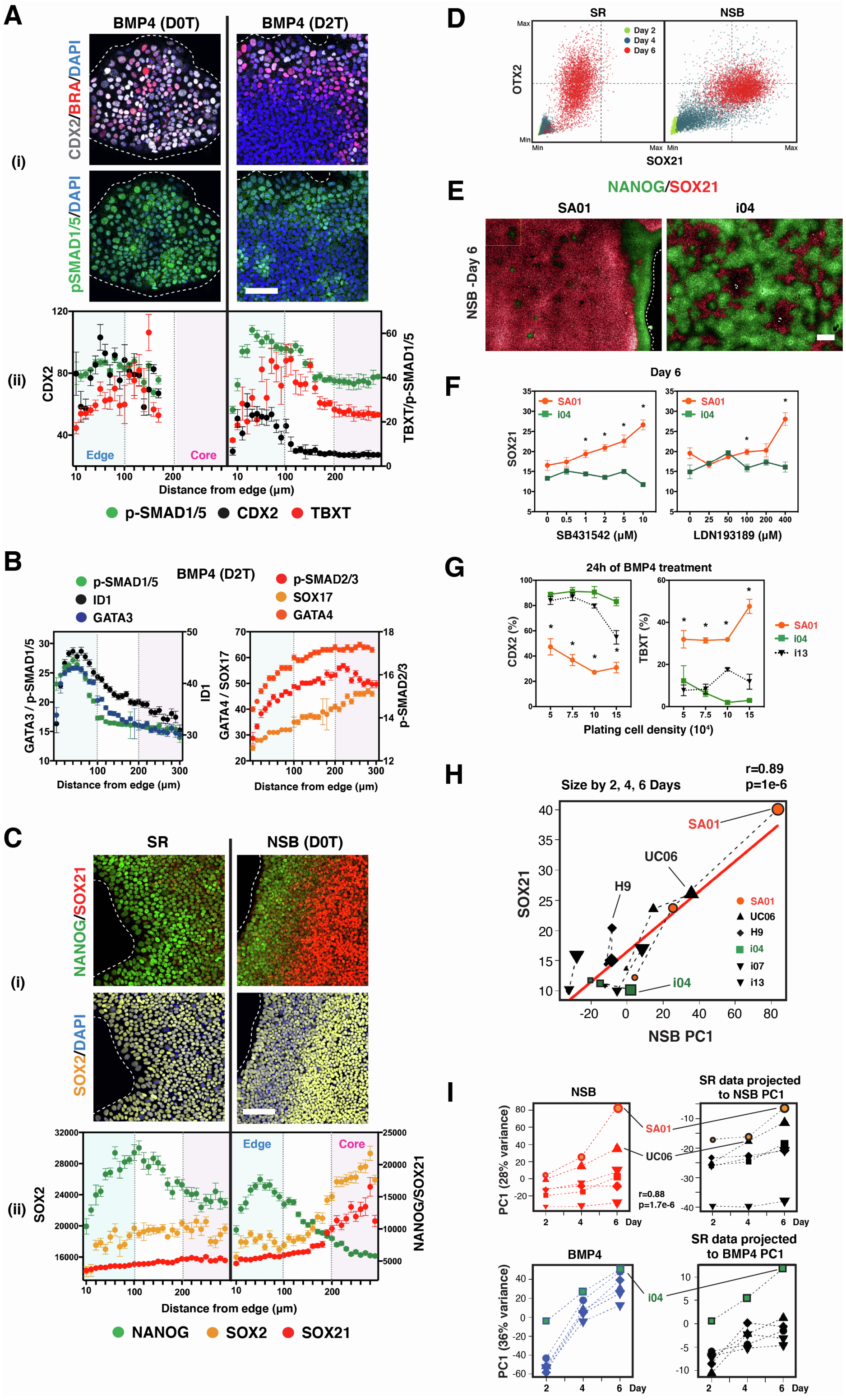

Figure S1. Cell line variation in the emergence of neural fate from pluripotency, related to Figure 1. (**A**) CDX2, TBXT, and pSMAD1/5 levels at 24 hours after BMP4 treatments (D0T: treated on day 0, D2T: treated on day 2). (i) Representative images. Scale bar, 100 μm. (ii) Expression levels plotted against distance from the edge. (**B**) Spatial expression of mesendoderm regulators at 24 hours after BMP4 treatment on day 2 (D2T). (**C**) Spatial expression of NANOG, SOX21, and SOX2 on day 4 in SR and NSB. (i) Representative images. Scale bar, 100 μm. (ii) Spatial expression levels. (**D**) Scatter plots showing single-cell levels of SOX21 and OTX2 expression in self-renewal (SR) and NSB conditions through culture times demonstrate a positive correlation between them during neuroectodermal differentiation. (**E**) SOX21 and NANOG expression on day 6 of NSB treatment in SA01 and i04 lines show a more efficient formation of the core zone in SA01 compared to i04. Scale bar, 200 μm. Dashed lines indicate the edge of colonies. (**F**) Dose-response curve of BMP/TGFβ signaling inhibitors LDN193189 and SB431542 on SOX21 induction on day 6 shows differential responses between SA01 and i04 lines. *, $p<0.05$ between lines. n=3 independent experiments. (**G**) Proportion of CDX2 and TBXT expressing cells in SA01 and i04 lines after 24 h of BMP4 treatment on day 0 (D0T) shows the cell line difference is not caused by initial cell plating density. *, $p<0.05$ between lines. n=3 independent experiments. (**H**) Scatter plot showing the correlation between SOX21 protein levels in NSB (Figure 1B) and NSB PC1. (**I**) Projections of SR data into PC1 of data from differentiation showing cell line-specific transcriptional lineage priming. PC1 of NSB and BMP4 data (left) and projection of SR data into the NSB and BMP4 PC1 (right).

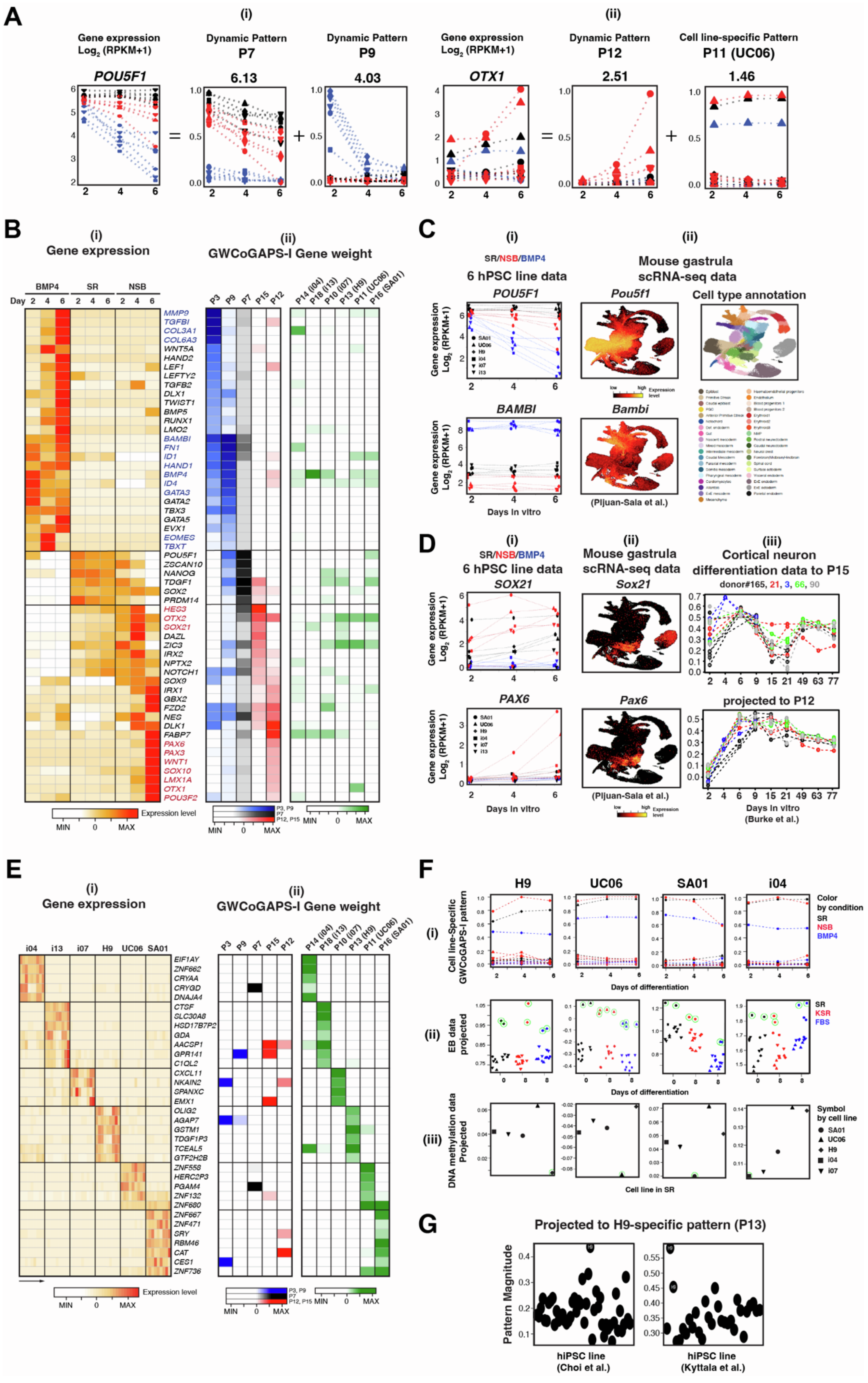
A
(i)
(ii)
Gene expression Log2 (RPKM+1)
Dynamic Pattern P7
Dynamic Pattern P9
Dynamic Pattern P12
Cell line-specific Pattern P11 (UC06)
POU5F1
OTX1
6.13
4.03
2.51
1.46
B
Gene expression
GWCoGAPS-I Gene weight
C
SR/NSB/BMP4
6 hPSC line data
Mouse gastrula scRNA-seq data
Cell type annotation
POU5F1
Pou5f1
BAMBI
Bambi
Days in vitro
(Pijuan-Sala et al.)
D
Cortical neuron differentiation data to P15
SOX21
Sox21
PAX6
Pax6
projected to P12
(Burke et al.)
E
Gene expression
GWCoGAPS-I Gene weight
F
H9
UC06
SA01
i04
Cell line-Specific GWCoGAPS-I pattern
EB data projected
DNA methylation data Projected
Days of differentiation
Cell line in SR
G
Projected to H9-specific pattern (P13)
Pattern Magnitude
hiPSC line (Choi et al.)
hiPSC line (Kyttala et al.)

Figure S2. Decomposing dynamic and cell line-specific transcriptomic modules, related to Figure 2. (**A**) The use of each GWCoGAPS pattern across genes can be precisely defined by gene-specific amplitudes for all patterns (Table S1). Two examples of individual genes whose expression patterns are represented by the combination of multiple GWCoGAPS patterns are shown. (i) The complete expression of *POU5F1* is represented by two dynamic GWCoGAPS patterns P7 and P9. (ii) The complete expression of *OTX1* is represented by a dynamic GWCoGAPS pattern P12 and UC06 line-specific GWCoGAPS pattern P11. (**B**) Gene expression of pluripotency or differentiation regulators and their weights in dynamic and cell line-specific GWCoGAPS-I patterns. (i) Heatmap of gene expression. Gene expression levels were normalized across the row (gene) to show their expression dynamics in time and condition. (ii) Heatmap showing the gene weights. SR pattern: P7; NSB pattern: P12 and P15; BMP4 pattern: P9 and P3. The A values of the genes were normalized within each column (pattern) to show their representations in each dynamic or cell line-specific pattern. (**C**) GWCoGAPS-I pattern P7 represents the loss of pluripotency, and P3 represents the induction of mesendoderm regulators. (i) Expression of *POU5f1* and *BAMBI*, the top-ranked genes in P7 and P3, respectively, in 6 hPSC line data. (ii) Expression of *Pou5f1* and *Bambi* in mouse gastrula scRNA-seq data(Pijuan-Sala et al., 2019). (**D**) NSB patterns P15 and P12 represent distinct transition steps toward neural fates. (i) Expression of *SOX21* and *PAX6*, the top-ranked genes in P15 and P12, respectively, in 6 hPSC line data. (ii) Expression of *Sox21* and *Pax6* in mouse gastrula scRNA-seq data. (iii) Projection of cortical neuron differentiation data (Burke et al., 2020) from multiple hiPSC lines. (**E**) Expression of cell line-specific genes exhibiting the highest weights in each line-specific pattern and their weights in dynamic and 6 cell line-specific patterns. (i) Heatmap of gene expression. Gene expression levels were normalized across the row (gene) to show their expression across the time, condition, and cell lines. The arrow indicates the order from left to right: 2, 4, and 6 days of SR, BMP4, and NSB conditions. (ii) Heatmap showing the gene weights. The A values of the genes were normalized within each column (pattern) to show their representations in each dynamic or cell line-specific pattern. (**F**) Projections of microarray (Mallon et al., 2013) and DNA methylation data generated from multiple hPSC lines into the cell line-specific GWCoGAPS-I patterns demonstrate the stability of the cell line-specific transcriptional signatures within a cell line. (i) H9, UC06, SA01, and i04 line-specific GWCoGAPS-I patterns that define distinct transcriptional signatures from all other lines across time and condition. The corresponding cell line samples are marked in green circles. (ii) Projections of microarray dataset containing the same 6 lines under embryoid body (EB) differentiation conditions (SR for self-renewal in hESC medium plus FGF2, KSR for ectodermal differentiation, and FBS for mesendodermal differentiation) into each cell line-specific patterns discriminate the corresponding cell line samples from all other lines. (iii) Projections of DNA methylation data show that promoters of genes expressed specifically in each cell line are hypomethylated in the corresponding cell line. (**G**) Projection of multiple hPSC line data (Choi et al., 2015; Kyttala et al., 2016) into H9-specific pattern. H9 samples are circled in green.

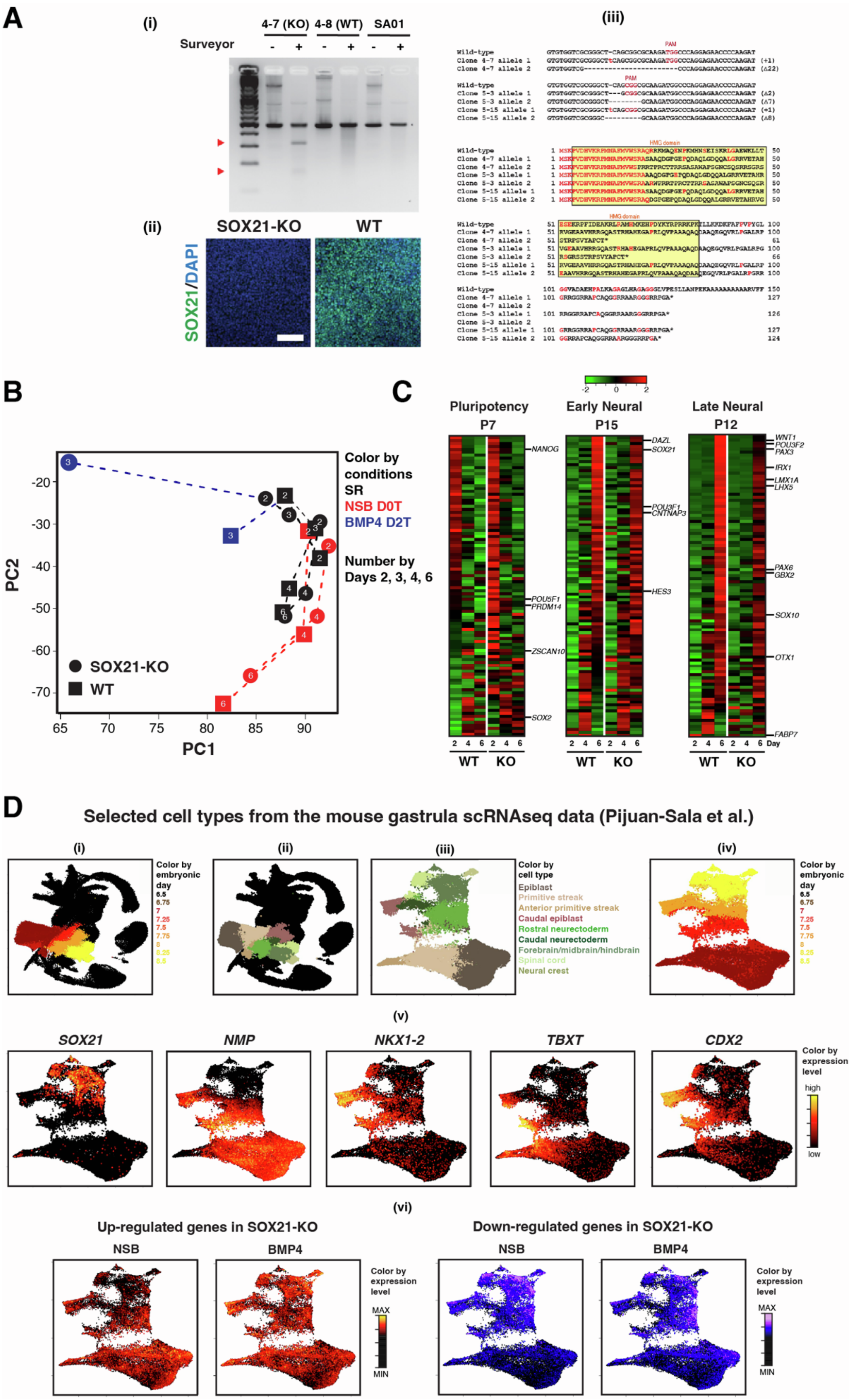

A
(i)
4-7 (KO) 4-8 (WT) SA01
Surveyor
(ii)
SOX21-KO
WT
SOX21/DAPI
(iii)
B
Color by conditions
SR
NSB D0T
BMP4 D2T
Number by Days 2, 3, 4, 6
SOX21-KO
WT
PC1
PC2
C
Pluripotency P7
Early Neural P15
Late Neural P12
WT KO
D
Selected cell types from the mouse gastrula scRNAseq data (Pijuan-Sala et al.)
Color by embryonic day
Color by cell type
Epiblast
Primitive streak
Anterior primitive streak
Caudal epiblast
Rostral neurectoderm
Caudal neurectoderm
Forebrain/midbrain/hindbrain
Spinal cord
Neural crest
SOX21
NMP
NKX1-2
TBXT
CDX2
Color by expression level
Up-regulated genes in SOX21-KO
Down-regulated genes in SOX21-KO
NSB
BMP4

Figure S3. SOX21 regulates early forebrain fate choice, related to Figure 3. (**A**) Establishment of SOX21-KO lines by CRISPR/Cas9 technology. All SOX21-KO SA01 hESC lines were screened using Surveyor and immunofluorescence assays and verified by DNA sequencing. (i) Analysis of SOX21-KO clones using Surveyor assay. The gel image shows modification at the SOX21 locus in a clone 4-7. Red arrowheads indicate expected fragment sizes for the SOX21 locus. (ii) Immunostaining of WT and SOX21-KO clones shows complete loss of SOX21 expression in clone 4-7. The cells were cultured in the presence of NSB for 6 days. Scale bar, 100 µm. (iii) Amino acid sequence of SOX21 alterations by CRISPR/Cas9 confirms knockout of SOX21. In three clones (clones 4-7, 5-3, and 5-15), frameshift mutation, premature stop codon mutation, or mutation that disrupts the HMG domain were confirmed in both *SOX21* alleles. These three clones were used for the functional assays shown in Figure 3. (**B**) Projection of WT and SOX21-KO line data into PC1 and PC2 of days 2, 4, and 6 data (Figure 1C) reveals delayed neuroectodermal differentiation in NSB and accelerated early mesendodermal differentiation under BMP4 D2T in SOX21-KO compared to WT. (**C**) Heatmaps showing the top 100 genes from P7, P15, and P12 (Table S1) in NSB. (**D**) Uniform manifold approximation and projection (UMAP) plots of selected epiblast and neural cell populations in mouse gastrula RNA-seq data (Pijuan-Sala *et al.*, 2019) showing *SOX21* and neuromesodermal precursor (NMP) gene expression. (i) A UMAP plot showing all cells with selected cell types colored by embryonic days. (ii) A UMAP plot showing all cells colored by cell type annotation with selected cell types (iii) A UMAP plot showing the selected cell types only and their cell type annotation. (iv) A UMAP plot showing the selected cell types colored by embryonic days. (v) Expression of *Sox21* and average expression of the neuromesodermal progenitor (NMP) genes in the epiblast and early neural lineage cells in mouse gastrula RNA-seq data. *Nkx1-2* expression delineates caudal epiblast cells showing the NMP gene signature, while *Tbxt* expression represents both the caudal epiblast cells and anterior primitive streak cells. Note the mutually exclusive expression between *Sox21* in the rostral neuroectoderm and *Cdx2* in caudal epiblast at developmental day 7.75, showing the early role of SOX21 in anterior-to-posterior axis patterning. (vi) A UMAP plot showing the projection of log fold changes of up- or down-regulated genes from the SOX21-KO RNA-seq data into the mouse gastrula RNA-seq data. Color scales range from the maximum to the minimum of the projected values within an analysis for each panel. The projected values are comparable within a panel but not between panels.

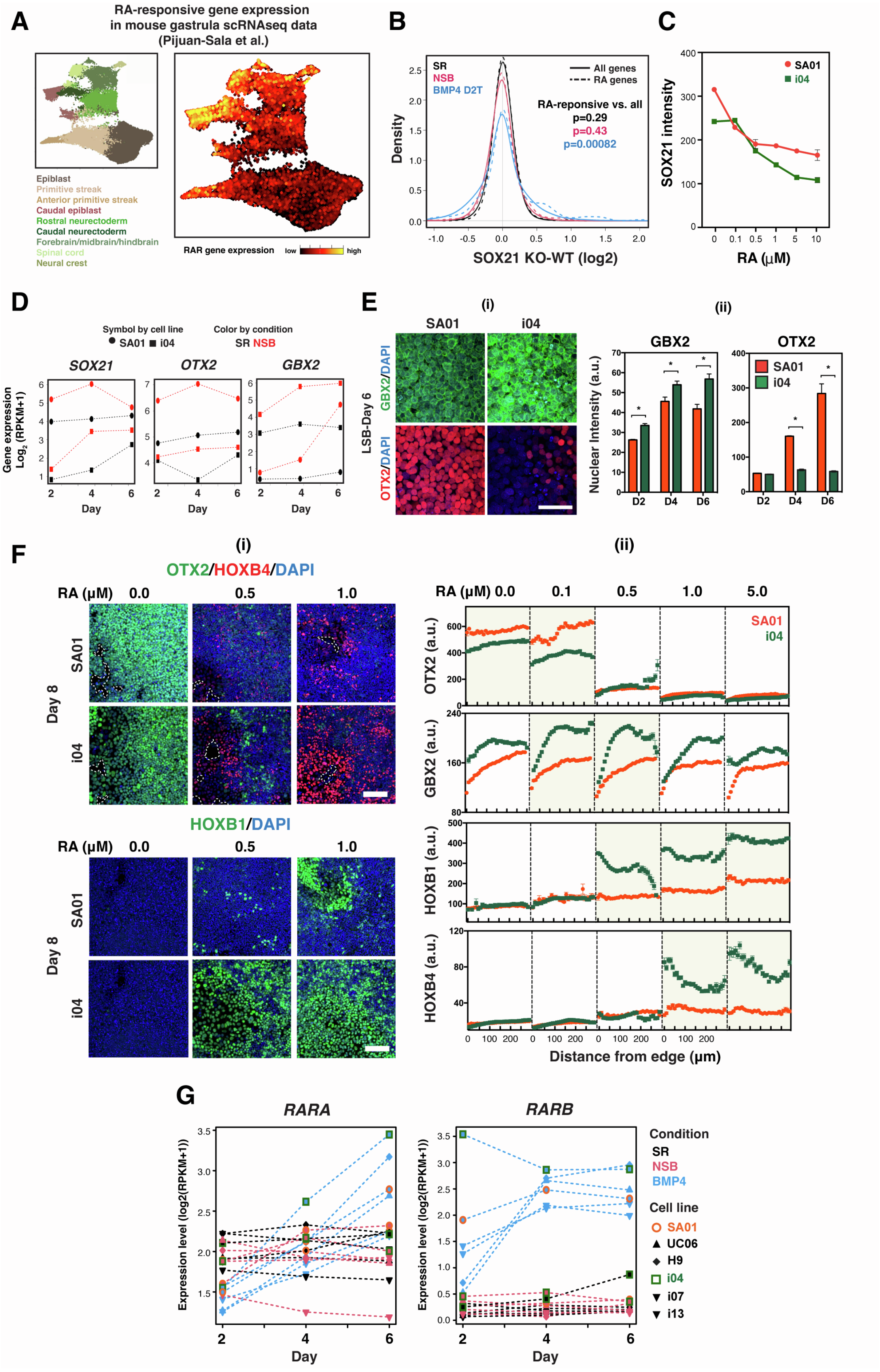
A
RA-responsive gene expression in mouse gastrula scRNAseq data (Pijuan-Sala et al.)
Epiblast
Primitive streak
Anterior primitive streak
Caudal epiblast
Rostral neurectoderm
Caudal neurectoderm
Forebrain/midbrain/hindbrain
Spinal cord
Neural crest
RAR gene expression
low
high
B
SR
NSB
BMP4 D2T
All genes
RA genes
RA-reponsive vs. all
p=0.29
p=0.43
p=0.00082
Density
SOX21 KO-WT (log2)
C
SA01
i04
SOX21 intensity
RA (μM)
D
Symbol by cell line
SA01
i04
Color by condition
SR NSB
SOX21
OTX2
GBX2
Gene expression Log2 (RPKM+1)
Day
E
(i)
(ii)
SA01
i04
LSB-Day 6
GBX2/DAPI
OTX2/DAPI
GBX2
OTX2
Nuclear Intensity (a.u.)
D2
D4
D6
F
(i)
(ii)
OTX2/HOXB4/DAPI
RA (μM)
0.0
0.5
1.0
Day 8
SA01
i04
HOXB1/DAPI
RA (μM) 0.0
0.1
0.5
1.0
5.0
OTX2 (a.u.)
GBX2 (a.u.)
HOXB1 (a.u.)
HOXB4 (a.u.)
Distance from edge (μm)
G
RARA
RARB
Expression level (log2(RPKM+1))
Day
Condition
SR
NSB
BMP4
Cell line
SA01
UC06
H9
i04
i07
i13

Figure S4. Cell line variation in fore- vs. hind-brain fate bias, related to Figure 4. (**A**) A UMAP of the selected populations from the mouse gastrula scRNA-seq data colored by retinoic acid (RA)-responsive gene expression. (**B**) A density plot of all 252 RA-responsive genes (dotted lines) compared to all 21,022 genes (solid lines) in SR, NSB, and BMP4 D2T conditions of SOX21-KO RNA-seq data. (**C**) Dose-dependent reduction of SOX21 protein expression level in SA01 and i04 lines after RA treatment in neuroectoderm condition. n=3 technical replicates. (**D**) mRNA expression of *OTX2*, *GBX2*, and *SOX21* from the hiPSC RNA-seq data. (**E**) Differential GBX2 and OTX2 expression in SA01 and i04. (i) Representative images on day 6 in neuroectoderm differentiation conditions (LDN193189 and SB431542, LSB). Scale bar, 50 μm. (ii) OTX2 and GBX2 expression level in SR. *, Comparison between SA01 and i04 ($p<0.05$). n=3 technical replicates. (**F**) Dose-response analysis of RA shows differential posterization of neural precursors between SA01 and i04 lines. (i) Representative images show differential OTX2, HOXB4, and HOXB1 expression on day 8 of LSB+RA-induced differentiation between SA01 and i04 lines. Scale bar, 100 μm. Dashed lines indicate the edge of colonies. (ii) Dose-response analysis of RA on spatial expression of anterior-posterior neural regulators shows a sequential posteriorization from the core to edge zones and a cell line-specific RA response. Single-cell levels of OTX2, GBX2, HOXB1, and HOXB4 expression are measured on day 8 of LSB+RA-induced differentiation and plotted against the distance from the edge. In contrast, HOXB9 and HOXD9, markers of the posterior spinal cord and trunk neural crest, were not detected in RA-treated cells (data not shown). (**G**) Gene expression of *RARA* and *RARB* in 6 PSC lines across different conditions.

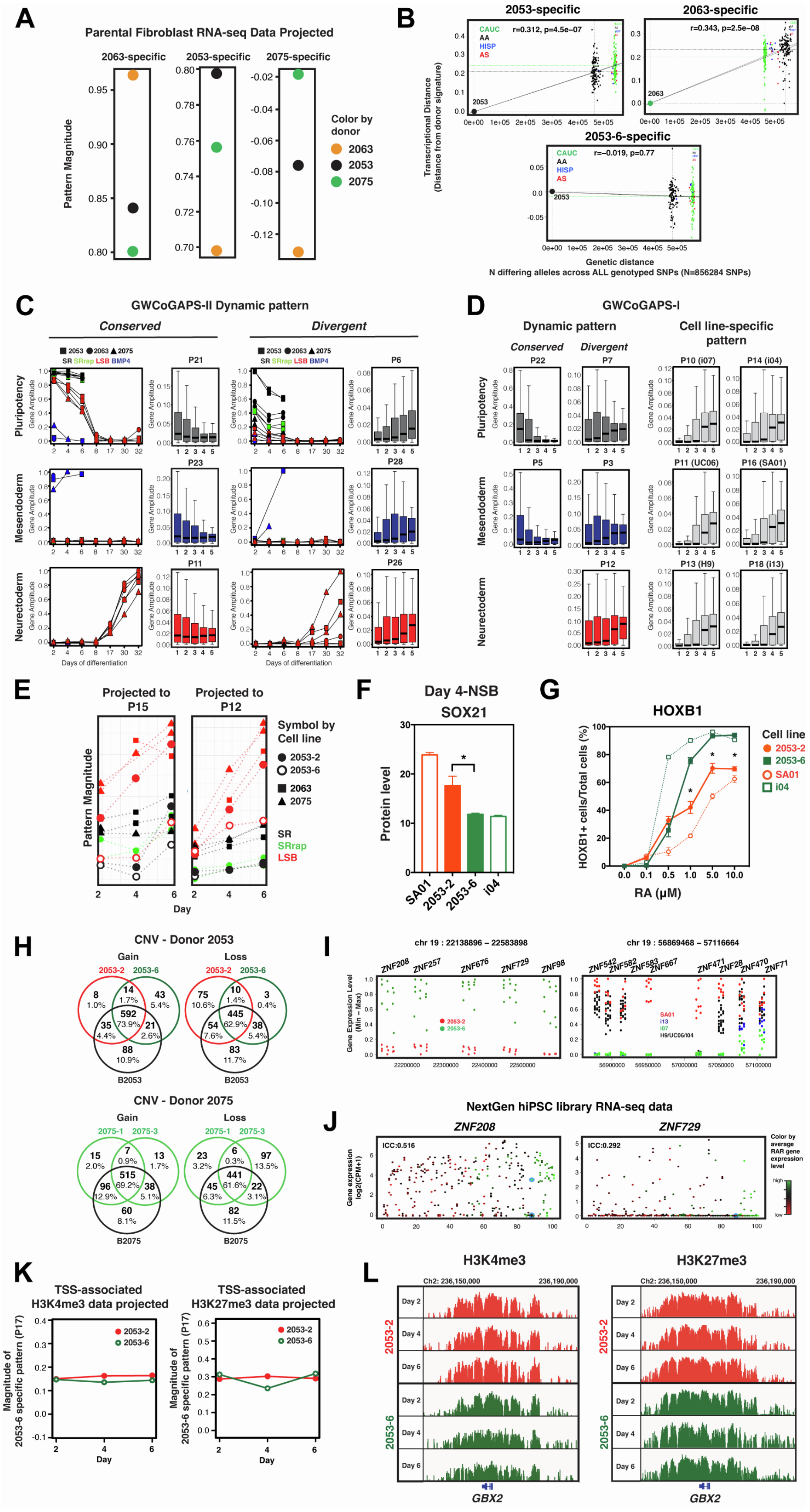

A
Parental Fibroblast RNA-seq Data Projected
2063-specific
2053-specific
2075-specific
Pattern Magnitude
Color by donor
2063
2053
2075
B
2053-specific
2063-specific
2053-6-specific
Transcriptional Distance (Distance from donor signature)
Genetic distance
N differing alleles across ALL genotyped SNPs (N=856284 SNPs)
C
GWCoGAPS-II Dynamic pattern
Conserved
Divergent
Pluripotency
Mesendoderm
Neurectoderm
Days of differentiation
D
GWCoGAPS-I
Dynamic pattern
Cell line-specific pattern
E
Projected to P15
Projected to P12
Symbol by Cell line
Pattern Magnitude
Day
F
Day 4-NSB
SOX21
Protein level
G
HOXB1
HOXB1+ cells/Total cells (%)
RA (μM)
Cell line
H
CNV - Donor 2053
CNV - Donor 2075
I
chr 19 : 22138896 – 22583898
chr 19 : 56869468 – 57116664
Gene Expression Level (Min - Max)
J
NextGen hiPSC library RNA-seq data
ZNF208
ZNF729
Gene expression log2(CPM+1)
K
TSS-associated H3K4me3 data projected
TSS-associated H3K27me3 data projected
Day
L
H3K4me3
H3K27me3
GBX2

Figure S5. Genetic and epigenetic elements contribute to donor- and line-specific transcriptomic signatures, related to Figure 5. (**A**) Projection of parental fibroblast RNA-seq data into donor-specific patterns shows donor-specific transcriptomic signatures are highest in the parental fibroblasts of the corresponding donor. (**B**) Scatter plots showing the association between genetic similarity and the strength of donor-specific transcriptomic signatures across pair-wise comparisons with other donors. Each point is a comparison of the highlighted donor to another donor. Genetic distance between donors was quantified as the number of differing alleles across all genotyped single nucleotide polymorphisms (SNPs on the X-axis). The transcriptional distance was quantified as the strength of the donor-specific transcriptomic signature in other donors' tissue (Y-axis; same data depicted in the Y-axis of Figures 5Aii and 5Cii, centered such that the source donor has a value of zero). Pearson's correlation and p-values were calculated by omitting the donor data point at the origin. Solid black lines pass through the origin and each ethnicity's average Y-value, indicated as horizontal dashed lines. The ethnicity of donors is indicated by color: CAUC, Caucasian; AA, African American; HISP, Hispanic; AS, Asian. (**C**) Contribution of genes of 5 evolutionary eras (1, Ancient; 2, Animal; 3, Chordate; 4, Mammal; 5, Primate) to the conserved or divergent dynamic patterns of GWCoGAPS-II analysis. Dynamic patterns conserved across cell lines show high gene amplitudes in ancient genes, while dynamic patterns divergent across cell lines show higher gene amplitudes in newer genes. Comparison of the distribution of era 1 gene amplitudes to era 5 gene amplitudes by Wilcoxon rank sum test: $p>1e-16$ for all patterns shown. (**D**) Contribution of genes of 5 evolutionary eras to the conserved or divergent dynamic and cell line-specific patterns of GWCoGAPS-I analysis ($p<1e-16$ for all patterns shown). (**E**) Projection of RNA-seq data into the neural patterns P15 and P12. (**F**) Differential SOX21 levels in SA01, i04, and two 2053 lines. *, Comparison between 2053-2 and 2053-6 ($p<0.05$). n=3 independent experiments. (**G**) Two replicate lines from donor 2053 show differential responsiveness to RA. Number of HOXB1 expressing cells on day 8 in response to varying doses of RA in SA01, i04, and two 2053 lines. *, Comparison between 2053-2 and 2053-6 ($p<0.05$). n=3 independent experiments. (**H**) The Venn diagrams showing the copy number variants (CNVs) gained and lost in the two replicate hiPSC lines and the mature post-mortem brain tissue of donors 2053 and 2075 with respect to the reference genome. In all cases, the vast majority of differences from the reference genome are the same across both hiPSC lines and the brain tissues derived from each donor (B2053 and B2075), indicating little change in genomic DNA. A similar analysis for single nucleotide variants (SNVs) showed similar results (data not shown). (**I**) Differential expression of the clustered ZNF genes across the discordant hiPSC lines 2053-2 and 2053-6 (left) and across the 6 hPSC lines (right). Notably, differential expression of KRAB-ZNF genes was also observed between lines derived from the same donor, while genome sequencing revealed no detectable CNVs in these genes. Plots show the expression of each gene from its minimum to maximum in pluripotency, NSB/LSB, and BMP4 conditions for all lines. (**J**) Expression of *ZNF208* and *ZNF729*, two of the genes expressed specifically in line 2053-6 (Figure S6I) in the NextGen hiPSC data. Replicate lines from individual donors are displayed as points along a single X-axis value. Donors are ordered by their rank in the first principal component of this data and colored by RA-responsive gene expression. *ZNF208* shows high expression in most lines, with a minority of lines showing very low expression. In contrast, *ZNF729* showed no reads in most lines, and a few lines showed significant expression. While quite distinct from one another, the expression patterns of both genes are consistent with epigenetic regulation, i.e., divergent expression levels across multiple lines derived from the same donor. (**K**) Projection of H3K4me3 and H3K27me3 ChIP-seq data from lines 2053-2 and 2053-6 in SR into the 2053-6 line-specific pattern. Ranges of the Y axis of ChIP-seq projections represent the biggest variation observed within all the patterns for each histone modification as we have done with H3K9me3-projection in Figure 5E. (**L**) H3K4me3 and H3K27me3 ChIP-seq

data at the GBX2 locus in lines 2053-2 and 2053-6 in SR condition.

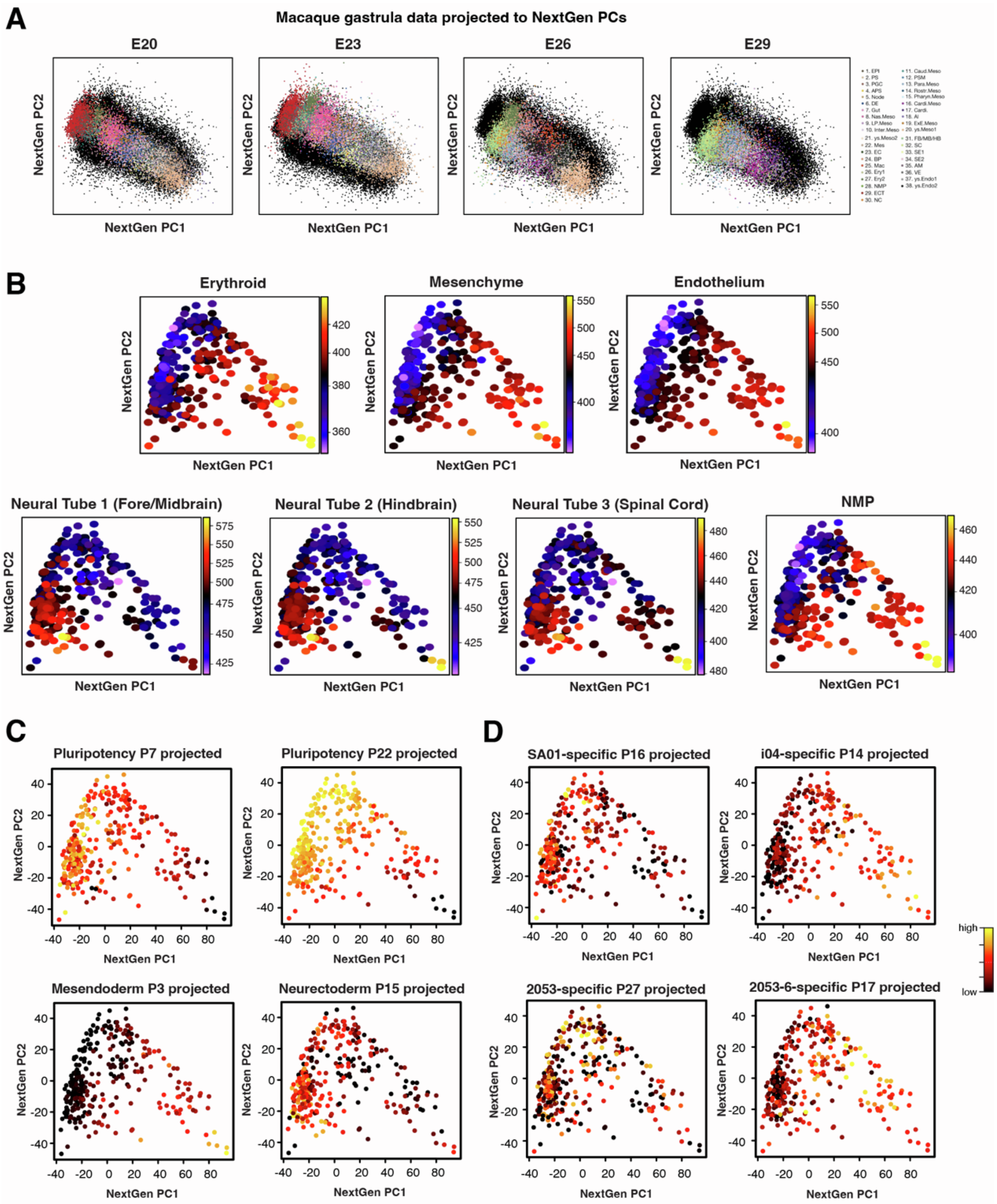

Figure S6. Early developmental bias and retinoic acid signaling define hPSC variation in the wider human population, related to Figure 6. (**A**) Projection of Carnegie stage CS8-11 cynomolgus macaque gastrula data (Zhai et al., 2022) into NextGen PC1 and PC2. (**B**) Average expression of lineage-specific gene sets defined by the transcriptomic signature of CS10 human embryo (Zeng et al., 2023) in the NextGen PSC lines. (**C**) Projection of GWCoGAPs-I dynamic patterns into PC1 and PC2 of NextGen hPSC RNA-seq data(Carcamo-Orive et al., 2017). (**D**) Projection of GWCoGAPs-I and -II cell line-specific patterns into PC1 and PC2 of NextGen hPSC RNA-seq data

Supplemental Table Legends (separate files)

Supplemental Table S1. Information on hPSC lines, RNA-seq data, and individual gene amplitudes for GWCoGAPS-I patterns, related to Figure 2.
Sheet 1: information on hPSC lines used, Sheet 2: summary of experimental times and conditions for RNA-seq data generation, Sheet 3: mapping and annotation details of RNA-seq, Sheet 4: individual gene amplitudes for GWCoGAPS-I patterns, Sheet 5: list of top 100 genes in 22 GWCoGAPS-I patterns,

Supplemental Table S2. Individual gene amplitudes for GWCoGAPS-II and genes ranked by their contribution to the line- and donor-specific patterns, related to Figure 5.
Submitting the top 250 genes from the line- and donor-specific patterns to the Enrichr webtool (https://maayanlab.cloud/Enrichr/enrich?dataset=3f14ebb0f1889f3a5acb6d940326bebb; https://maayanlab.cloud/Enrichr/enrich?dataset=cb12b784682d4aed17f5b17d6cdbee3b) revealed enrichment in 1) Co-expression with many KRAB-ZNF genes (p=7.8e-9 to p=5.8e-6) from the ARCHS4 database, 2) H3K9me3 (p=1.8e-19 to p=6.4e-7) and SETDB1 (p=9.7e-8) ChIP-seq peaks from the ENCODE Histone modifications and TF ChIP-seq databases, and 3) TRIM28/KAP1 protein-protein interactions from the BioPlex database (p=6.8e-6). These enrichments represent a canonical mechanism of transcriptional repression: KRAB-ZNF genes bind repetitive genomic DNA derived from TEs and TRIM28/KAP1, recruiting SETDB1 to deposit the H3K9me3. We also found enrichment of protocadherin (PCDH) genes among the GWCoGAPS gene amplitudes in line-and donor-specific patterns (p=2e-17 and p=0.00046, in GWCoGAPS-I and –II, respectively). Y chromosome genes in these lists serve as a control positive in that their expression patterns are described primarily as a combination of the male line-specific patterns. Genes in line- and donor-specific expression patterns can be explored in our NeMO Analytics portal, where examples of both epigenetic (e.g., ZNF genes in Figures S5I and S5J) and genetic (e.g., NOMO3) regulation can be observed in our hPSC lines and the NextGen Consortium lines: https://nemoanalytics.org/p?l=Kim2024&g=NOMO3.

Supplemental Table S3. **Normalized H3K9me3 levels in 2053-2 and 2053-6 lines in self-renewal**, related to Figure 5. In particular, *GBX2* and other hindbrain fate genes IRX1/2/4, ZIC1, and OLIG3 were highly represented in the 2053-6 line-specific signature and showed higher H3K9me3 levels in 2053-2. Although HOX genes were not expressed in SR condition, 28 HOX genes were among the top 100 with increased H3K9me3 levels in line 2053-2.

Supplemental Table S4. Gene sets used to explore pluripotency states and anterior-to-posterior bias in NextGen hiPSC library dataset, related to Figure 6.
Sheet 1: Gene weights of PC3 of mouse mid-streak embryo data. Sheet 2: Values of retinoic acid response genes in PC1 and PC2 of NextGen RNA-seq data. Sheet 3: Gene weights of PC1 of mouse ESC and EpiSC data.

Supplemental experimental procedures

hPSC culture and differentiation

For posterior neural differentiation, cells were cultured with Aggrewell medium (05893, Stem Cell Technology) for 2 days after ROCK inhibitor removal and then cultured with N2B27 medium supplemented with LDN193189 (100 nM, 04-0074, Stemgent) and SB431542 (2 μM, S4317, Sigma-Aldrich) for another 4 days. Retinoic acid (RA, R2625, Sigma-Aldrich) was added on day 4 to induce posterior neuroectoderm differentiation. For further differentiation to hindbrain-derived neurons, cells were cultured with neurobasal medium (21103-049, Life Technologies) supplemented with bovine Insulin (25 μg/ml, I6634, Sigma-Aldrich), B27 (17504-044, Life Technologies), human BDNF (10 ng/ml, 248-BD, R&D Systems) and human NT-3 (10 ng/ml, 267-N3, R&D Systems) for another 20 days. For inhibition of RA signaling, selective RARα antagonist BMS195614 (5 μM, 3660, Tocris) and RARβ antagonist LE135 (5 μM, 2021, Tocris) were additionally added to the neuroectoderm condition (LDN193189, 100 nM and SB431542, 2 μM) for 4 days.

Generation of hiPSC lines

Previously reported hiPSC lines i04, i07, and i13 (NIH-i4, NIH-i7, NIH-i13) are detailed in(Mallon *et al.*, 2013). The hiPSC lines (2075, 2053, and 2063) were reprogrammed using synthetic mRNAs (Oct4, Klf4, Sox2, c-Myc, Lin-28; OSKML), mRNA reprogramming kit (00-0071, Stemgent), and microRNA Booster kit (00-0073, Stemgent). Human fibroblasts (Donor 2075, 2053, and 2063) were seeded at 5 X 10^3 cells/cm^2 in a Matrigel-coated plate and cultured with DMEM medium supplemented with 10% FBS (Life Technologies) and 2 mM L-glutamine. After 24 hours (day 1), the medium was changed to Pluriton human NUFF-conditioned media with 300 ng/ml B18R protein. On days 1 and 5, the microRNA booster kit was used with the StemFect RNA transfection reagent kit (00-0069, Stemgent) to enhance reprogramming. On days 2-12, the OSKML RNAs were transfected. The mRNA reprogramming process was performed at 37°C in a 5% O_2 and CO_2 incubator. These lines were validated for pluripotency marker expression, trilineage differentiation potential, and normal karyotype, with karyotyping performed every 10 passages.

Generation of CRISPR/Cas9 mediated SOX21-KO hESC line

SOX21-KO hESC lines were generated using CRISPR/Cas9. SOX21-specific gRNAs were designed using the Optimized CRISPR Design-MIT for Sox21NHEJ4 (http://crispr.mit.edu/) and CHOPCHOP for Sox21NHEJ5 (https://chopchop.rc.fas.harvard.edu/). Oligonucleotides (CACCGCGGGCTCAGCGGCGCAAGA –top for Sox21NHEJ4; AAACTCTTGCGCCGCTGAGCCCGC –bottom for Sox21NHEJ4; CACCGGGTGTGGTCGCGGGCTCAG –top for Sox21NHEJ5; AAACCTGAGCCCGCGACCACACCC –bottom for Sox21NHEJ5) were cloned into pSpCas9(BB)-2A-Puro (px459; Addgene), producing plasmid pX459-Sox21NHEJ4 and pX459-Sox21NHEJ5, synthesized by Integrated DNA Technologies. SA01 hESCs were transfected with 2.5 μg of either plasmid using DNA-In Stem (MTI-Global stem, gifted from Dr. Jessee). Transfected cells were dissociated and plated into a 10 cm culture dish. After 48 hours of 0.5 μg/ml puromycin selection, hESC colonies were maintained for 10 days. Individual colonies were isolated and clonally expanded. Genomic DNA was isolated from each clonal line using Wizard Genomic DNA Purification Kit (Promega). The genomic region surrounding the CRISPR target site for SOX21 was amplified by PCR (KOD Xtreme Hot Start DNA Polymerase; EMD Millipore), and products were treated with SURVEYOR nuclease (SURVEYOR Mutation Detection Kit, Transgenomic) to detect CRISPR/Cas9-induced indel mutations. The PCR products were cloned

into pGEM®-T Easy Vector (Promega) and sequenced to confirm the genotypes. Knockout validation of SOX21 protein was performed by immunostaining.

Immunofluorescence

Cells were fixed with 4% paraformaldehyde for 10 min and permeabilized for 40 min using 0.1% Triton X-100 (Sigma-Aldrich) in PBS. Subsequently, cells were blocked with 10% donkey serum (Sigma-Aldrich) and incubated with primary antibodies overnight. Following primary antibodies and dilutions were used: TBXT (AF2085, R&D, 1:500), CDX2 (AM392, Biogenex), EOMES (ab23345, Abcam, 1:400), GATA3 (MAB6330, R&D, 1:200), GATA4 (AF2606, R&D, 1:400), GBX2 (AF4638, R&D, 1:200), HOXB1 (AF6318, R&D, 1:200), HOXB4 (ab133621, Abcam, 1:400), HOXB9 (ab66765, Abcam, 1:400), ID1 (AF4377, R&D, 1:200), ISLET1 (AF1837, R&D, 1:200), NANOG (AF1997, R&D, 1:200; Reprocell 1:200), OCT4A (MAB17591, R&D, 1:200), OLIG2 (AF2418, R&D, 1:200), OTX2 (AF1979, R&D, 1:200), PAX6 (PRB-278P, BioLegend, 1:500; AF8150, R&D, 1:200), PHOX2B (AF4940, R&D, 1:200), p-SMAD1/5 (9516, Cell Signaling Technology, 1:200), p-SMAD2/3 (8828, Cell Signaling Technology, 1:200), and TUJ1 (PRB-435P, BioLegend, 1:1000), SOX1 (AF3369, R&D, 1:400), SOX17 (AF1924, R&D, 1:500), SOX2 (AF2018, MAB2018, R&D, 1:200), SOX21 (AF3538, R&D, 1:200), SOX3 (GT15119, Neuromics, 1:200), and TUJ1 (MAB1195, R&D, 1:400). Secondary antibody incubation was performed with Alexa flour conjugated antibodies at dilution of 1:400 (Life Technologies). For direct immunostaining, primary antibodies were conjugated using Alexa fluor monoclonal antibody labeling kits (A20181, A20184, A20186, Life Technologies). Nuclei were counterstained with DAPI (Life Technologies).

High-content spatial analysis

Images were acquired with the Operetta (Perkin Elmer), analyzed in batch mode with custom building blocks on a Columbus server (Perkin Elmer), and visualized with Spotfire (Perkin Elmer). Spatial analysis of hPSC epithelium (‘distance from the edge’ measurement) was achieved using a custom Acapella script (Perkin Elmer) run in Columbus with the following commands; 1) stitch a montage from 3x3 user-defined contiguous overlapping fields captured with the 20x objective, 2) segment and binarize DAPI signal from individual nuclei to create nuclear objects, 3) segment and binarize DAPI signal from the cytoplasm surrounding each nucleus to create cytoplasmic objects. hPSCs show strong blue fluorescence arising from sequestration of retinyl esters in cytoplasmic lipid bodies(Muthusamy et al., 2014), 4) dilate nuclear objects to eliminate gaps between neighboring objects, 5) create super objects by filling holes containing less than 30 pixels, 6) segment super objects, 7) create a perimeter line at the edge of each super object, 8) calculate the minimum distance between the centroid of each nucleus and the closest super object perimeter, 9) report fluorescence signal from nucleus and cytoplasm for each object. For each cell, this script reports nuclear and cytoplasmic signals for all channels and a single measure of minimum distance to the closest perimeter of the epithelium. Using data visualization in Spotfire, median fluorescence signals from all cells were plotted corresponding to the distance from an edge of epithelium.

RNA-seq library preparation

Total RNA was extracted using the mirVana kit (Ambion). RNA quality control was performed using the Agilent 2100 Bioanalyzer System. RNA-seq libraries were constructed using Illumina mRNA sequencing sample Prep Kit (for Poly-A libraries) or TruSeq Stranded Total RNA RiboZero sample Prep Kit (for strand-specific libraries). Briefly, poly-A-containing mRNA molecules were purified or ribosomal RNAs were removed using RiboZero beads from ~ 800 ng DNase-treated total RNA. Following purification, the resulting RNA was fragmented into small pieces using

divalent cations under elevated temperature at 94°C for 2 min. The range of the fragment length was 130-290 bp, with a median length of 185 bp. Reverse transcriptase and random primers were used to copy the cleaved RNA fragments into first-strand cDNA. The second-strand cDNA was synthesized using DNA Polymerase I and RNase H. These cDNA fragments went through an end repair process using T4 DNA polymerase, T4 PNK, and Klenow DNA polymerase, the addition of a single 'A' base using Klenow exo (3' to 5' exo minus) and the ligation of Illumina PE adapters using T4 DNA Ligase. An index was inserted into Illumina adapters so that multiple samples could be sequenced in one lane of an 8-lane flow cell if necessary. The concentration of RNA was measured by Qubit (Life Technologies). The quality of the RNA-seq library was measured by LabChipGX (Caliper) using HT DNA 1K/12K/HiSens Labchip. The final cDNA libraries were sequenced using HiSeq 2000 (for Poly-A library preparation) or HiSeq 3000 (for RiboZero library preparation) for high-throughput DNA sequencing.

RNA-seq data processing

After the sequencing, the Illumina Real Time Analysis (RTA) module was used to perform image analysis and base calling, and the BCL Converter (CASAVA v1.8.2) was used to generate FASTQ files, which contain the sequence reads. The sequencing depth was over 80 million (40 million paired-end) mappable sequencing reads (Table S1). Read-level Q/C was performed by FastQC (v0.10.1). Pair-end reads of cDNA sequences were aligned back to the human genome (UCSC hg19 from Illumina iGenome) by the spliced read mapper TopHat (v2.0.4) with default option with "--mate-innder-dist 160" based on known transcripts of Ensembl Build GRCh37.75. For stranded RiboZero samples, TopHat used "--library-type fr-firststrand" option. The alignment statistics and Q/C were achieved by samtools (v0.1.18) and RSeQC (v2.3.5) to calculate quality control metrics on the resulting aligned reads, which provides useful information on mappability, uniformity of gene body coverage, insert length distributions and junction annotation, respectively. To achieve a gene-level expression profile, the properly paired and mapped reads were achieved by "samtools sort –n" option, and these reads were counted by htseq-count v0.5.3 (with intersection-strict mode and stranded option for RiboZero samples) according to gene annotation (Illumina iGenome), and RPKM was calculated. This provided 23,368 gene-level expression profiles.

Statistics for SOX21 protein level

To determine the effect of a cell line of origin on nuclear SOX21 protein levels in Figure 1B, we used a mixed model comparing the mean expression levels across cell lines while accounting for the correlation of expression levels within replicate experiments (a total of 5 independent experiments were conducted) with a random intercept, implemented in R using the lme4 library and the lmer() function: expression~ as.factor (line)*condition*day+(1| replicate). To test the effect of the line of origin, this model was compared to a second model with no line effect using anova().

Bioinformatic analyses

Principle component analysis was done using the prcomp() function in R. Agglomerative hierarchical clustering of genes using gene-level RPKM from RNA-seq data was performed using hclust() and cutree() with correlational distance (dist=1-r) in R. GWCoGAPS was run using default parameters as previously described(Fertig et al., 2012; Fertig et al., 2014; Fertig and Favorov, 2010; Stein-O'Brien et al., 2017), for a range of k patterns (k=22 selected) and uncertainty as 10% of the data. Briefly, whole transcriptomic data was parallelized into seven sets. GWCoGAPS decomposes a matrix of experimental observations, **D**—here, log2 RNA-seq RPKMs—with genes as rows and samples as columns, into two matrices, by the following equation.

$$\mathbf{D} \sim N(\mathbf{AP},\Sigma)$$

Where, **A** is the amplitude matrix indicating the strength of involvement of a given gene in each pattern, and **P** is the pattern matrix defining relationships (i.e., patterns) between samples. N and Σ are functions of each element of **AP** and represent the Normal distribution and the standard deviation, respectively. Projection of principal components and GWCoGAPS gene weights defines patterns of relationships between samples in new data associated with the gene expression signatures of the patterns from the primary data. These were achieved using the default projectR function in the projectR package as previously described(Fertig *et al.*, 2014). Enrichment was calculated via either the calcCoGAPSStat function in the CoGAPS Bioconductor package or the geneSetTest function in the limma Bioconductor package in R. ANOVAs were used to assess the association of each GWCoGAPS pattern with treatment, time, and cell line of origin, using lm() and summary() in R: lm(pattern~treatment*day+line). No filtering was applied for the NMF decomposition. All genes in the dataset were used in the GWCoGAPS analysis. This choice was made for multiple reasons. First, the RNA-seq data are from bulk in vitro cell samples that yielded ample high-quality starting RNAs sequenced at >79 M reads per sample (mean=127 M). The signal-to-noise ratio of this deeply sequenced bulk data is very high, especially compared to the currently popular scRNA-seq, which often requires heavy filtering of lowly expressed genes. Second, the GWCoGAPS NMF algorithm can effectively extract patterns from an extremely broad range of gene expression levels(Fertig *et al.*, 2014; Stein-O'Brien *et al.*, 2017; Stein-O'Brien et al., 2019).

Gene age estimation

Gene age was estimated by phylostratigraphy that uses protein sequence similarity scored by BLASTP to find the minimal evolutionary age of protein-coding genes(Domazet-Loso et al., 2007; Weber et al., 2020). For each protein, the National Center for Biotechnology Information (NCBI) nonredundant database was used to find the most distant species in which a sufficiently similar protein sequence exists. We estimated the minimal evolutionary age of a gene as the age of the ancestor of the query species, human in this study, and the most distant species harboring a sufficiently similar sequence. To find the most distant species, we used the NCBI taxonomy for every species and estimated the timing of lineage divergence events with TimeTree(Kumar et al., 2017). As in other studies, we used the e-value threshold of 10^{-3} to detect sequence similarity by BLASTP(Neme and Tautz, 2016; Vakirlis et al., 2020; Weber *et al.*, 2020). For all human protein sequences, we filtered the sequences for a minimal length of 40 amino acids and a maximal length of 4,000 amino acids and kept only one protein isoform per gene (the longest and evolutionary oldest). We counted the number of genes in each phylostratum (PS), from the most ancient (PS 1, Cellular organisms) to the most recent (PS 31, *Homo sapiens*). We aggregated gene counts from individual phylostrata into five broad evolutionary eras: Ancient (PS 1 to 3, Cellular organisms to Opisthokonta; 4290 to 2101 millions of years ago (MYA)); Animal (PS 4 to 7, Metazoa to Deuterostomia, 2101 to 747 MYA); Chordate (PS 8 to 17, Chordate to Amniota, 747 to 320 MYA); Mammal (PS 18 to 22, Mammalia to Euarchontoglires, 320 to 91 MYA); Primate (PS 23 to 31, Primates to *Homo sapiens*, 91 Mya to present).

ChIP-seq

The cells were crosslinked in 1% formaldehyde for 10 min at room temperature with constant agitation, followed by quenching with 125 mM glycine for 5 min. Nuclei were collected on days 2, 4, and 6 in self-renewal. Chromatin was fragmented with micrococcal nuclease (MNase) until the majority of DNA was in the range of 200-700 base pairs. Chromatin was incubated with an H3K9me3 antibody (13969, Cell signaling) at 4°C overnight, with constant agitation. Antibody-bound chromatin was immunoprecipitated with Protein G Dynabeads for 1 hour at 4°C, with

constant agitation. Protein-DNA complexes were eluted from Dynabeads, and the DNA was reverse crosslinked with 0.3 M NaCl with 4 h incubation at 65°C. Free DNA was purified with QIAquick Gel Extraction Kit (28706X4, Qiagen). ChIP-seq libraries were generated using TruSeq ChIP Library Preparation Kit (Illumina, IP-202-1012) and the libraries were sequenced on Illumina HiSeq 3000. ChIP-seq raw reads were aligned to the human genome assembly hg19 using Burrows-Wheeler Aligner (BWA), and peak calling was done using MACS2.1.1. To understand H3K9me3 occupancy in promotors, ChIP-seq reads within +/-3kb of TSS were counted using bedtools.

Supplemental References

Burke, E.E., Chenoweth, J.G., Shin, J.H., Collado-Torres, L., Kim, S.K., Micali, N., Wang, Y., Colantuoni, C., Straub, R.E., Hoeppner, D.J., et al. (2020). Dissecting transcriptomic signatures of neuronal differentiation and maturation using iPSCs. Nature communications *11*, 462. 10.1038/s41467-019-14266-z.

Carcamo-Orive, I., Hoffman, G.E., Cundiff, P., Beckmann, N.D., D'Souza, S.L., Knowles, J.W., Patel, A., Papatsenko, D., Abbasi, F., Reaven, G.M., et al. (2017). Analysis of Transcriptional Variability in a Large Human iPSC Library Reveals Genetic and Non-genetic Determinants of Heterogeneity. Cell stem cell *20*, 518-532.e519. 10.1016/j.stem.2016.11.005.

Choi, J., Lee, S., Mallard, W., Clement, K., Tagliazucchi, G.M., Lim, H., Choi, I.Y., Ferrari, F., Tsankov, A.M., Pop, R., et al. (2015). A comparison of genetically matched cell lines reveals the equivalence of human iPSCs and ESCs. Nature biotechnology *33*, 1173-1181. 10.1038/nbt.3388.

Domazet-Loso, T., Brajkovic, J., and Tautz, D. (2007). A phylostratigraphy approach to uncover the genomic history of major adaptations in metazoan lineages. Trends in genetics : TIG *23*, 533-539. 10.1016/j.tig.2007.08.014.

Fertig, E.J., Ren, Q., Cheng, H., Hatakeyama, H., Dicker, A.P., Rodeck, U., Considine, M., Ochs, M.C., and Chung, C.H. (2012). Gene expression signatures modulated by epidermal growth factor receptor activation and their relationship to cetuximab resistance in head and neck squamous cell carcinoma. BMC genomics *13*.

Fertig, E.J., Stein-O'Brien, G., Jaffe, A., and Colantuoni, C. (2014). Pattern identification in time-course gene expression data with the CoGAPS matrix factorization. Methods Mol Biol *1101*, 87-112. 10.1007/978-1-62703-721-1_6.

Fertig, E.J.D., J., and Favorov, A.V.P., G. Ochs, M. F. (2010). CoGAPS: an R/C++ package to identify patterns and biological process activity in transcriptomic data. Bioinformatics *26*, 2792-2793. 10.1093/bioinformatics/btq503.

Kumar, S., Stecher, G., Suleski, M., and Hedges, S.B. (2017). TimeTree: A Resource for Timelines, Timetrees, and Divergence Times. Mol Biol Evol *34*, 1812-1819. 10.1093/molbev/msx116.

Kyttala, A., Moraghebi, R., Valensisi, C., Kettunen, J., Andrus, C., Pasumarthy, K.K., Nakanishi, M., Nishimura, K., Ohtaka, M., Weltner, J., et al. (2016). Genetic Variability Overrides the Impact

of Parental Cell Type and Determines iPSC Differentiation Potential. Stem Cell Reports *6*, 200-212. 10.1016/j.stemcr.2015.12.009.

Mallon, B.S., Chenoweth, J.G., Johnson, K.R., Hamilton, R.S., Tesar, P.J., Yavatkar, A.S., Tyson, L.J., Park, K., Chen, K.G., Fann, Y.C., and McKay, R.D. (2013). StemCellDB: the human pluripotent stem cell database at the National Institutes of Health. Stem cell research *10*, 57-66. 10.1016/j.scr.2012.09.002.

Muthusamy, T., Mukherjee, O., Menon, R., Megha, P.B., and Panicker, M.M. (2014). A method to identify and isolate pluripotent human stem cells and mouse epiblast stem cells using lipid body-associated retinyl ester fluorescence. Stem Cell Reports *3*, 169-184. 10.1016/j.stemcr.2014.05.004.

Neme, R., and Tautz, D. (2016). Fast turnover of genome transcription across evolutionary time exposes entire non-coding DNA to de novo gene emergence. eLife *5*, e09977. 10.7554/eLife.09977.

Pijuan-Sala, B., Griffiths, J.A., Guibentif, C., Hiscock, T.W., Jawaid, W., Calero-Nieto, F.J., Mulas, C., Ibarra-Soria, X., Tyser, R.C.V., Ho, D.L.L., et al. (2019). A single-cell molecular map of mouse gastrulation and early organogenesis. Nature *566*, 490-495. 10.1038/s41586-019-0933-9.

Stein-O'Brien, G.L., Carey, J.L., Lee, W.S., Considine, M., Favorov, A.V., Flam, E., Guo, T., Li, S., Marchionni, L., Sherman, T., et al. (2017). PatternMarkers & GWCoGAPS for novel data-driven biomarkers via whole transcriptome NMF. Bioinformatics, 1-3. 10.1093/bioinformatics/btx058.

Stein-O'Brien, G.L., Clark, B.S., Sherman, T., Zibetti, C., Hu, Q., Sealfon, R., Liu, S., Qian, J., Colantuoni, C., Blackshaw, S., et al. (2019). Decomposing Cell Identity for Transfer Learning across Cellular Measurements, Platforms, Tissues, and Species. Cell Syst *8*, 395-411 e398. 10.1016/j.cels.2019.04.004.

Vakirlis, N., Carvunis, A.R., and McLysaght, A. (2020). Synteny-based analyses indicate that sequence divergence is not the main source of orphan genes. eLife *9*. 10.7554/eLife.53500.

Weber, J.A., Park, S.G., Luria, V., Jeon, S., Kim, H.M., Jeon, Y., Bhak, Y., Jun, J.H., Kim, S.W., Hong, W.H., et al. (2020). The whale shark genome reveals how genomic and physiological properties scale with body size. Proceedings of the National Academy of Sciences of the United States of America *117*, 20662-20671. 10.1073/pnas.1922576117.

Zhai, J., Guo, J., Wan, H., Qi, L., Liu, L., Xiao, Z., Yan, L., Schmitz, D.A., Xu, Y., Yu, D., et al. (2022). Primate gastrulation and early organogenesis at single-cell resolution. Nature *612*, 732-738. 10.1038/s41586-022-05526-y.
