# Supplementary material for: Individual variation in the emergence of anterior-to-posterior neural fates from human pluripotent stem cells
Source: Stem Cell Reports. 2024 Aug 15;19(9):1336–50. doi: 10.1016/j.stemcr.2024.07.004 (PMC11411333; doi:10.1016/j.stemcr.2024.07.004)
Supplement: Document S2. Article plus supplemental information [file mmc6.pdf]

Stem Cell Reports
Article
OPEN ACCESS

Individual variation in the emergence of anterior-to-posterior neural fates from human pluripotent stem cells

Suel-Kee Kim,[1,2,16] Seungmae Seo,[1,16] Genevieve Stein-O'Brien,[1,16] Amritha Jaishankar,[1,16] Kazuya Ogawa,[1] Nicola Micali,[1,2] Victor Luria,[2,4,5] Amir Karger,[6] Yanhong Wang,[1] Hyojin Kim,[2] Thomas M. Hyde,[1,8,10] Joel E. Kleinman,[1,8] Ty Voss,[13] Elana J. Fertig,[9] Joo-Heon Shin,[1] Roland Bürli,[14] Alan J. Cross,[14] Nicholas J. Brandon,[14] Daniel R. Weinberger,[1,8,10,11,12] Joshua G. Chenoweth,[1] Daniel J. Hoeppner,[1] Nenad Sestan,[2,3,*] Carlo Colantuoni,[1,8,11,15,*] and Ronald D. McKay[1,7,17,*]
[1]Lieber Institute for Brain Development, 855 North Wolfe Street, Baltimore, MD 21205, USA
[2]Department of Neuroscience, Yale School of Medicine, New Haven, CT 06510, USA
[3]Departments of Genetics, Psychiatry, and Comparative Medicine, Kavli Institute for Neuroscience, Program in Cellular Neuroscience, Neurodegeneration and Repair, Child Study Center, Yale School of Medicine, New Haven, CT 06510, USA
[4]Department of Systems Biology, Harvard Medical School, Boston, MA 02115, USA
[5]Division of Genetics and Genomics, Boston Children's Hospital, Harvard Medical School, Boston, MA 02115, USA
[6]IT-Research Computing, Harvard Medical School, Boston, MA 02115, USA
[7]Departments of Cell Biology, Johns Hopkins School of Medicine, Baltimore, MD 21205, USA
[8]Departments of Neurology, Johns Hopkins School of Medicine, Baltimore, MD 21205, USA
[9]Departments of Oncology, Biomedical Engineering, and Applied Mathematics and Statistics, Johns Hopkins School of Medicine, Baltimore, MD 21205, USA
[10]Departments of Psychiatry, Johns Hopkins School of Medicine, Baltimore, MD 21205, USA
[11]Departments of Neuroscience, Johns Hopkins School of Medicine, Baltimore, MD 21205, USA
[12]McKusick-Nathans Institute of Genetic Medicine, Johns Hopkins School of Medicine, Baltimore, MD 21205, USA
[13]Division of Preclinical Innovation, Nation Center for Advancing Translational Science, NIH, Bethesda, MD 20892, USA
[14]Astra-Zeneca Neuroscience iMED., 141 Portland Street, Cambridge, MA 01239, USA
[15]Institute for Genome Sciences, University of Maryland School of Medicine, Baltimore, MD 21201, USA
[16]These authors contributed equally
[17]Lead contact
*Correspondence: nenad.sestan@yale.edu (N.S.), ccolantu@jhmi.edu (C.C.), ronaldmckay@mac.com (R.D.M.)
https://doi.org/10.1016/j.stemcr.2024.07.004

SUMMARY

Variability between human pluripotent stem cell (hPSC) lines remains a challenge and opportunity in biomedicine. In this study, hPSC lines from multiple donors were differentiated toward neuroectoderm and mesendoderm lineages. We revealed dynamic transcriptomic patterns that delineate the emergence of these lineages, which were conserved across lines, along with individual line-specific transcriptional signatures that were invariant throughout differentiation. These transcriptomic signatures predicted an antagonism between SOX21-driven forebrain fates and retinoic acid-induced hindbrain fates. Replicate lines and paired adult tissue demonstrated the stability of these line-specific transcriptomic traits. We show that this transcriptomic variation in lineage bias had both genetic and epigenetic origins, aligned with the anterior-to-posterior structure of early mammalian development, and was present across a large collection of hPSC lines. These findings contribute to developing systematic analyses of PSCs to define the origin and consequences of variation in the early events orchestrating individual human development.

INTRODUCTION

During mammalian embryonic development, pluripotent epiblast cells undergo spatially constrained cell state transitions to form distinct tissues (Arnold and Robertson, 2009). Human pluripotent stem cells (hPSCs) represent the epiblast state, primed to diversify into the embryonic germ layers, ultimately forming major organs (Bao et al., 2009; Brons et al., 2007; Tesar et al., 2007). Previous studies have examined genomic and transcriptomic variations in hPSC lines (Carcamo-Orive et al., 2017; Choi et al., 2015; Cuomo et al., 2020; DeBoever et al., 2017; Kilpinen et al., 2017; Rouhani et al., 2014). However, we lack a detailed understanding of variation in the transitions from pluripotent cells to neural stem cells with distinct brain regional identities, complicating stem cell applications for neurological and psychiatric disorders. Methods to direct hPSCs to various tissues *in vitro* are advancing, focusing on reproducible cellular output from diverse lines. It is now of great interest to develop assays that further explore the origins of inherent variation in cellular phenotypes among hPSC lines.

Recent studies report developmental differences among hPSC lines in generating regionally specified neural precursors and their possible implications for neurodevelopmental disorders (Jourdon et al., 2023; Kanton et al., 2019; Mariani et al., 2015; Micali et al., 2020; Paulsen et al., 2022; Wang et al., 2020). Here, we employ cellular and genomic approaches to define functional variation in hPSC lines as they progress through neuroectoderm versus

This is an open access article under the CC BY license (http://creativecommons.org/licenses/by/4.0/).

mesendoderm and fore- versus hindbrain development. High-resolution decomposition of gene expression during hPSC differentiation revealed dynamic transcriptomic changes in lineage emergence that were conserved across lines. In addition, these tools defined gene expression specific to individual hPSC lines and donors that remained stable throughout prolonged cell culture and repeated differentiation. These line-specific signatures are regulated by both genetic and epigenetic mechanisms that act through SOX21 and retinoic acid (RA) signaling to control fore- and hindbrain trajectories. Our extensive multi-omics data, combined with relevant public datasets, are accessible at https://nemoanalytics.org/p?l=Kim2024.

RESULTS

Cell line variation in the emergence of neural fate from pluripotency

Previous studies have defined diverse lineage-competent states generating distinct embryonic and extraembryonic fates on unconstrained (Guo et al., 2021; Hough et al., 2014), surface-patterned (Warmflash et al., 2014), and microfluidic-directed (Rifes et al., 2020) hPSC colony organizations. Here, we measured spatial dynamics of early cell fate emergence in unconstrained monolayer culture by monitoring the expression of key lineage regulators and signaling targets. One day after the passage of dissociated single cells (day 0, D0), rho-kinase (ROCK) inhibitor was removed to allow undifferentiated cells to form epithelial sheets. To induce differentiation, hPSCs were exposed to bone morphogenetic protein (BMP)/transforming growth factor β (TGF-β) signaling agonists or antagonists to generate mesendodermal or neural fates, respectively (Chambers et al., 2009; Faial et al., 2015). BMP4 treatment on D0 (D0T) induced phosphorylation of SMAD1/5 and expression of early primitive streak and extraembryonic fate regulators TBXT and CDX2 across the cell population (Figure S1A). When BMP4 was applied on D2 (D2T), the induction of these markers was restricted to the edge of the epithelium (Figures S1A and S1B), while cells in the core remained competent to respond to BMP4, as indicated by SMAD2/3 phosphorylation and expression of SOX17 and GATA4, drivers of primitive and definitive endoderm differentiation (Figure S1B). BMP/TGF-β signaling antagonists Noggin and SB431542 (NSB) induced neural differentiation, with SOX2, SOX21, and OTX2 expressed in the epithelium core. In contrast, edge cells maintained a high level of NANOG (Figures S1C and S1D). These findings indicate that this unconstrained two-dimensional system defines spatial domains in early neuroepithelial lineage emergence.

To measure variation in this spatial organization across multiple hPSC lines, we compared hESC line SA01 with hiPSC line i04 (Mallon et al., 2013). SA01 displayed larger core zones with higher SOX21 and OTX2 expression, while i04 showed larger edge zones with greater NANOG expression (Figures 1A and S1E). These differences persisted under various doses of neuroectodermal inducers and cell-plating densities (Figures S1F and S1G) and were not due to different proliferation rates. In response to BMP4, i04 rapidly induced CDX2, while SA01 predominantly expressed TBXT (Figure S1G). These data suggest that cell line variation in early fate bias can be defined in this system.

Variation in early neural fate was indicated by differential SOX21 expression among six hPSC lines in both self-renewal (SR) and neuroectoderm (NSB) conditions (Figure 1B; Table S1). SA01 line showed the lowest NANOG level and the highest SOX21 induction under neuroectoderm conditions, while line i04 showed the opposite, suggesting an inverse correlation between NANOG and SOX21 expression during neuroectoderm emergence. Principal-component analysis (PCA) of RNA sequencing (RNA-seq) data at 2, 4, and 6 days of SR, NSB, or BMP4 conditions for all lines (Table S1) showed that principal component (PC) 1 corresponded to mesendodermal differentiation and PC2 to temporal changes in differentiation in all conditions (Figure 1C). All cell lines followed similar trajectories along PC1 and PC2, while differences were also evident. SA01 advanced furthest along the NSB trajectory, aligning with SOX21 levels (Figures 1C and S1H). This bias in differentiation was evident in the PCA of NSB samples alone (Figure S1I). Projecting SR data into the NSB dataset revealed that the same ranking of cell lines was present in SR, indicating that the gene expression differences in differentiation were already present in pluripotency (Figure S1I). Projection of SR data into BMP4 PC1 also showed a consistent inverse ranking. These observations suggest that SOX21 expression marks early neuroectodermal specification and that heterogeneity within pluripotency is linked to the emergence of early cell fate bias between lines.

Decomposing dynamic and stable transcriptomic modules in early differentiation

To further dissect this low-dimensional transcriptomic change across cell lines and conditions, we employed the Genome-Wide Coordinated Gene Activity in Pattern Sets (GWCoGAPS) non-negative matrix factorization algorithm (Stein-O'Brien et al., 2017) and identified 22 transcriptomic patterns (GWCoGAPS-I, Table S1). GWCoGAPS patterns decompose multiple signals within individual gene expression. Hence, a combination of patterns represents the complete expression of each gene (Figure S2A). This analysis revealed two classes of patterns (Figure 2A): dynamic

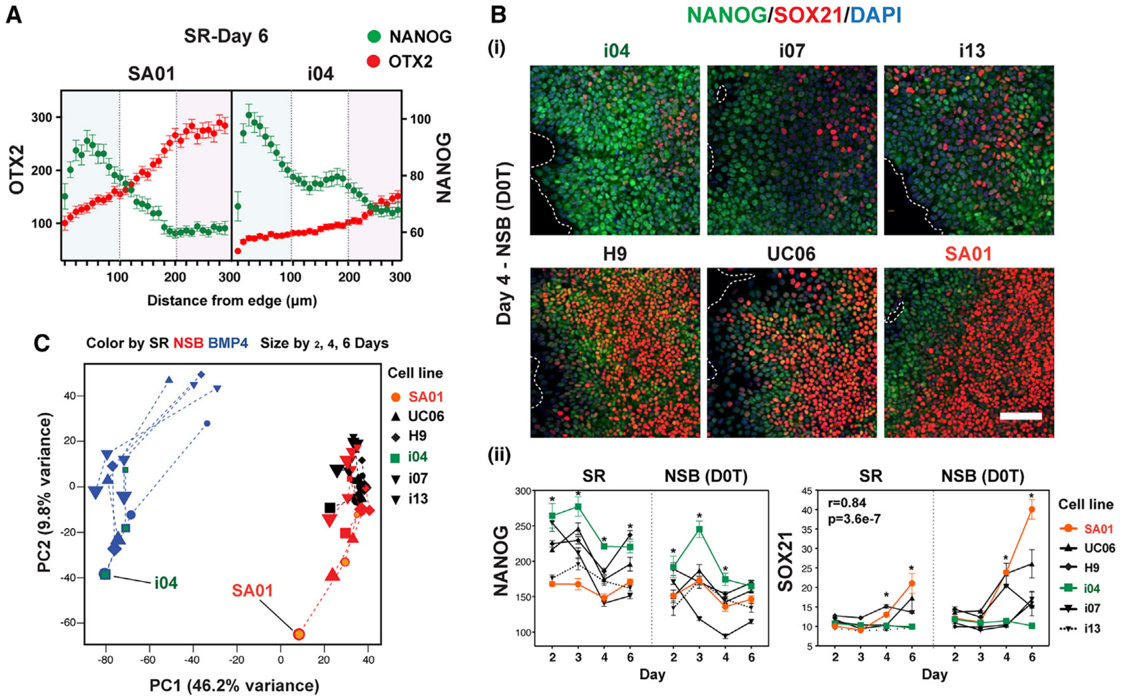

Figure 1. Cell line variation in the emergence of neural fate from pluripotency

(A) Spatial expression on day 6 in SR condition in SA01 and i04 lines.

(B) Variation in NANOG and SOX21 expression across PSC lines. (i) Representative images. Scale bar, 100 µm. (ii) Expression levels in each line across time (n = 5, independent experiments; *, Comparison between SA01 and i04: $p < 0.001$; r and p value refer to the Pearson correlation coefficient between SOX21 levels in SR and NSB).

(C) PCA showing differentiation trajectories.

See also Figure S1.

patterns, which characterized transcriptomic trajectories that changed over time or conditions, and cell line-specific patterns, which defined consistent differences over time and conditions but varied between cell lines.

Among the dynamic patterns (Figure 2A), three patterns, including P7, related to pluripotency; 6 patterns, including P3 and P9, represented a response to BMP4; and three patterns, including P15 and P12, captured different temporal phases of response to NSB. P7 included the core pluripotency genes *POU5F1*, *SOX2*, and *NANOG*. BMP4-induced patterns P3 and P9 included the early mesendodermal and extraembryonic fate regulators *TBXT*, *EOMES*, and GATA family members. NSB patterns P12 and P15 contained the neuroectodermal regulators *OTX2*, *SOX21*, and *PAX6* (Figures S2B; Table S1).

To relate these *in vitro* transcriptomic dynamics with *in vivo* development, we projected single-cell RNA-seq (scRNA-seq) data from developing mouse gastrula (Pijuan-Sala et al., 2019) into the GWCoGAPS-I patterns. The pluripotency module P7 showed the highest levels in epiblast cells and decreased in early germ layer populations (Figures 2B and S2C). In contrast, BMP4 pattern P3 genes increased in mesodermal lineages and posterior primitive streak derivatives. Patterns P15 and P12 genes peaked in neuroectodermal cells (Figures 2C and S2D). Sequential induction of these early neural expression modules was also found in cortical neuron differentiation from multiple hiPSC lines (Burke et al., 2020) (Figure S2Diii).

Genes with high weights in cell line-specific patterns showed higher expression in specific cell lines (Figures 2A, 2Di, and S2E). In general, genes with high weights in dynamic patterns exhibited low weights in cell line-specific patterns (Figure S2E). However, some dynamic pattern genes, such as *OTX2*, *SOX21*, *and ZIC3*, had higher weights in cell line-specific patterns of lines showing more efficient generation of neuroectoderm, while *GBX2* and *FABP7* had higher weights in those of lines more efficient at generating mesendoderm. This suggests that cell line-specific and dynamic transcriptomic signatures interact to influence the early differentiation of cell lines.

Projection of our previous microarray data from the differentiation of the same lines (Mallon et al., 2013) showed that line-specific patterns were stable across vastly different laboratory conditions (Figures 2Dii and S2Fii).

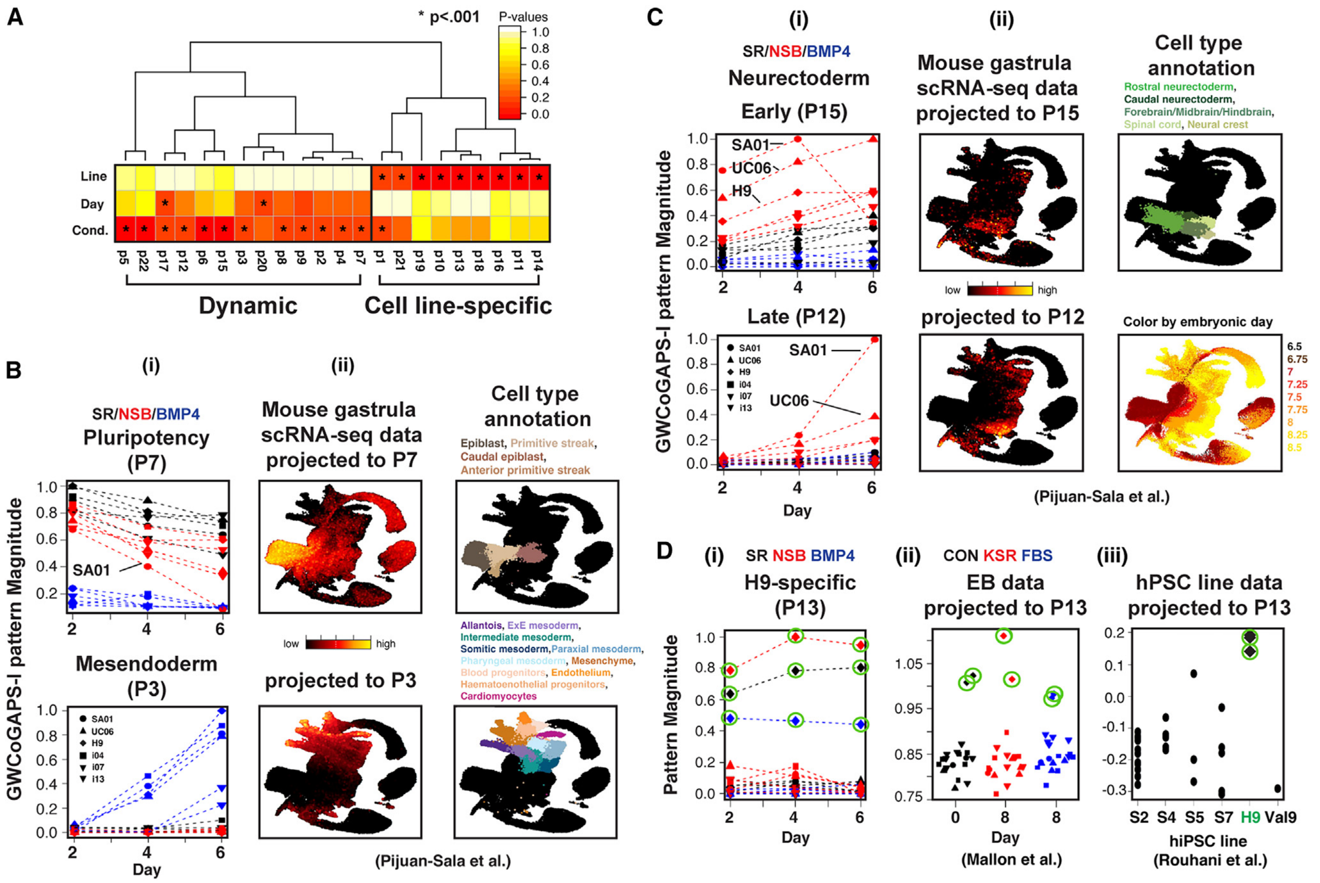

Figure 2. Decomposing dynamic and cell line-specific transcriptomic modules

(A) Hierarchical clustering of GWCoGAPS-I patterns and *p* values from ANOVA analysis of effects of line, day, and condition in each pattern.
(B) (i) GWCoGAPS-I patterns representing loss of pluripotency (P7) or mesendoderm induction (P3). (ii) Projections of mouse gastrula data.
(C) (i) NSB patterns delineating early and later stages of neuroectoderm differentiation. (ii) Projection of mouse gastrula data.
(D) (i) H9-specific transcriptomic signature. (ii) Projection of embryoid body (EB) data from the same lines (Comparison between H9 and other lines, $p = 3.0e-4$). (iii) Projection of multiple hPSC line data. H9 samples are circled in green.
See also Figure S2.

Projection of RNA-seq data from other studies using multiple lines, including H9 (Choi et al., 2015; Kyttala et al., 2016; Rouhani et al., 2014), into the H9-specific pattern showed this line with the strongest signal (Figures 2Diii and S2G). Additionally, projection of DNA methylation data indicated that cell line-specific patterns correlate with hypomethylation at promoters of genes highly expressed in the corresponding cell lines (Figure S2Fiii). These findings suggest that cell line-specific patterns define stable transcriptomic and epigenetic signatures in individual hPSC lines.

SOX21 regulates early forebrain fate by inhibiting mesendoderm and neuromesoderm specification

While the neuroectoderm pattern P15 represents the early transcriptomic changes following NSB treatment, it also exhibited differential responses across lines (Figures 2A and 2C). The high rank of *SOX21* in P15 (Figure S2B) and its induction with OTX2 in NSB condition (Figure S1D) suggest a role in the variation of the cell lines in early forebrain specification. We previously suggested that SOX21 interacts with SOX2 to regulate antero-posterior identity in the adult mouse intestine by repressing CDX2 (Kuzmichev et al., 2012). To define SOX21's role in early neural specification, we generated SOX21-knockout (KO) lines from the SA01 line (Figure S3A). After NSB treatment, SOX21-KO lines showed increased SOX2 and SOX3 expression in the epithelial edge and increased NANOG in both edge and core zones (Figure 3A). Transcriptomic changes in SOX21-KO lines (Figures S3B and S3C) further supported that the loss of SOX21 resulted in sustained pluripotency and delayed transition to neural fates.

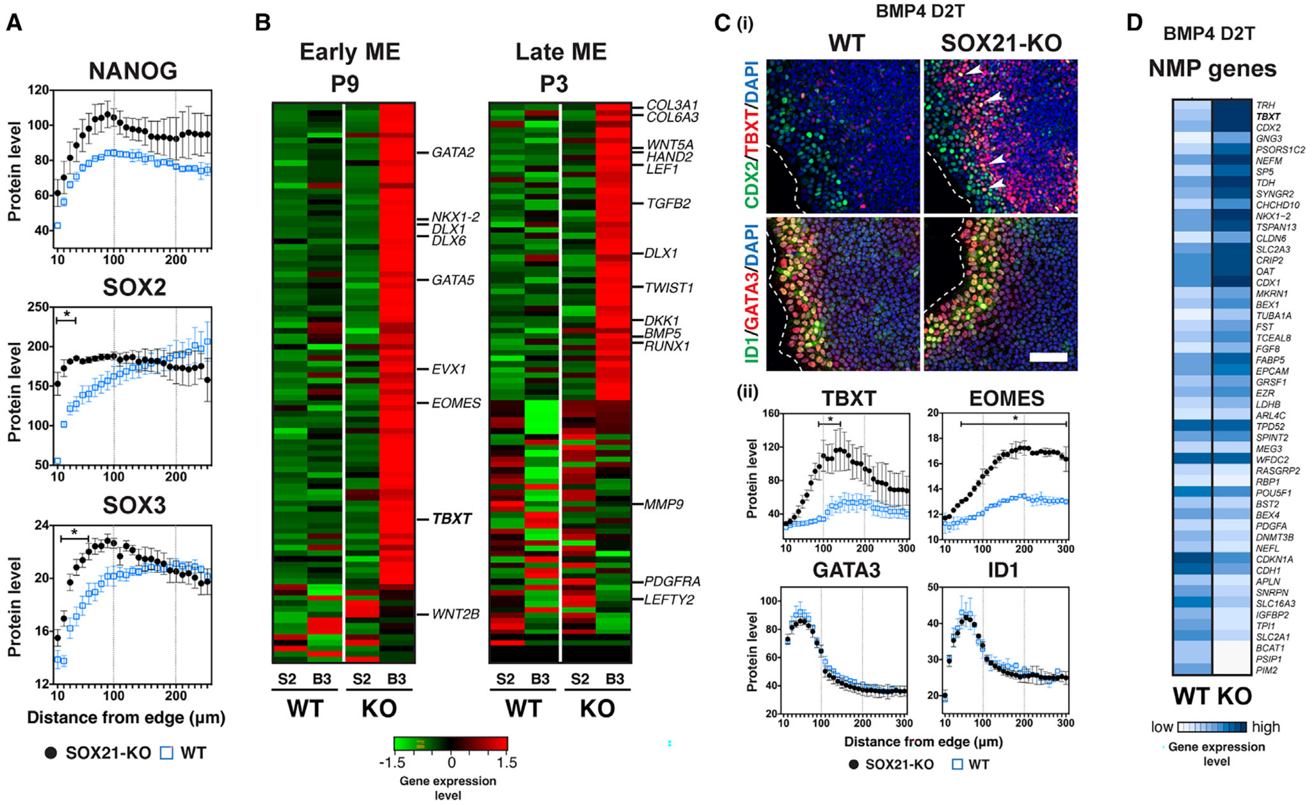

Figure 3. SOX21 mediates early forebrain fate

(A) Spatial expression on D3 in NSB condition.

(B) Expression of the top 100 genes in P9 and P3 on D2 SR (S2) and 24 h after BMP4 treatment on D2 (B3).

(C) (i) Expression of mesendodermal regulators 24 h after BMP4 treatment on D2. Arrowheads indicate coexpression of TBXT and CDX2. Scale bar, 100 µm. (ii) Spatial expression. *, Comparison between WT ($n = 3$, replicate cell lines) and SOX21-KO ($n = 3$): $p < 0.05$.

(D) Expression of NMP genes 24 h after BMP4 D2T.

See also Figure S3.

To test SOX21's relative role in the emergence of mesendodermal and neural fates, SOX21-KO cells were treated with BMP4 on D2T after the core zone had formed. While wild-type (WT) cells showed minimal induction of mesendodermal genes, SOX21-KO cells exhibited strong upregulation of BMP4-responsive genes (Figure 3B). Immunostaining showed no difference in GATA3 and ID1 expression between WT and SOX21-KO lines in the edge zone. However, TBXT and EOMES were significantly induced in the core zone of SOX21-KO cells (Figure 3C). Notably, CDX2 expression extended in the core zone of SOX21-KO cells, resulting in increased TBXT and CDX2 coexpression. In the posterior region of embryos, neuromesodermal precursors (NMPs) generate both spinal cord and trunk mesoderm (Henrique et al., 2015). Consistent with CDX2's role in specifying NMP (Guibentif et al., 2021), the NMP transcriptomic signature and SOX21 expression were mutually exclusive in the mouse gastrula (Figure S3D). Many NMP genes showed higher expression in SOX21-KO cells when treated with BMP4 on D2 (Figure 3D). These results indicate that SOX21 restricts mesendoderm and NMP during the early stages of anterior-to-posterior (A-P) neural fate determination.

SOX21's role in the early specification of rostral and caudal neuroectoderm was supported by the observation that genes upregulated in SOX21-KO cells under NSB condition showed high expression in the epiblast and anterior primitive streak of developing mice (Figure S3Dvi). Interestingly, the restriction of caudal identity was evident from genes upregulated in the SOX21-KO cells under BMP4 D2T condition and in the forebrain of the gastrulating mouse embryo. *TBXT*, which specifies pro-mesoderm and neural crest in the caudal epiblast (Gogolou et al., 2022), was among the targets of SOX21 repression (Figures 3B–3D and S3Dv). Previous studies have reported SOX21's role in promoting extraembryonic fates (Goolam et al., 2016), late forebrain development (Fang et al., 2019), and adult neurogenesis (Matsuda et al., 2012). Our

findings support an early inhibitory role for SOX21 during the transition from pluripotency, restricting posterior neuroectodermal fates and promoting anterior fates in the emerging forebrain.

Cell line-specific transcriptomic signatures underlie variation in forebrain versus hindbrain fate bias

We used multidimensional scaling (MDS) to relate cell line-specific signatures to lineage emergence (Figure 4A). The first MDS dimension distinguished dynamic patterns from cell line-specific signatures. The second dimension separated the NSB patterns from the BMP4 patterns, positioning the SR patterns between the lineage-related patterns. Gene expression changes by SOX21-KO in pluripotency correlated with BMP4 patterns P3 and P8 (Figure 4B), aligning with SOX21's role in repressing mesendoderm specification. In addition, these changes positively correlated with the i04 line-specific signature and negatively with the SA01 line-specific signature. These opposing correlations suggest that these line-specific signatures interact with SOX21, influencing the lineage bias of these lines.

To further examine the differential A-P axis patterning in these lines, we interrogated the expression of genes known to be upregulated by RA signaling (Balmer and Blomhoff, 2002), which plays a role in posterior mesendodermal, neural, and neuromesodermal development (Ghyselinck and Duester, 2019). Consistently, RA-responsive genes were highly expressed in caudal epiblast and posterior neural fates of mouse gastrula (Figure S4A). These genes were significantly enriched in two BMP4 patterns (Figure 4A) and in SOX21-KO cells in BMP4 condition (Figures 4B and S4B). RA exposure reduced SOX21 expression in a dose-dependent manner (Figure S4C). Furthermore, i04 line exhibited the highest expression of RA-responsive genes across all conditions (Figure 4C). Notably, the i04 line-specific signature was significantly enriched with RA-responsive genes (Figure 4D) and aligned with BMP4 patterns in MDS dimension 2 (Figure 4A). In contrast, the SA01 line's signature showed no RA-responsive gene enrichment and aligned with NSB patterns. These results suggest the lower SOX21 expression in i04 line might be related to its high RA signaling activity.

Opposing interactions between OTX2 and GBX2 establish the mid-hindbrain boundary, with the RA-responsive GBX2 expressed in the hindbrain (Millet et al., 1999). These were differentially expressed between SA01 and i04 lines (Figures S4D and S4E), with GBX2 showing higher gene weights in the i04-specific pattern (Figure 4D). Since cell line-specific patterns define stable transcriptomic features, the differential enrichment of RA-responsive genes in these patterns could predict the A-P differentiation efficiency of individual cell lines. To test this hypothesis, we assessed RA dose response in neural differentiation across cell lines and observed that i04, i07, and i13 produced more hindbrain cells than SA01, H9, and UC06 (Figures 4Ei and S4F). RA gene enrichment in the cell line-specific patterns strongly correlated with hindbrain fate potential (Figure 4Eii). Further differentiation showed higher production of hindbrain neurons in i04 (Figure 4F). Thus, cell line-specific signatures associated with RA signaling can predict the differential emergence of anterior versus posterior neuronal fates in hPSC lines.

Next, we modulated RA signaling by applying RA receptor (RAR) inhibitors to the cells. Both *RARA* and *RARB* genes were upregulated during mesendodermal differentiation, with *RARB* gene expression highest in SR condition, particularly in the i04 line (Figure S4G). Inhibiting RARβ in neuroectoderm condition increased SOX21 expression in i04 cells (Figure 4G), suggesting that high RARβ-driven signaling in this line contributes to inefficiency in generating anterior neuroectoderm. We further examined RA response in SA01 line-derived SOX21-KO lines and observed a higher RA response (Figure 4H), indicating that SOX21 can suppress RA-responsive gene expression. These results demonstrate an interplay between SOX21 expression and endogenous RA signaling activity, underlying cell line variation in their response to RA and subsequent A-P identity.

Cell line-specific signatures involve evolutionarily recent genes under genetic control

We next generated six new hiPSC lines, including two replicate lines each from three adult donors whose postmortem brain RNA-seq data were previously generated (Jaffe et al., 2018)(Table S1). The dynamic differentiation trajectories observed in the original hPSC lines (GWCoGAPS-I) were recapitulated in the new analysis (GWCoGAPS-II, Table S2) also identifying transcriptomic signatures that were stable across time and conditions for each donor (Figure 5Ai). Projection of RNA-seq data from the cerebral cortex of 260 individuals into these donor-specific patterns showed that the expression traits were elevated in the corresponding postmortem tissue (Figure 5Aii). Moreover, the donor-specific signatures were also highest in the parental fibroblasts of the same donors (Figure S5A). These expression profiles across replicate lines, fibroblasts, and mature brain tissue suggest that donor-specific signatures comprise stable gene expression traits potentially persisting across an individual's cells throughout their lifespan.

The Genotype-Tissue Expression (GTEx) Project analyzed gene expression and genetic variation across multiple tissues from numerous donors, identifying multi-tissue expression quantitative trait loci (eQTLs) (GTEx Consortium, 2015). Genes with high amplitudes in our donor-specific signatures were significantly enriched in

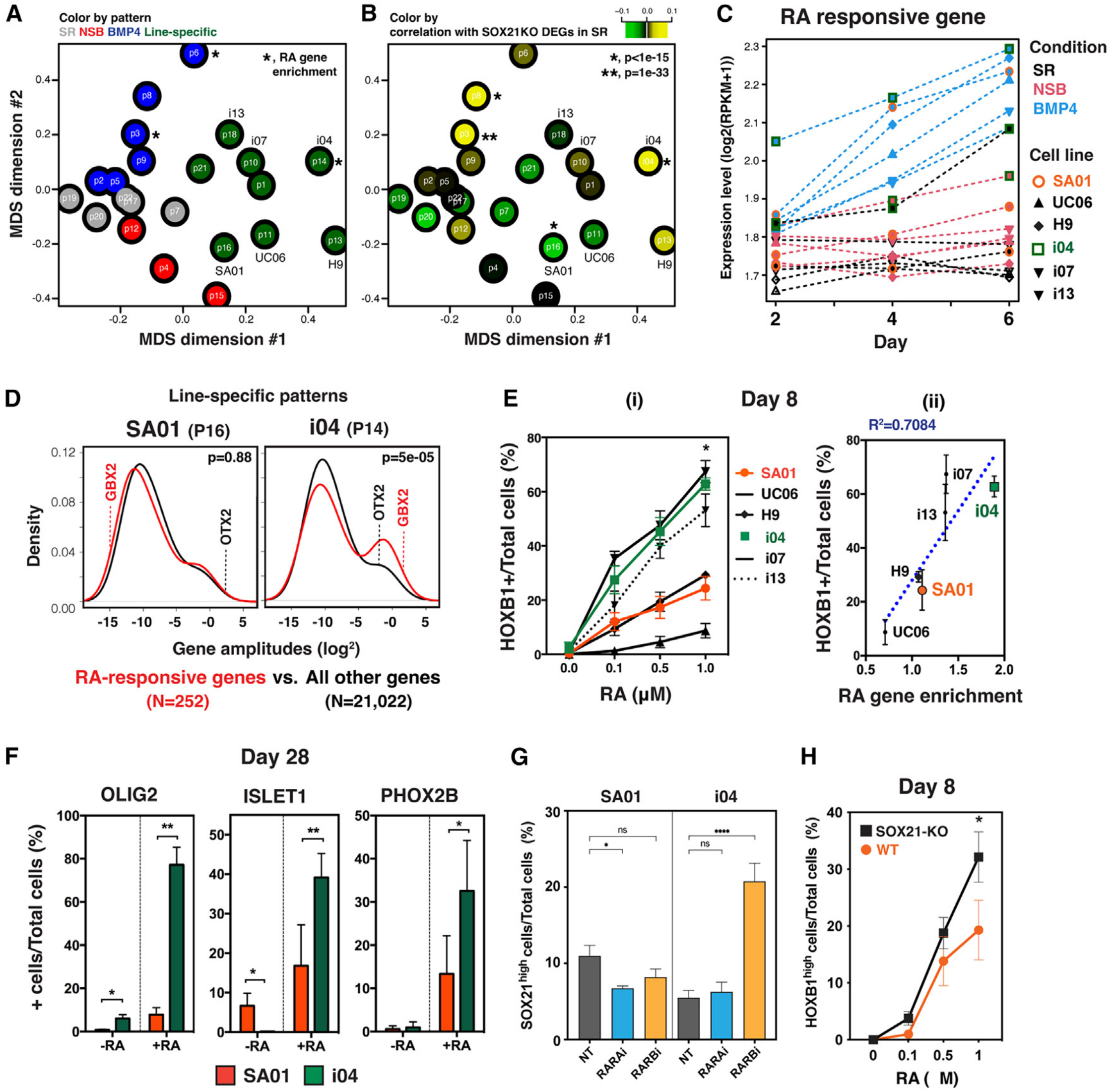

Figure 4. Cell line-specific transcriptomic signatures underlie variation in forebrain versus hindbrain fate bias

(A) MDS plot of gene amplitudes showing correlation between GWCoGAPS-I patterns. *, patterns with RA-responsive gene enrichment (P3, $p = 9.7e{-}08$; P6, $p = 0.014$; P14, $p = 0.008$).

(B) MDS plot colored by correlation of each pattern's gene weights with DEGs in SOX21-KO cells in SR condition.

(C) Average expression of RA genes.

(D) Distribution of gene weights of RA genes in cell line-specific patterns.

(E) (i) Proportion of $HOXB1^{hi}$ cells after RA treatment in NSB condition. *, comparison between SA01 and i04 ($p < 0.05$). (ii) Correlation of $HOXB1^{hi}$ cell proportions and RA gene enrichment in each cell line-specific pattern. $n = 3$, independent experiments.

(F) Different production of hindbrain neurons in response to RA Comparison between SA01 and i04 (*, $p < 0.05$; **, $p < 0.01$). $n = 3$, independent experiments.

(G) Proportion of $SOX21^{hi}$ cells after RAR inhibitor treatment on D4 in NSB condition (*, $p < 0.05$; ****, $p < 0.0001$). $n = 6$, technical replicates.

(H) Proportion of $HOXB1^{hi}$ cells in SOX21-KO lines after RA treatment in NSB condition. Comparison between WT and SOX21-KO (*, $p < 0.05$). $n = 3$, replicate cell lines.

See also Figure S4.

these eQTLs ($p = 1e{-}6$ to $p = 5e{-}21$), suggesting their stable expression. To assess the genetic origins of these stable signatures, we compared the strength of donor-specific signatures in brain RNA-seq data with genetic similarity among donors, using single-nucleotide polymorphism genotypes in these brains (Jaffe et al., 2018)(Figure S5B). We observed

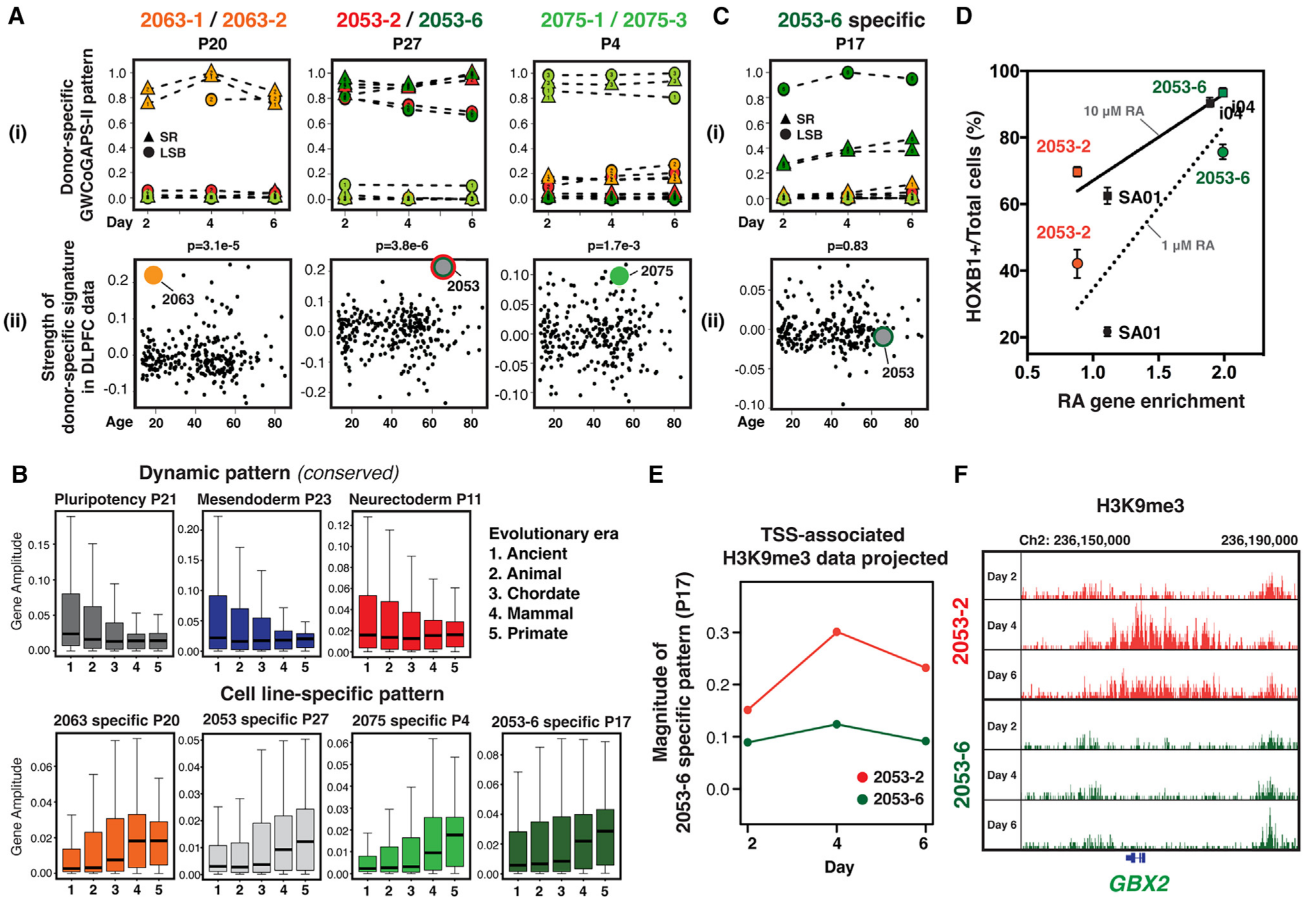

Figure 5. Genetic and epigenetic elements contribute to donor- and line-specific transcriptomic signatures

(A) (i) Donor-specific patterns. (ii) Projection of 260 human brain data. Significance confirmed by permutation: 2,053, $p = 3.8e{-}6$; 2075, $p = 1.7e{-}3$; 2063, $p = 3.1e{-}5$.

(B) Contribution of genes of different evolutionary eras to GWCoGAPS-II patterns. Ancient genes (era 1) show high gene amplitudes in conserved dynamic patterns (compared to era 5, Wilcoxon rank-sum test: $p < 1e{-}16$ for all 3 dynamic patterns). Primate-specific genes (era 5) show higher gene amplitudes in cell line-specific patterns ($p < 1e{-}16$ for all 4 line-specific patterns).

(C) 2053-6 line-specific pattern and projection of brain data.

(D) Correlation of $HOXB1^{hi}$ cell proportions and RA gene enrichment in cell line-specific patterns ($R^2 = 0.75$ in 1 μM RA and $R^2 = 0.87$ in 10 μM RA). The proportion in line 2053-2 was correlated with 2053 donor-specific pattern. $n = 3$, independent experiments.

(E) Projection of ChIP-seq data from lines 2053-2 and 2053-6 in SR into the 2053-6 line-specific pattern.

(F) H3K9me3 ChIP-seq data at the *GBX2* locus.

See also Figure S5.

significant correlations ($p = 4.5e{-}7$ and $p = 2.5e{-}8$) between genetic similarity and the strength of the projected donor-specific signatures, indicating that genetic factors influence donor-specific transcriptomic signatures.

Next, we analyzed the distribution of evolutionary gene ages across the gene weights of GWCoGAPS-II patterns. This revealed that dynamic patterns shared across all lines had stronger weights in ancient genes (Figure 5B). In contrast, dynamic patterns varying among lines exhibited a higher contribution of newer genes (Figure S5C). Importantly, evolutionarily recent genes showed the highest contribution in cell line-specific patterns (Figures 5B and S5D). These findings suggest that conserved dynamic changes in pluripotency and differentiation are ancient and part of the "Waddington landscape" (Ferrell, 2012) that constrains cellular differentiation paths. In contrast, stable transcriptomic patterns related to individual human variation are newer and influence how cells of individual hPSC lines follow particular paths within this cellular landscape.

Additionally, estimates of gene dosage sensitivity (Collins et al., 2022) indicate that genes in cell line-specific signatures are less crucial to survival, reflecting their recent evolutionary origin. We found that the top 1% of each

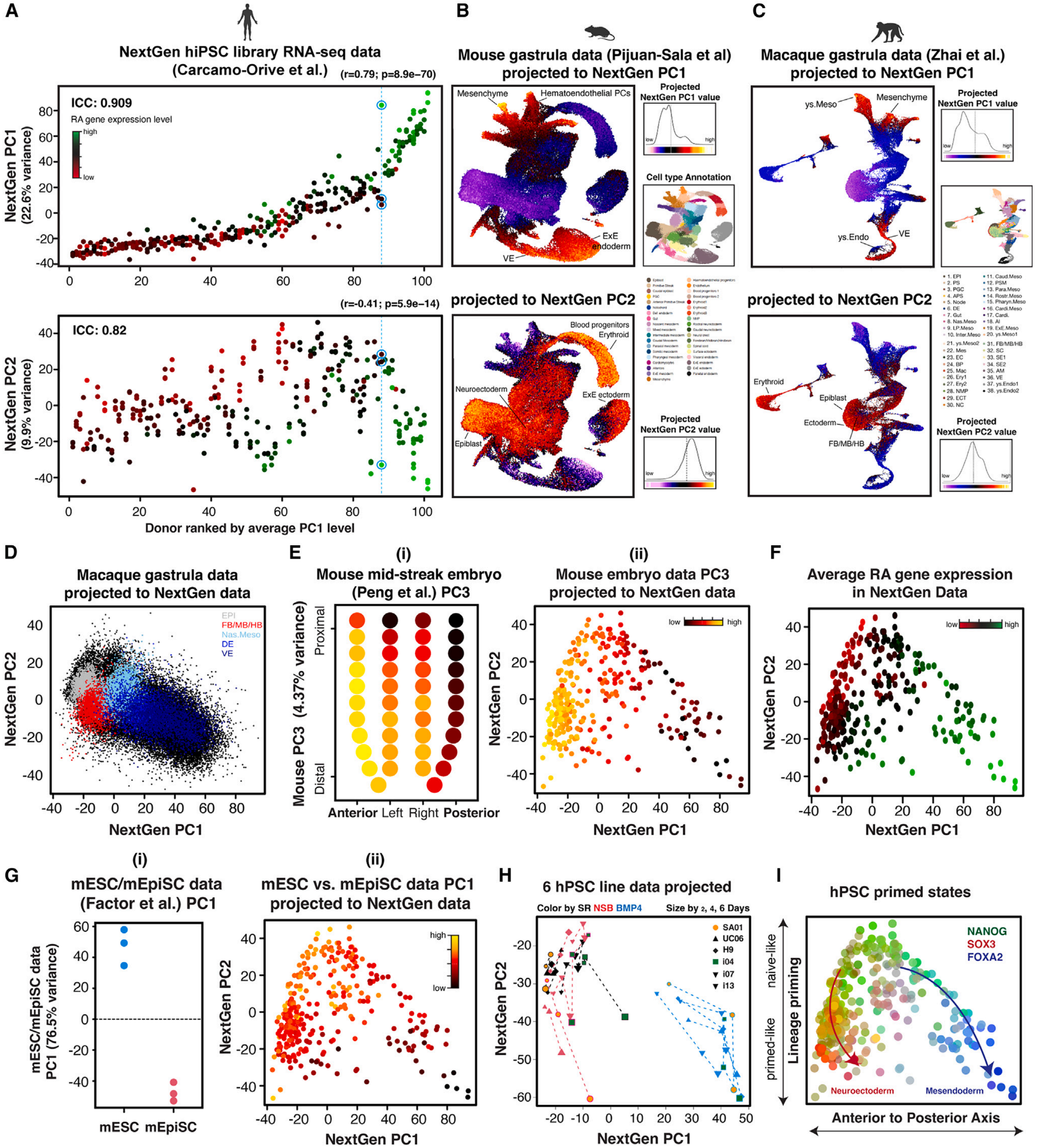

Figure 6. Early developmental bias and expression of RA-responsive genes define hPSC variation in the human population

(A) PCs of the NextGen RNA-seq data. Donors are ordered along the X axis by the average PC1 level of all replicates. Pearson's R and *p* values indicate correlations of PC1 and PC2 with mean RA gene expression. Blue circles highlight lines from one donor with high variance in PCs and RA gene expression. Intraclass correlation coefficient (ICC) estimates proportion of transcriptomic variation across lines attributed to the donor of origin.

(B) NextGen PC projection into mouse gastrula data.

(legend continued on next page)

GWCoGAPS-I pattern had lower gene dosage sensitivity in cell line-specific patterns (average probability of haploinsufficiency, pHaplo; 0.32–0.47) compared to other patterns (pHaplo; 0.53–0.72).

Cell line-specific signatures can also be driven by early epigenetic mechanisms

In addition to donor-specific signatures (Figure 5A), GWCoGAPS-II revealed a 2053-6 line-specific signature, which was not present in the brain data of the same donor (Figure 5C). Projection of the 2053-6 line-specific signature showed no correlation with genetic distance between donors (Figure S5B), suggesting that cell line-specific patterns can dissect distinct genetic and epigenetic origins.

Projection of the new cell line RNA-seq data into the GWCoGAPS-I neuroectodermal patterns revealed that all new lines showed similar forebrain fate induction, except 2053-6 (Figure S5E). Line 2053-6 showed less SOX21 induction than 2053-2 (Figure S5F), similar to the differences between SA01 and i04. RA-responsive genes were enriched in the 2053-6 line-specific pattern, and 2053-6 line generated more hindbrain cells following RA treatment (Figure S5G). These data indicate that the decision to preferentially form fore- versus hindbrain fates between 2053-2 and -6 lines may be regulated by stable epigenetic differences.

Genome sequencing was performed on 2053-2 and -6 lines and brain tissues from donors 2053 and 2075 (Figure S5H). For both donors, most copy-number variations were shared in all donor tissues and cell lines, indicating minimal genetic changes during reprogramming. Therefore, the discordant expression traits and lineage bias between 2053-2 and -6 lines were not due to large-scale genome differences.

Notably, we found that Kruppel-associated box domain zinc finger (KRAB-ZNF) genes were significantly enriched in all line- and donor-specific signatures (Table S2) and expressed at distinct levels among lines (Figures S5I and S5J). KRAB-ZNF genes repress transposable elements and establish persistent H3K9me3-mediated heterochromatin to regulate gene expression during early development (Ecco et al., 2016). These observations suggest that early KRAB-ZNF-driven H3K9me3 heterochromatin mechanisms in pluripotency may shape persisting transcriptomic phenotypes that influence cell function.

To explore this further, we generated chromatin immunoprecipitation sequencing (ChIP-seq) data for histone modification from 2053-2 and 2053-6 lines in SR condition. Projection into the 2053-6 line-specific pattern showed enrichment of the H3K9me3 repressive mark in 2053-2 at promoters of genes overexpressed in 2053-6, while H3K4me3 and H3K27me3 showed no such difference (Figures 5E and S5K). This suggests that many genes in 2053-6 have been de-repressed via specific loss of H3K9me3, leading to stable expression phenotypes and posterior fate bias. In particular, *GBX2* was highly represented in the 2053-6 line-specific signature and showed higher H3K9me3 levels in 2053-2 (Figures 5F and S5L; Table S3). This distinct heterochromatin setting between the lines in pluripotency underscores the importance of exploring how early epigenetic mechanisms contribute to divergent transcriptomic phenotypes and lineage fate bias.

Early lineage bias and RA signaling define hPSC variation in the wider human population

To explore the generality of these features in the human population, we performed PCA on RNA-seq data from 317 undifferentiated hiPSC lines derived from 101 donors generated by the NextGen Consortium (Carcamo-Orive et al., 2017). PC1 exhibited a strong positive correlation with RA-responsive gene expression, while PC2 showed a negative correlation, indicating that the differences in RA response observed in a small number of lines reflect broader systematic variation (Figure 6A). A significant proportion of variance in PC1 (91%) and PC2 (82%) was derived from inter-donor differences, suggesting that genetic factors play a dominant role in the transcriptomic variation across hPSC lines. In contrast, certain hiPSC lines from the same donor displayed significantly divergent positions along PC1 and PC2, as well as distinct RA-responsive gene expression levels, suggesting that epigenetic mechanisms also contribute to this transcriptomic variation.

Projection of the NextGen PCs onto early mouse development revealed a strong lineage bias (Figure 6B). High PC1 values corresponded to mesodermal and endodermal identities. RA-responsive genes with the highest PC1 loadings were highly expressed in self-renewing cells at the edge of pluripotent colonies (Hough et al., 2014) and in

(C and D) NextGen PC projections into macaque gastrula data.
(E) (i) PC3 of the mouse (embryonic day 7) embryo data. (ii) Projection into the NextGen PCs.
(F) Average RA gene expression in NextGen PCs.
(G) (i) PC1 represents higher gene expression in mESCs. (ii) Projection into NextGen PCs.
(H) Projection of 6 hPSC line data into NextGen PCs.
(I) A model illustrating how biased gene expression in hPSCs drives (arrows) anterior/neuroectodermal or posterior/mesendodermal differentiation.
See also Figure S6.

pluripotent founder cells with primitive endoderm identities (Nakanishi et al., 2019). High PC2 values were associated with epiblast, primitive streak, and hematopoietic lineage identities. These lineage associations were also observed when projecting the NextGen PCs onto macaque gastrula data (Zhai et al., 2022) (Figure 6C). Projecting macaque epiblast and early lineage data into NextGen PCs revealed a systematic distribution of lineage bias within hPSC variation (Figures 6D and S6A). Cell lines with high PC1 values displayed high expression of mesenchyme, erythroid, and endothelium-specific genes in human embryos, while cell lines with low PC1 and PC2 values exhibited high expression of neural progenitor genes (Figure S6B) (Zeng et al., 2023).

These early lineage features were further explored by projecting GWCoGAPS-I dynamic patterns (Figure S6C). Cell lines with low PC1 values exhibited higher levels of pluripotency patterns, while those with high PC1 values showed higher levels of mesendoderm patterns. Lines with low PC1 and PC2 values displayed higher levels of neuroectoderm patterns. The strong associations with A-P regional identities were validated by projecting mouse embryo data (Peng et al., 2016) (Figure 6E; Table S4). Consistently, the lines showing posterior bias exhibited higher expression levels of RA-responsive genes (Figure 6F; Table S4). These findings indicate that the transcriptomic heterogeneity in hPSC lines aligns with the developmental A-P axis and lineage emergence in mammalian embryos.

Interestingly, projecting mouse inner cell mass-derived embryonic stem cell (mESC) and epiblast-derived stem cell (mEpiSC) data (Factor et al., 2014) revealed that cell lines with high NextGen PC2 values displayed a higher mESC identity (Figure 6G; Table S4). This suggests that these PSC line variations exhibit gradients of naive and primed pluripotent transcriptomic signatures (Figures 6G and 6I).

Furthermore, we projected cell line-specific patterns into the NextGen data (Figure S6D). The transcriptomic signatures of SA01 and 2053-2 lines, which exhibited neuroectoderm and forebrain bias, were more pronounced in cell lines with lower NextGen PC1 values, aligning with their anterior bias. In contrast, the signatures of lines i04 and 2053-6, displaying mesendoderm and hindbrain bias, were elevated in lines with higher NextGen PC1 values, reflecting their posterior bias. This analysis further supports that cell line-specific signatures contain transcriptomic traits predicting lineage bias.

Projection of the initial six-line data, including early differentiation conditions (Figure 1C), into NextGen PCs confirmed the lineage structure within the pluripotency landscape (Figure 6H). This analysis clearly separated SR and neuroectoderm from mesendoderm differentiation along NextGen PC1. The progression from SR toward early lineages aligned with Nextgen PC2. These findings, summarized in Figure 6I, emphasize the broad utility of mapping lineage bias in hPSCs as these early cellular assays within pluripotency have the potential to predict functional differences across extensive hPSC line collections.

DISCUSSION

Studies are increasingly revealing the genetic origins of heterogeneity in transcription and differentiation potential of hPSCs (Bonder et al., 2021; Carcamo-Orive et al., 2017; Choi et al., 2015; Cuomo et al., 2020; DeBoever et al., 2017; Jerber et al., 2021; Kilpinen et al., 2017; Kyttala et al., 2016; Merkle et al., 2022; Ortmann et al., 2020; Puigdevall et al., 2023; Rouhani et al., 2014; Strano et al., 2020). Our analysis identifies dynamic lineage-driving transcriptomic modules and cell line-specific gene expression traits. We demonstrated that the relationship between cell line-specific and dynamic patterns enables the identification of key regulators predicting functional phenotypes of PSC lines during differentiation. The observed bias in neural and mesendodermal lineage in a small number of hPSC lines aligned with the transcriptomic variation across hundreds of hPSC lines prior to differentiation, suggesting this variation in lineage bias is a general feature of the human population. Low variance within donors and persistent donor-specific expression traits in adult tissues suggest that major aspects of this transcriptomic variation in pluripotency are long-lasting and genetically controlled. In addition, a difference in bias toward fore- versus hindbrain fates in replicate lines from the same donor indicates that epigenetic mechanisms also contribute to variation in early neural fate choice. These data support a model where, in addition to genetic variation, alternate epigenetically predisposed states exist within pluripotency prior to the implementation of anterior or posterior regional fate choice.

In contrast to the conserved dynamic expression patterns, cell line-specific patterns were enriched with recently evolved genes. KRAB-ZNF gene enrichment and H3K9me3-mediated regulation in these cell line-specific signatures suggest a potential epigenetic mechanism controlling their stable expression phenotypes. Recent studies have revealed subtle variations in H3K9me3 that function in cellular reprogramming and early lineage plasticity (Buckberry et al., 2023; Hoetker et al., 2023). Understanding how H3K9me3, associated with individual signatures, interacts with other cell type-specific histone modifications will provide deeper insight into the epigenetic regulation of human variation in lineage bias.

Recent scRNA-seq data from cerebral organoids (Kanton et al., 2019; Rosebrock et al., 2022) revealed striking differential forebrain fate bias across human donors. Our previous work (Micali et al., 2020) defined early variation

in transcriptomic patterns resulting in a neural fate bias along the dorsoventral telencephalic axis. Variation in dorsoventral specification has been observed across many lines and linked to risk for neuropsychiatric diseases (Mariani et al., 2015; Paulsen et al., 2022; Strano et al., 2020). In this current study, we focused on differential RA signaling, which drives lineage bias along the A-P axis. Dysregulation of RA signaling has been associated with the risk for schizophrenia and autism (Shibata et al., 2021). Further dissecting the consequences of variation in early RA response and other morphogenetic regulators is important to better understand potential clinical implications.

Advances in selecting specific hPSC lines for stem cell therapeutics (Andrews et al., 2022; Merkle et al., 2022) highlight the importance of defining genetic and epigenetic controls influencing human cellular variation during differentiation. Our work contributes to understanding how variation in early cellular states influences human brain development to modify complex traits and disease risk. As PSC-derived models of early mammalian development become increasingly sophisticated (Pera, 2023), using synthetic embryos from diverse PSC lines holds great promise to systematically explore the consequences of this early inherent variation at later steps of human brain development and function.

EXPERIMENTAL PROCEDURES

Resource availability

Lead contact

Further information and requests should be directed to and will be fulfilled by the lead contact Ronald D. McKay (ronaldmckay@mac.com).

Materials availability

Cell lines generated in this study are available from the lead contact upon request.

Data and code availability

The accession number for the RNA-seq data reported in this paper is deposited in GEO Database: GSE164055, with linked raw read files in the National Library of Medicine Sequence Read Archive under BioProject PRJNA688712.

(https://www.ncbi,nlm.nih.gov/sra)

GWCoGAPS decompositions:

(https://www.bioconductor.org/packages/release/bioc/html/CoGAPS.html).

Projection analyses:

(https://www.bioconductor.org/packages/release/bioc/html/projectR.html).

Multi-omics data at NeMO Analytics:

Single gene query:

https://nemoanalytics.org/p?l=Kim2024.

Projection of GWCoGAPS-I:

https://nemoanalytics.org/p?p=p&l=Kim2024&c=Kim2024_GWCoGAPS_I_p24&algo=nmf.

Projection of GWCoGAPS-II:

https://nemoanalytics.org/p?p=p&l=Kim2024&c=Kim2024_GWCoGAPS_II_p30&algo=nmf.

Projection of NextGen Consortium PCA:

https://nemoanalytics.org/p?p=p&l=Kim2024&c=Kim2024_NextGenPCs&algo=pca.

Methods

hPSC culture and differentiation

hPSCs were dissociated with Accutase (A11105, Life Technologies), plated at 1 × 10^5 cells/cm^2 on Matrigel (354277, BD)-coated plates, and cultured in mTeSR1 (05850, Stem Cell Technology) with 5 μM Y27632 (Y0503, Sigma-Aldrich), which was removed after 24 h. Neuroectodermal differentiation was induced with Noggin (500 ng/mL, 719-NG, R&D Systems) and SB431542 (2 μM, S4317, Sigma-Aldrich) in mTeSR1; mesendodermal differentiation was induced with BMP4 (100 ng/mL, 314-BP, R&D Systems) upon Y27632 removal (D0) and cultured for 6 days.

Generation of CRISPR-Cas9-mediated SOX21-KO line

SOX21-KO lines were generated using CRISPR-Cas9. Oligonucleotides were cloned into pSpCas9(BB)-2A-Puro (px459; Addgene), producing plasmid pX459-Sox21NHEJ4 and pX459-Sox21NHEJ5, synthesized by Integrated DNA Technologies. SA01 hESCs were transfected with 2.5 μg of either plasmid using DNA-In Stem (MTI-GlobalStem, gifted from Dr. Jessee).

RNA-seq data processing

After sequencing, Illumina Real Time Analysis (RTA) module was used to perform image analysis and base calling, and BCL Converter (CASAVA v1.8.2) was used to generate FASTQ files. Sequencing depth was over 80 million (40 million paired-end) (Table S1). Read-level Q/C was performed by FastQC (v0.10.1). Pair-end reads of cDNA sequences were aligned back to the human genome (UCSC hg19 from Illumina iGenome) by spliced read mapper TopHat (v2.0.4). Reads were counted by htseq-count v0.5.3 according to gene annotation (Illumina iGenome), and Reads Per Kilobase per Million mapped reads (RPKM) was calculated. This provided 23,368 gene-level expression profiles.

SUPPLEMENTAL INFORMATION

Supplemental information can be found online at https://doi.org/10.1016/j.stemcr.2024.07.004.

ACKNOWLEDGMENTS

We thank the Lieber and Maltz families for their support at the Lieber Institute for Brain Development (LIBD). This work was also supported by NCI/NIH grants R01CA177669, P30CA006973, U01CA212007, and U01CA253403 and Johns Hopkins University Catalyst Award to E.J.F., and R01NS116418, R01HG010898, MH116488, and U01MH124619 to N.S. Data sharing and visualization via NeMO Analytics were supported by grants R24MH114815 and R01DC019370. We thank J. Jessee, MTI-GlobalStem, for technical support on CRISPR-Cas9 plasmid transfection. We thank many members at LIBD and Sestan lab for their helpful comments.

AUTHOR CONTRIBUTIONS

S.-K.K., S.S., G.S.-O., A.J., J.G.C., D.J.H., C.C., and R.D.M. conceived the study. S.-K.K., S.S., and Y.W. performed cell culture. K.O. generated SOX21-KO lines. S.-K.K., A.J., Y.W., and K.O. generated RNA-seq data. G.S.-O., S.S., E.J.F., C.C., and J.-H.S. analyzed RNA-seq and ChIP-seq data. V.L. and A.K. performed gene age analyses. T.M.H., J.E.K., and D.R.W. provided fibroblasts and human brain data. S.-K.K., S.S., N.M., and H.K. performed immunocytochemistry. S.-K.K., S.S., A.J., T.V., and D.J.H. performed image analysis. S.-K.K., S.S., G.S.-O., N.M., J.G.C., D.J.H., N.S., C.C., and R.D.M. interpreted the data. N.S., R.B., A.J.C., N.J.B., D.R.W., and R.D.M. directed the research. S.-K.K., S.S., C.C., and R.D.M. wrote the manuscript.

DECLARATION OF INTERESTS

The authors declare no competing interests.

Received: September 18, 2023
Revised: July 16, 2024
Accepted: July 16, 2024
Published: August 15, 2024

REFERENCES

Andrews, P.W., Barbaric, I., Benvenisty, N., Draper, J.S., Ludwig, T., Merkle, F.T., Sato, Y., Spits, C., Stacey, G.N., Wang, H., and Pera, M.F. (2022). The consequences of recurrent genetic and epigenetic variants in human pluripotent stem cells. Cell Stem Cell *29*, 1624–1636. https://doi.org/10.1016/j.stem.2022.11.006.

Arnold, S.J., and Robertson, E.J. (2009). Making a commitment: cell lineage allocation and axis patterning in the early mouse embryo. Nat. Rev. Mol. Cell Biol. *10*, 91–103. https://doi.org/10.1038/nrm2618.

Balmer, J.E., and Blomhoff, R. (2002). Gene expression regulation by retinoic acid. J. Lipid Res. *43*, 1773–1808. https://doi.org/10.1194/jlr.R100015-JLR200.

Bao, S., Tang, F., Li, X., Hayashi, K., Gillich, A., Lao, K., and Surani, M.A. (2009). Epigenetic reversion of post-implantation epiblast to pluripotent embryonic stem cells. Nature *461*, 1292–1295. https://doi.org/10.1038/nature08534.

Bonder, M.J., Smail, C., Gloudemans, M.J., Frésard, L., Jakubosky, D., D'Antonio, M., Li, X., Ferraro, N.M., Carcamo-Orive, I., Mirauta, B., et al. (2021). Identification of rare and common regulatory variants in pluripotent cells using population-scale transcriptomics. Nat. Genet. *53*, 313–321. https://doi.org/10.1038/s41588-021-00800-7.

Brons, I.G.M., Smithers, L.E., Trotter, M.W.B., Rugg-Gunn, P., Sun, B., Chuva de Sousa Lopes, S.M., Howlett, S.K., Clarkson, A., Ahrlund-Richter, L., Pedersen, R.A., and Vallier, L. (2007). Derivation of pluripotent epiblast stem cells from mammalian embryos. Nature *448*, 191–195. https://doi.org/10.1038/nature05950.

Buckberry, S., Liu, X., Poppe, D., Tan, J.P., Sun, G., Chen, J., Nguyen, T.V., de Mendoza, A., Pflueger, J., Frazer, T., et al. (2023). Transient naive reprogramming corrects hiPS cells functionally and epigenetically. Nature *620*, 863–872. https://doi.org/10.1038/s41586-023-06424-7.

Burke, E.E., Chenoweth, J.G., Shin, J.H., Collado-Torres, L., Kim, S.K., Micali, N., Wang, Y., Colantuoni, C., Straub, R.E., Hoeppner, D.J., et al. (2020). Dissecting transcriptomic signatures of neuronal differentiation and maturation using iPSCs. Nat. Commun. *11*, 462. https://doi.org/10.1038/s41467-019-14266-z.

Carcamo-Orive, I., Hoffman, G.E., Cundiff, P., Beckmann, N.D., D'Souza, S.L., Knowles, J.W., Patel, A., Papatsenko, D., Abbasi, F., Reaven, G.M., et al. (2017). Analysis of Transcriptional Variability in a Large Human iPSC Library Reveals Genetic and Non-genetic Determinants of Heterogeneity. Cell Stem Cell *20*, 518–532.e9. https://doi.org/10.1016/j.stem.2016.11.005.

Chambers, S.M., Fasano, C.A., Papapetrou, E.P., Tomishima, M., Sadelain, M., and Studer, L. (2009). Highly efficient neural conversion of human ES and iPS cells by dual inhibition of SMAD signaling. Nat. Biotechnol. *27*, 275–280. https://doi.org/10.1038/nbt.1529.

Choi, J., Lee, S., Mallard, W., Clement, K., Tagliazucchi, G.M., Lim, H., Choi, I.Y., Ferrari, F., Tsankov, A.M., Pop, R., et al. (2015). A comparison of genetically matched cell lines reveals the equivalence of human iPSCs and ESCs. Nat. Biotechnol. *33*, 1173–1181. https://doi.org/10.1038/nbt.3388.

Collins, R.L., Glessner, J.T., Porcu, E., Lepamets, M., Brandon, R., Lauricella, C., Han, L., Morley, T., Niestroj, L.M., Ulirsch, J., et al. (2022). A cross-disorder dosage sensitivity map of the human genome. Cell *185*, 3041–3055.e25. https://doi.org/10.1016/j.cell.2022.06.036.

Cuomo, A.S.E., Seaton, D.D., McCarthy, D.J., Martinez, I., Bonder, M.J., Garcia-Bernardo, J., Amatya, S., Madrigal, P., Isaacson, A., Buettner, F., et al. (2020). Single-cell RNA-sequencing of differentiating iPS cells reveals dynamic genetic effects on gene expression. Nat. Commun. *11*, 810. https://doi.org/10.1038/s41467-020-14457-z.

DeBoever, C., Li, H., Jakubosky, D., Benaglio, P., Reyna, J., Olson, K.M., Huang, H., Biggs, W., Sandoval, E., D'Antonio, M., et al. (2017). Large-Scale Profiling Reveals the Influence of Genetic Variation on Gene Expression in Human Induced Pluripotent Stem Cells. Cell Stem Cell *20*, 533–546.e7. https://doi.org/10.1016/j.stem.2017.03.009.

Ecco, G., Cassano, M., Kauzlaric, A., Duc, J., Coluccio, A., Offner, S., Imbeault, M., Rowe, H.M., Turelli, P., and Trono, D. (2016). Transposable Elements and Their KRAB-ZFP Controllers Regulate Gene Expression in Adult Tissues. Dev. Cell *36*, 611–623. https://doi.org/10.1016/j.devcel.2016.02.024.

Factor, D.C., Corradin, O., Zentner, G.E., Saiakhova, A., Song, L., Chenoweth, J.G., McKay, R.D., Crawford, G.E., Scacheri, P.C., and Tesar, P.J. (2014). Epigenomic Comparison Reveals Activation of "Seed" Enhancers during Transition from Naive to Primed Pluripotency. Cell Stem Cell *14*, 854–863. https://doi.org/10.1016/j.stem.2014.05.005.

Faial, T., Bernardo, A.S., Mendjan, S., Diamanti, E., Ortmann, D., Gentsch, G.E., Mascetti, V.L., Trotter, M.W.B., Smith, J.C., and Pedersen, R.A. (2015). Brachyury and SMAD signalling collaboratively orchestrate distinct mesoderm and endoderm gene regulatory

networks in differentiating human embryonic stem cells. Development *142*, 2121–2135. https://doi.org/10.1242/dev.117838.

Fang, Z., Liu, X., Wen, J., Tang, F., Zhou, Y., Jing, N., and Jin, Y. (2019). SOX21 Ensures Rostral Forebrain Identity by Suppression of WNT8B during Neural Regionalization of Human Embryonic Stem Cells. Stem Cell Rep. *13*, 1038–1052. https://doi.org/10.1016/j.stemcr.2019.10.013.

Ferrell, J.E., Jr. (2012). Bistability, bifurcations, and Waddington's epigenetic landscape. Curr. Biol. *22*, R458–R466. https://doi.org/10.1016/j.cub.2012.03.045.

Ghyselinck, N.B., and Duester, G. (2019). Retinoic acid signaling pathways. Development *146*, dev167502. https://doi.org/10.1242/dev.167502.

Gogolou, A., Souilhol, C., Granata, I., Wymeersch, F.J., Manipur, I., Wind, M., Frith, T.J.R., Guarini, M., Bertero, A., Bock, C., et al. (2022). Early anteroposterior regionalisation of human neural crest is shaped by a pro-mesodermal factor. Elife *11*, e74263. https://doi.org/10.7554/eLife.74263.

Goolam, M., Scialdone, A., Graham, S.J.L., Macaulay, I.C., Jedrusik, A., Hupalowska, A., Voet, T., Marioni, J.C., and Zernicka-Goetz, M. (2016). Heterogeneity in Oct4 and Sox2 Targets Biases Cell Fate in 4-Cell Mouse Embryos. Cell *165*, 61–74. https://doi.org/10.1016/j.cell.2016.01.047.

GTEx Consortium (2015). The Genotype-Tissue Expression (GTEx) pilot analysis: Multitissue gene regulation in humans. Science *348*, 648–660.

Guibentif, C., Griffiths, J.A., Imaz-Rosshandler, I., Ghazanfar, S., Nichols, J., Wilson, V., Göttgens, B., and Marioni, J.C. (2021). Diverse Routes toward Early Somites in the Mouse Embryo. Dev. Cell *56*, 141–153.e6. https://doi.org/10.1016/j.devcel.2020.11.013.

Guo, G., Stirparo, G.G., Strawbridge, S.E., Spindlow, D., Yang, J., Clarke, J., Dattani, A., Yanagida, A., Li, M.A., Myers, S., et al. (2021). Human naive epiblast cells possess unrestricted lineage potential. Cell Stem Cell *28*, 1040–1056.e6. https://doi.org/10.1016/j.stem.2021.02.025.

Henrique, D., Abranches, E., Verrier, L., and Storey, K.G. (2015). Neuromesodermal progenitors and the making of the spinal cord. Development *142*, 2864–2875. https://doi.org/10.1242/dev.119768.

Hoetker, M.S., Yagi, M., Di Stefano, B., Langerman, J., Cristea, S., Wong, L.P., Huebner, A.J., Charlton, J., Deng, W., Haggerty, C., et al. (2023). H3K36 methylation maintains cell identity by regulating opposing lineage programmes. Nat. Cell Biol. *25*, 1121–1134. https://doi.org/10.1038/s41556-023-01191-z.

Hough, S.R., Thornton, M., Mason, E., Mar, J.C., Wells, C.A., and Pera, M.F. (2014). Single-Cell Gene Expression Profiles Define Self-Renewing, Pluripotent, and Lineage Primed States of Human Pluripotent Stem Cells. Stem Cell Rep. *2*, 881–895. https://doi.org/10.1016/j.stemcr.2014.04.014.

Jaffe, A.E., Straub, R.E., Shin, J.H., Tao, R., Gao, Y., Collado-Torres, L., Kam-Thong, T., Xi, H.S., Quan, J., Chen, Q., et al. (2018). Developmental and genetic regulation of the human cortex transcriptome illuminate schizophrenia pathogenesis. Nat. Neurosci. *21*, 1117–1125. https://doi.org/10.1038/s41593-018-0197-y.

Jerber, J., Seaton, D.D., Cuomo, A.S.E., Kumasaka, N., Haldane, J., Steer, J., Patel, M., Pearce, D., Andersson, M., Bonder, M.J., et al. (2021). Population-scale single-cell RNA-seq profiling across dopaminergic neuron differentiation. Nat. Genet. *53*, 304–312. https://doi.org/10.1038/s41588-021-00801-6.

Jourdon, A., Wu, F., Mariani, J., Capauto, D., Norton, S., Tomasini, L., Amiri, A., Suvakov, M., Schreiner, J.D., Jang, Y., et al. (2023). Modeling idiopathic autism in forebrain organoids reveals an imbalance of excitatory cortical neuron subtypes during early neurogenesis. Nat. Neurosci. *26*, 1505–1515. https://doi.org/10.1038/s41593-023-01399-0.

Kanton, S., Boyle, M.J., He, Z., Santel, M., Weigert, A., Sanchís-Calleja, F., Guijarro, P., Sidow, L., Fleck, J.S., Han, D., et al. (2019). Organoid single-cell genomic atlas uncovers human-specific features of brain development. Nature *574*, 418–422. https://doi.org/10.1038/s41586-019-1654-9.

Kilpinen, H., Goncalves, A., Leha, A., Afzal, V., Alasoo, K., Ashford, S., Bala, S., Bensaddek, D., Casale, F.P., Culley, O.J., et al. (2017). Common genetic variation drives molecular heterogeneity in human iPSCs. Nature *546*, 370–375. https://doi.org/10.1038/nature22403.

Kuzmichev, A.N., Kim, S.K., D'Alessio, A.C., Chenoweth, J.G., Wittko, I.M., Campanati, L., and McKay, R.D. (2012). Sox2 acts through Sox21 to regulate transcription in pluripotent and differentiated cells. Curr. Biol. *22*, 1705–1710. https://doi.org/10.1016/j.cub.2012.07.013.

Kyttala, A., Moraghebi, R., Valensisi, C., Kettunen, J., Andrus, C., Pasumarthy, K.K., Nakanishi, M., Nishimura, K., Ohtaka, M., Weltner, J., et al. (2016). Genetic Variability Overrides the Impact of Parental Cell Type and Determines iPSC Differentiation Potential. Stem Cell Rep. *6*, 200–212. https://doi.org/10.1016/j.stemcr.2015.12.009.

Mallon, B.S., Chenoweth, J.G., Johnson, K.R., Hamilton, R.S., Tesar, P.J., Yavatkar, A.S., Tyson, L.J., Park, K., Chen, K.G., Fann, Y.C., and McKay, R.D.G. (2013). StemCellDB: the human pluripotent stem cell database at the National Institutes of Health. Stem Cell Res. *10*, 57–66. https://doi.org/10.1016/j.scr.2012.09.002.

Mariani, J., Coppola, G., Zhang, P., Abyzov, A., Provini, L., Tomasini, L., Amenduni, M., Szekely, A., Palejev, D., Wilson, M., et al. (2015). FOXG1-Dependent Dysregulation of GABA/Glutamate Neuron Differentiation in Autism Spectrum Disorders. Cell *162*, 375–390. https://doi.org/10.1016/j.cell.2015.06.034.

Matsuda, S., Kuwako, K.i., Okano, H.J., Tsutsumi, S., Aburatani, H., Saga, Y., Matsuzaki, Y., Akaike, A., Sugimoto, H., and Okano, H. (2012). Sox21 promotes hippocampal adult neurogenesis via the transcriptional repression of the Hes5 gene. J. Neurosci. *32*, 12543–12557. https://doi.org/10.1523/JNEUROSCI.5803-11.2012.

Merkle, F.T., Ghosh, S., Genovese, G., Handsaker, R.E., Kashin, S., Meyer, D., Karczewski, K.J., O'Dushlaine, C., Pato, C., Pato, M., et al. (2022). Whole-genome analysis of human embryonic stem cells enables rational line selection based on genetic variation. Cell Stem Cell *29*, 472–486.e7. https://doi.org/10.1016/j.stem.2022.01.011.

Micali, N., Kim, S.-K., Diaz-Bustamante, M., Stein-O'Brien, G., Seo, S., Shin, J.-H., Rash, B.G., Ma, S., Wang, Y., Olivares, N.A., et al. (2020). Variation of Human Neural Stem Cells Generating Organizer States In Vitro before Committing to Cortical Excitatory or

Inhibitory Neuronal Fates. Cell Rep. *31*, 107599. https://doi.org/10.1016/j.celrep.2020.107599.

Millet, S., Campbell, K., Epstein, D.J., Losos, K., Harris, E., and Joyner, A.L. (1999). A role for Gbx2 in repression of Otx2 and positioning the mid/hindbrain organizer. Nature *401*, 161–164.

Nakanishi, M., Mitchell, R.R., Benoit, Y.D., Orlando, L., Reid, J.C., Shimada, K., Davidson, K.C., Shapovalova, Z., Collins, T.J., Nagy, A., and Bhatia, M. (2019). Human Pluripotency Is Initiated and Preserved by a Unique Subset of Founder Cells. Cell *177*, 910–924.e22. https://doi.org/10.1016/j.cell.2019.03.013.

Ortmann, D., Brown, S., Czechanski, A., Aydin, S., Muraro, D., Huang, Y., Tomaz, R.A., Osnato, A., Canu, G., Wesley, B.T., et al. (2020). Naive Pluripotent Stem Cells Exhibit Phenotypic Variability that Is Driven by Genetic Variation. Cell Stem Cell *27*, 470–481.e6. https://doi.org/10.1016/j.stem.2020.07.019.

Paulsen, B., Velasco, S., Kedaigle, A.J., Pigoni, M., Quadrato, G., Deo, A.J., Adiconis, X., Uzquiano, A., Sartore, R., Yang, S.M., et al. (2022). Autism genes converge on asynchronous development of shared neuron classes. Nature *602*, 268–273. https://doi.org/10.1038/s41586-021-04358-6.

Peng, G., Suo, S., Chen, J., Chen, W., Liu, C., Yu, F., Wang, R., Chen, S., Sun, N., Cui, G., et al. (2016). Spatial Transcriptome for the Molecular Annotation of Lineage Fates and Cell Identity in Mid-gastrula Mouse Embryo. Dev. Cell *36*, 681–697. https://doi.org/10.1016/j.devcel.2016.02.020.

Pera, M.F. (2023). Seven days in the life cycle of Homo sapiens. Cell *186*, 3755–3757. https://doi.org/10.1016/j.cell.2023.08.007.

Pijuan-Sala, B., Griffiths, J.A., Guibentif, C., Hiscock, T.W., Jawaid, W., Calero-Nieto, F.J., Mulas, C., Ibarra-Soria, X., Tyser, R.C.V., Ho, D.L.L., et al. (2019). A single-cell molecular map of mouse gastrulation and early organogenesis. Nature *566*, 490–495. https://doi.org/10.1038/s41586-019-0933-9.

Puigdevall, P., Jerber, J., Danecek, P., Castellano, S., and Kilpinen, H. (2023). Somatic mutations alter the differentiation outcomes of iPSC-derived neurons. Cell Genom. *3*, 100280. https://doi.org/10.1016/j.xgen.2023.100280.

Rifes, P., Isaksson, M., Rathore, G.S., Aldrin-Kirk, P., Møller, O.K., Barzaghi, G., Lee, J., Egerod, K.L., Rausch, D.M., Parmar, M., et al. (2020). Modeling neural tube development by differentiation of human embryonic stem cells in a microfluidic WNT gradient. Nat. Biotechnol. *38*, 1265–1273. https://doi.org/10.1038/s41587-020-0525-0.

Rosebrock, D., Arora, S., Mutukula, N., Volkman, R., Gralinska, E., Balaskas, A., Aragonés Hernández, A., Buschow, R., Brändl, B., Müller, F.J., et al. (2022). Enhanced cortical neural stem cell identity through short SMAD and WNT inhibition in human cerebral organoids facilitates emergence of outer radial glial cells. Nat. Cell Biol. *24*, 981–995. https://doi.org/10.1038/s41556-022-00929-5.

Rouhani, F., Kumasaka, N., de Brito, M.C., Bradley, A., Vallier, L., and Gaffney, D. (2014). Genetic background drives transcriptional variation in human induced pluripotent stem cells. PLoS Genet. *10*, e1004432. https://doi.org/10.1371/journal.pgen.1004432.

Shibata, M., Pattabiraman, K., Lorente-Galdos, B., Andrijevic, D., Kim, S.K., Kaur, N., Muchnik, S.K., Xing, X., Santpere, G., Sousa, A.M.M., and Sestan, N. (2021). Regulation of prefrontal patterning and connectivity by retinoic acid. Nature *598*, 483–488. https://doi.org/10.1038/s41586-021-03953-x.

Stein-O'Brien, G.L., Carey, J.L., Lee, W.S., Considine, M., Favorov, A.V., Flam, E., Guo, T., Li, S., Marchionni, L., Sherman, T., et al. (2017). PatternMarkers & GWCoGAPS for novel data-driven biomarkers via whole transcriptome NMF. Bioinformatics *33*, 1892–1894. https://doi.org/10.1093/bioinformatics/btx058.

Strano, A., Tuck, E., Stubbs, V.E., and Livesey, F.J. (2020). Variable Outcomes in Neural Differentiation of Human PSCs Arise from Intrinsic Differences in Developmental Signaling Pathways. Cell Rep. *31*, 107732. https://doi.org/10.1016/j.celrep.2020.107732.

Tesar, P.J., Chenoweth, J.G., Brook, F.A., Davies, T.J., Evans, E.P., Mack, D.L., Gardner, R.L., and McKay, R.D.G. (2007). New cell lines from mouse epiblast share defining features with human embryonic stem cells. Nature *448*, 196–199. https://doi.org/10.1038/nature05972.

Wang, M., Wei, P.C., Lim, C.K., Gallina, I.S., Marshall, S., Marchetto, M.C., Alt, F.W., and Gage, F.H. (2020). Increased Neural Progenitor Proliferation in a hiPSC Model of Autism Induces Replication Stress-Associated Genome Instability. Cell Stem Cell *26*, 221–233.e6. https://doi.org/10.1016/j.stem.2019.12.013.

Warmflash, A., Sorre, B., Etoc, F., Siggia, E.D., and Brivanlou, A.H. (2014). A method to recapitulate early embryonic spatial patterning in human embryonic stem cells. Nat. Methods *11*, 847–854. https://doi.org/10.1038/nmeth.3016.

Zeng, B., Liu, Z., Lu, Y., Zhong, S., Qin, S., Huang, L., Zeng, Y., Li, Z., Dong, H., Shi, Y., et al. (2023). The single-cell and spatial transcriptional landscape of human gastrulation and early brain development. Cell Stem Cell *30*, 851–866.e7. https://doi.org/10.1016/j.stem.2023.04.016.

Zhai, J., Guo, J., Wan, H., Qi, L., Liu, L., Xiao, Z., Yan, L., Schmitz, D.A., Xu, Y., Yu, D., et al. (2022). Primate gastrulation and early organogenesis at single-cell resolution. Nature *612*, 732–738. https://doi.org/10.1038/s41586-022-05526-y.

Stem Cell Reports, Volume *19*

Supplemental Information

Individual variation in the emergence of anterior-to-posterior neural fates from human pluripotent stem cells

Suel-Kee Kim, Seungmae Seo, Genevieve Stein-O'Brien, Amritha Jaishankar, Kazuya Ogawa, Nicola Micali, Victor Luria, Amir Karger, Yanhong Wang, Hyojin Kim, Thomas M. Hyde, Joel E. Kleinman, Ty Voss, Elana J. Fertig, Joo-Heon Shin, Roland Bürli, Alan J. Cross, Nicholas J. Brandon, Daniel R. Weinberger, Joshua G. Chenoweth, Daniel J. Hoeppner, Nenad Sestan, Carlo Colantuoni, and Ronald D. McKay

Supplemental Materials

Supplemental Figures

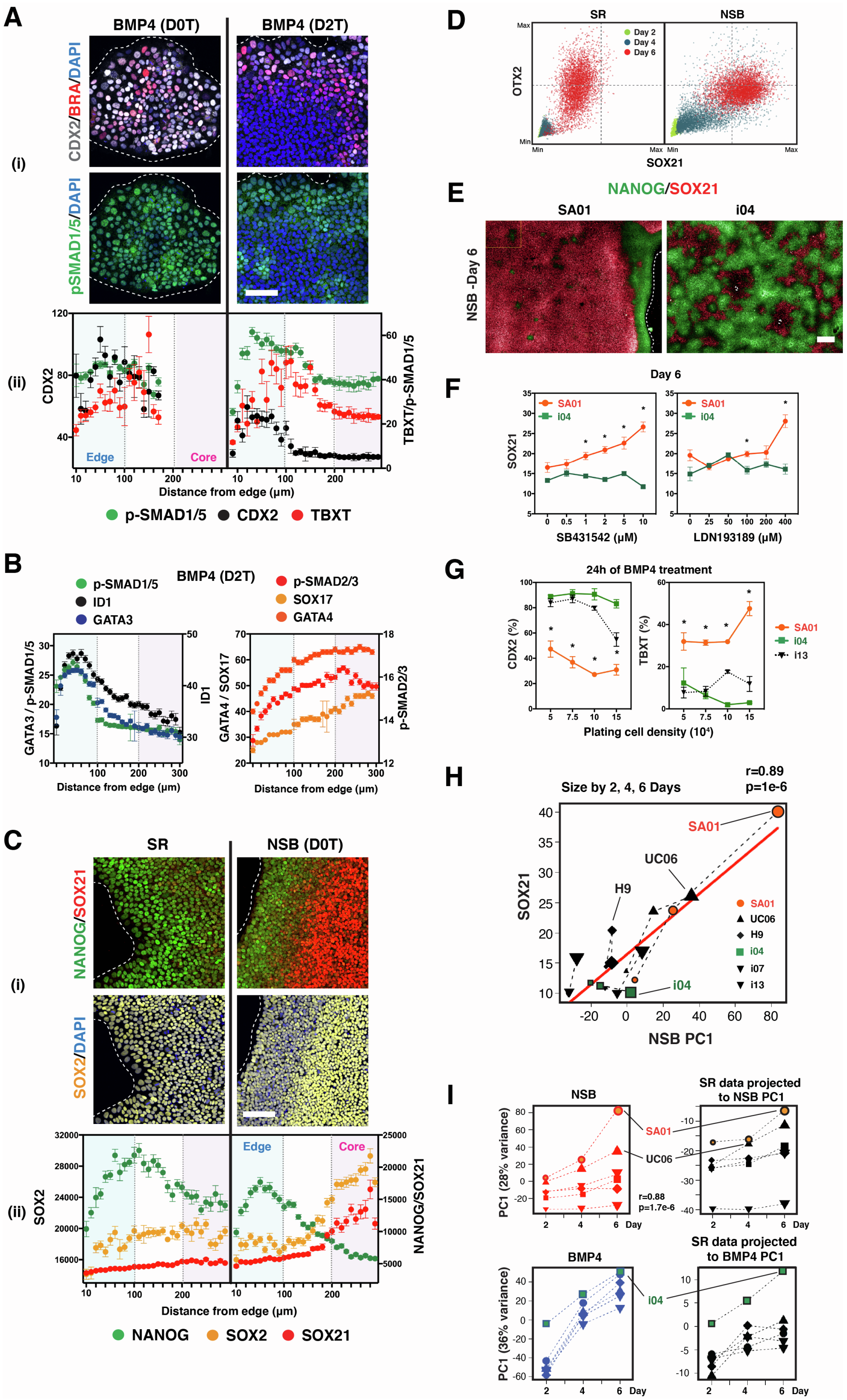

Figure S1. Cell line variation in the emergence of neural fate from pluripotency, related to Figure 1. (**A**) CDX2, TBXT, and pSMAD1/5 levels at 24 hours after BMP4 treatments (D0T: treated on day 0, D2T: treated on day 2). (i) Representative images. Scale bar, 100 μm. (ii) Expression levels plotted against distance from the edge. (**B**) Spatial expression of mesendoderm regulators at 24 hours after BMP4 treatment on day 2 (D2T). (**C**) Spatial expression of NANOG, SOX21, and SOX2 on day 4 in SR and NSB. (i) Representative images. Scale bar, 100 μm. (ii) Spatial expression levels. (**D**) Scatter plots showing single-cell levels of SOX21 and OTX2 expression in self-renewal (SR) and NSB conditions through culture times demonstrate a positive correlation between them during neuroectodermal differentiation. (**E**) SOX21 and NANOG expression on day 6 of NSB treatment in SA01 and i04 lines show a more efficient formation of the core zone in SA01 compared to i04. Scale bar, 200 μm. Dashed lines indicate the edge of colonies. (**F**) Dose-response curve of BMP/TGFβ signaling inhibitors LDN193189 and SB431542 on SOX21 induction on day 6 shows differential responses between SA01 and i04 lines. *, $p<0.05$ between lines. n=3 independent experiments. (**G**) Proportion of CDX2 and TBXT expressing cells in SA01 and i04 lines after 24 h of BMP4 treatment on day 0 (D0T) shows the cell line difference is not caused by initial cell plating density. *, $p<0.05$ between lines. n=3 independent experiments. (**H**) Scatter plot showing the correlation between SOX21 protein levels in NSB (Figure 1B) and NSB PC1. (**I**) Projections of SR data into PC1 of data from differentiation showing cell line-specific transcriptional lineage priming. PC1 of NSB and BMP4 data (left) and projection of SR data into the NSB and BMP4 PC1 (right).

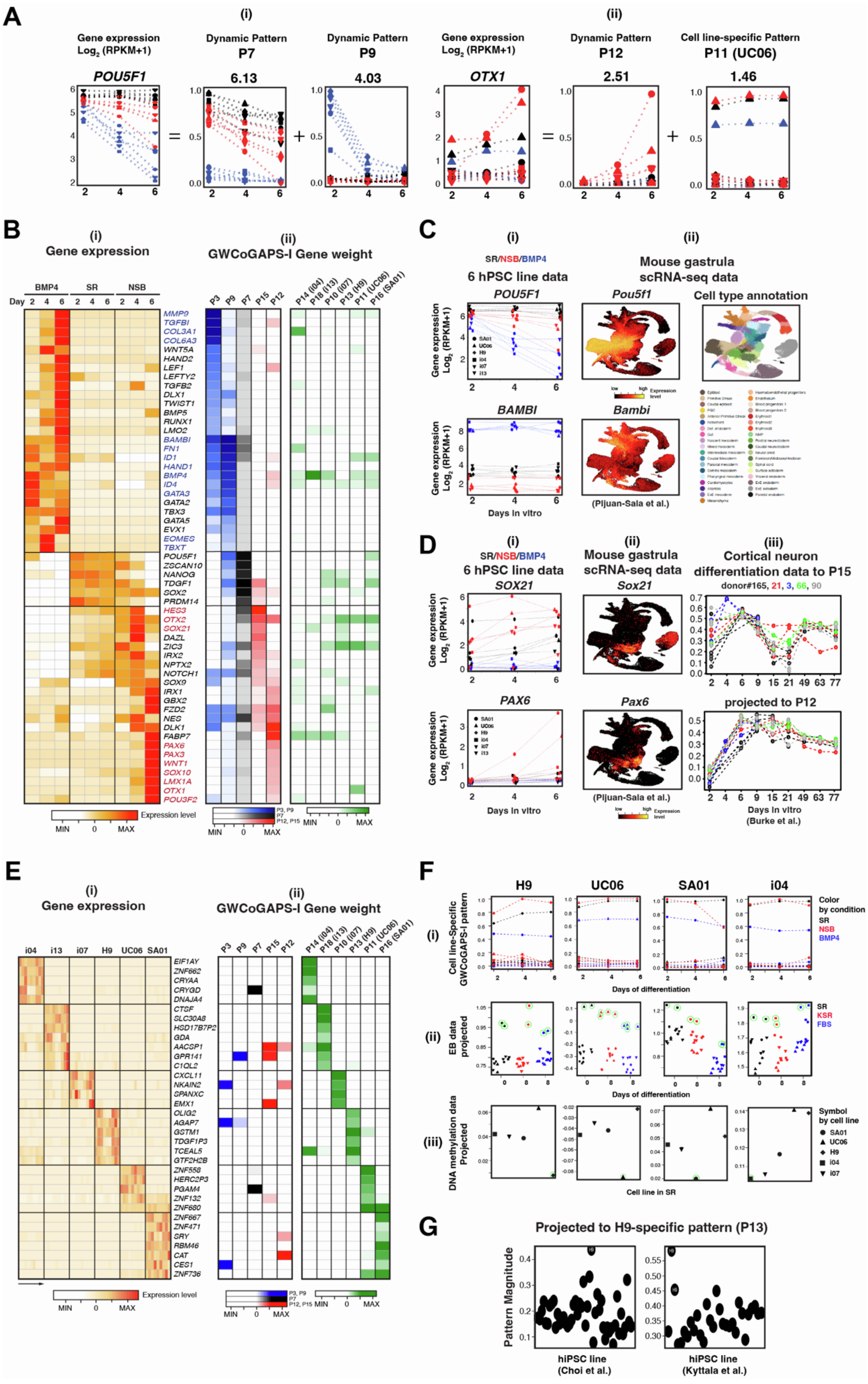
A
(i)
(ii)
Gene expression Log2 (RPKM+1)
Dynamic Pattern P7
Dynamic Pattern P9
Dynamic Pattern P12
Cell line-specific Pattern P11 (UC06)
POU5F1
OTX1
6.13
4.03
2.51
1.46
B
(i) Gene expression
(ii) GWCoGAPS-I Gene weight
BMP4
SR
NSB
Day
Expression level
MIN
MAX
C
(i)
(ii)
SR/NSB/BMP4
6 hPSC line data
Mouse gastrula scRNA-seq data
Pou5f1
Cell type annotation
BAMBI
Bambi
Gene expression Log2 (RPKM+1)
Days in vitro
(Pijuan-Sala et al.)
D
(iii)
Cortical neuron differentiation data to P15
SOX21
Sox21
PAX6
Pax6
projected to P12
(Burke et al.)
E
(i) Gene expression
(ii) GWCoGAPS-I Gene weight
i04
i13
i07
H9
UC06
SA01
F
Cell line-Specific GWCoGAPS-I pattern
Color by condition
SR
NSB
BMP4
Days of differentiation
EB data projected
KSR
FBS
DNA methylation data Projected
Symbol by cell line
Cell line in SR
G
Projected to H9-specific pattern (P13)
Pattern Magnitude
hiPSC line (Choi et al.)
hiPSC line (Kyttala et al.)

Figure S2. Decomposing dynamic and cell line-specific transcriptomic modules, related to Figure 2. (**A**) The use of each GWCoGAPS pattern across genes can be precisely defined by gene-specific amplitudes for all patterns (Table S1). Two examples of individual genes whose expression patterns are represented by the combination of multiple GWCoGAPS patterns are shown. (i) The complete expression of *POU5F1* is represented by two dynamic GWCoGAPS patterns P7 and P9. (ii) The complete expression of *OTX1* is represented by a dynamic GWCoGAPS pattern P12 and UC06 line-specific GWCoGAPS pattern P11. (**B**) Gene expression of pluripotency or differentiation regulators and their weights in dynamic and cell line-specific GWCoGAPS-I patterns. (i) Heatmap of gene expression. Gene expression levels were normalized across the row (gene) to show their expression dynamics in time and condition. (ii) Heatmap showing the gene weights. SR pattern: P7; NSB pattern: P12 and P15; BMP4 pattern: P9 and P3. The A values of the genes were normalized within each column (pattern) to show their representations in each dynamic or cell line-specific pattern. (**C**) GWCoGAPS-I pattern P7 represents the loss of pluripotency, and P3 represents the induction of mesendoderm regulators. (i) Expression of *POU5f1* and *BAMBI*, the top-ranked genes in P7 and P3, respectively, in 6 hPSC line data. (ii) Expression of *Pou5f1* and *Bambi* in mouse gastrula scRNA-seq data(Pijuan-Sala et al., 2019). (**D**) NSB patterns P15 and P12 represent distinct transition steps toward neural fates. (i) Expression of *SOX21* and *PAX6*, the top-ranked genes in P15 and P12, respectively, in 6 hPSC line data. (ii) Expression of *Sox21* and *Pax6* in mouse gastrula scRNA-seq data. (iii) Projection of cortical neuron differentiation data (Burke et al., 2020) from multiple hiPSC lines. (**E**) Expression of cell line-specific genes exhibiting the highest weights in each line-specific pattern and their weights in dynamic and 6 cell line-specific patterns. (i) Heatmap of gene expression. Gene expression levels were normalized across the row (gene) to show their expression across the time, condition, and cell lines. The arrow indicates the order from left to right: 2, 4, and 6 days of SR, BMP4, and NSB conditions. (ii) Heatmap showing the gene weights. The A values of the genes were normalized within each column (pattern) to show their representations in each dynamic or cell line-specific pattern. (**F**) Projections of microarray (Mallon et al., 2013) and DNA methylation data generated from multiple hPSC lines into the cell line-specific GWCoGAPS-I patterns demonstrate the stability of the cell line-specific transcriptional signatures within a cell line. (i) H9, UC06, SA01, and i04 line-specific GWCoGAPS-I patterns that define distinct transcriptional signatures from all other lines across time and condition. The corresponding cell line samples are marked in green circles. (ii) Projections of microarray dataset containing the same 6 lines under embryoid body (EB) differentiation conditions (SR for self-renewal in hESC medium plus FGF2, KSR for ectodermal differentiation, and FBS for mesendodermal differentiation) into each cell line-specific patterns discriminate the corresponding cell line samples from all other lines. (iii) Projections of DNA methylation data show that promoters of genes expressed specifically in each cell line are hypomethylated in the corresponding cell line. (**G**) Projection of multiple hPSC line data (Choi et al., 2015; Kyttala et al., 2016) into H9-specific pattern. H9 samples are circled in green.

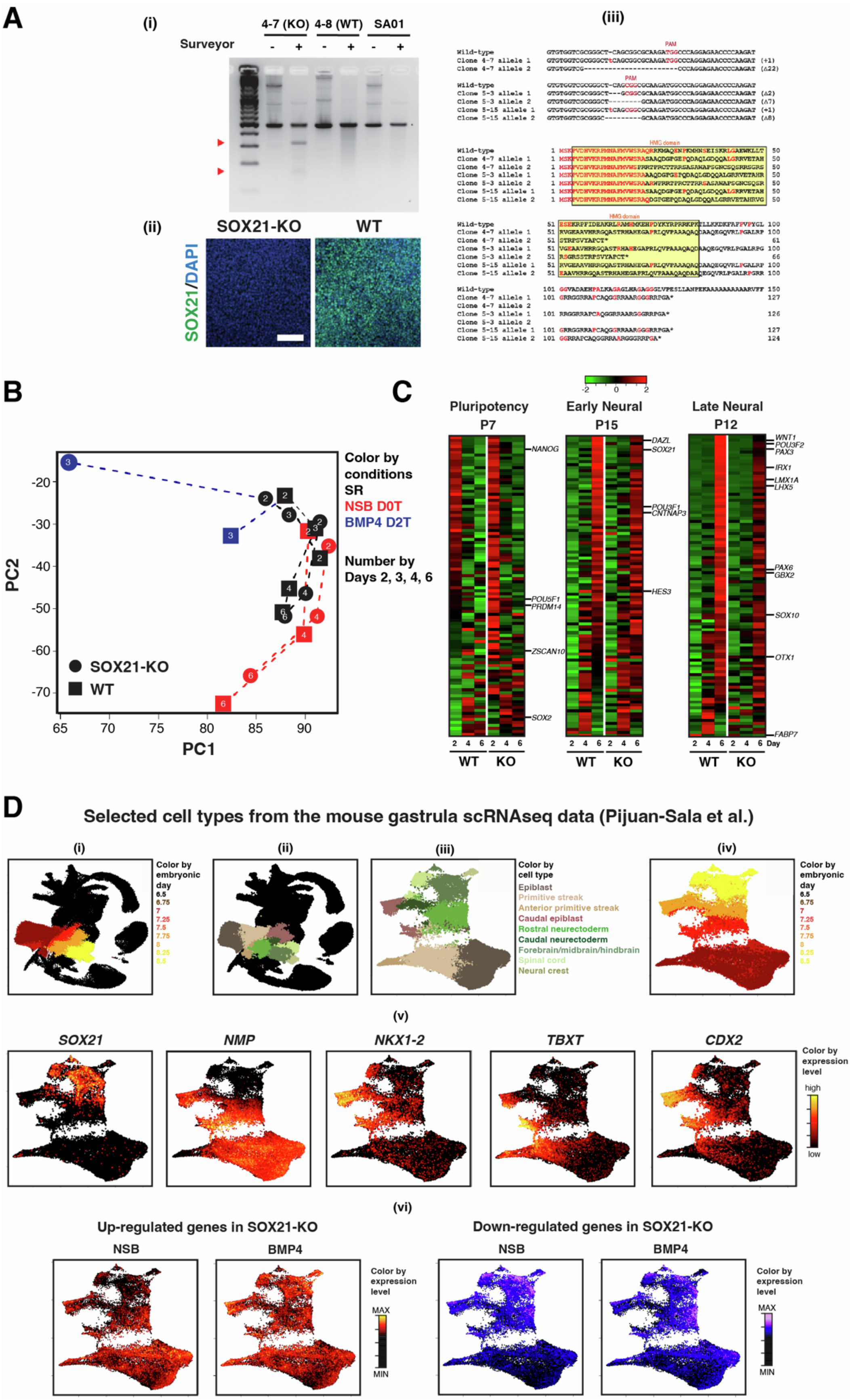
A
(i)
4-7 (KO) 4-8 (WT) SA01
Surveyor
(ii)
SOX21-KO
WT
SOX21/DAPI
(iii)
B
PC2
PC1
Color by conditions
SR
NSB D0T
BMP4 D2T
Number by Days 2, 3, 4, 6
SOX21-KO
WT
C
Pluripotency P7
Early Neural P15
Late Neural P12
NANOG
POU5F1
PRDM14
ZSCAN10
SOX2
DAZL
SOX21
POU3F1
CNTNAP3
HES3
WNT1
POU3F2
PAX3
IRX1
LMX1A
LHX5
PAX6
GBX2
SOX10
OTX1
FABP7
Day
WT KO
D
Selected cell types from the mouse gastrula scRNAseq data (Pijuan-Sala et al.)
(i)
(ii)
(iii)
(iv)
Color by embryonic day
Color by cell type
Epiblast
Primitive streak
Anterior primitive streak
Caudal epiblast
Rostral neurectoderm
Caudal neurectoderm
Forebrain/midbrain/hindbrain
Spinal cord
Neural crest
(v)
SOX21
NMP
NKX1-2
TBXT
CDX2
Color by expression level
high
low
(vi)
Up-regulated genes in SOX21-KO
Down-regulated genes in SOX21-KO
NSB
BMP4
MAX
MIN

Figure S3. SOX21 regulates early forebrain fate choice, related to Figure 3. (**A**) Establishment of SOX21-KO lines by CRISPR/Cas9 technology. All SOX21-KO SA01 hESC lines were screened using Surveyor and immunofluorescence assays and verified by DNA sequencing. (i) Analysis of SOX21-KO clones using Surveyor assay. The gel image shows modification at the SOX21 locus in a clone 4-7. Red arrowheads indicate expected fragment sizes for the SOX21 locus. (ii) Immunostaining of WT and SOX21-KO clones shows complete loss of SOX21 expression in clone 4-7. The cells were cultured in the presence of NSB for 6 days. Scale bar, 100 μm. (iii) Amino acid sequence of SOX21 alterations by CRISPR/Cas9 confirms knockout of SOX21. In three clones (clones 4-7, 5-3, and 5-15), frameshift mutation, premature stop codon mutation, or mutation that disrupts the HMG domain were confirmed in both *SOX21* alleles. These three clones were used for the functional assays shown in Figure 3. (**B**) Projection of WT and SOX21-KO line data into PC1 and PC2 of days 2, 4, and 6 data (Figure 1C) reveals delayed neuroectodermal differentiation in NSB and accelerated early mesendodermal differentiation under BMP4 D2T in SOX21-KO compared to WT. (**C**) Heatmaps showing the top 100 genes from P7, P15, and P12 (Table S1) in NSB. (**D**) Uniform manifold approximation and projection (UMAP) plots of selected epiblast and neural cell populations in mouse gastrula RNA-seq data (Pijuan-Sala *et al.*, 2019) showing *SOX21* and neuromesodermal precursor (NMP) gene expression. (i) A UMAP plot showing all cells with selected cell types colored by embryonic days. (ii) A UMAP plot showing all cells colored by cell type annotation with selected cell types (iii) A UMAP plot showing the selected cell types only and their cell type annotation. (iv) A UMAP plot showing the selected cell types colored by embryonic days. (v) Expression of *Sox21* and average expression of the neuromesodermal progenitor (NMP) genes in the epiblast and early neural lineage cells in mouse gastrula RNA-seq data. *Nkx1-2* expression delineates caudal epiblast cells showing the NMP gene signature, while *Tbxt* expression represents both the caudal epiblast cells and anterior primitive streak cells. Note the mutually exclusive expression between *Sox21* in the rostral neuroectoderm and *Cdx2* in caudal epiblast at developmental day 7.75, showing the early role of SOX21 in anterior-to-posterior axis patterning. (vi) A UMAP plot showing the projection of log fold changes of up- or down-regulated genes from the SOX21-KO RNA-seq data into the mouse gastrula RNA-seq data. Color scales range from the maximum to the minimum of the projected values within an analysis for each panel. The projected values are comparable within a panel but not between panels.

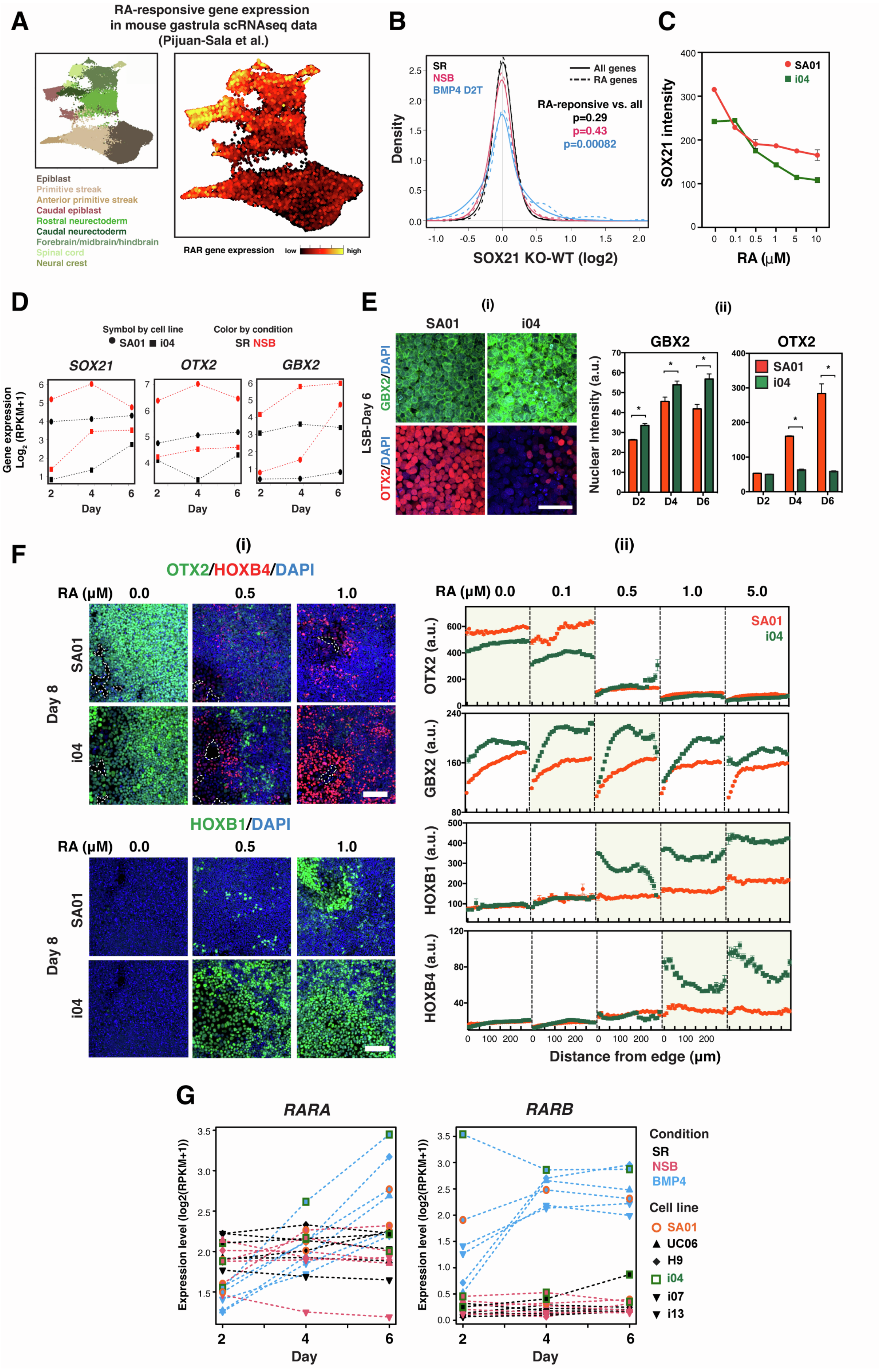
A
RA-responsive gene expression in mouse gastrula scRNAseq data (Pijuan-Sala et al.)
Epiblast
Primitive streak
Anterior primitive streak
Caudal epiblast
Rostral neurectoderm
Caudal neurectoderm
Forebrain/midbrain/hindbrain
Spinal cord
Neural crest
RAR gene expression
low
high
B
SR
NSB
BMP4 D2T
All genes
RA genes
RA-reponsive vs. all
p=0.29
p=0.43
p=0.00082
Density
SOX21 KO-WT (log2)
C
SA01
i04
SOX21 intensity
RA (μM)
D
Symbol by cell line
SA01
i04
Color by condition
SR NSB
SOX21
OTX2
GBX2
Gene expression
Log2 (RPKM+1)
Day
E
(i)
(ii)
SA01
i04
LSB-Day 6
GBX2/DAPI
OTX2/DAPI
GBX2
OTX2
Nuclear Intensity (a.u.)
D2
D4
D6
F
(i)
(ii)
OTX2/HOXB4/DAPI
RA (μM)
0.0
0.5
1.0
Day 8
SA01
i04
HOXB1/DAPI
RA (μM) 0.0
0.1
0.5
1.0
5.0
OTX2 (a.u.)
GBX2 (a.u.)
HOXB1 (a.u.)
HOXB4 (a.u.)
Distance from edge (μm)
G
RARA
RARB
Expression level (log2(RPKM+1))
Day
Condition
SR
NSB
BMP4
Cell line
SA01
UC06
H9
i04
i07
i13

Figure S4. Cell line variation in fore- vs. hind-brain fate bias, related to Figure 4. (**A**) A UMAP of the selected populations from the mouse gastrula scRNA-seq data colored by retinoic acid (RA)-responsive gene expression. (**B**) A density plot of all 252 RA-responsive genes (dotted lines) compared to all 21,022 genes (solid lines) in SR, NSB, and BMP4 D2T conditions of SOX21-KO RNA-seq data. (**C**) Dose-dependent reduction of SOX21 protein expression level in SA01 and i04 lines after RA treatment in neuroectoderm condition. n=3 technical replicates. (**D**) mRNA expression of *OTX2*, *GBX2*, and *SOX21* from the hiPSC RNA-seq data. (**E**) Differential GBX2 and OTX2 expression in SA01 and i04. (i) Representative images on day 6 in neuroectoderm differentiation conditions (LDN193189 and SB431542, LSB). Scale bar, 50 μm. (ii) OTX2 and GBX2 expression level in SR. *, Comparison between SA01 and i04 ($p<0.05$). n=3 technical replicates. (**F**) Dose-response analysis of RA shows differential posterization of neural precursors between SA01 and i04 lines. (i) Representative images show differential OTX2, HOXB4, and HOXB1 expression on day 8 of LSB+RA-induced differentiation between SA01 and i04 lines. Scale bar, 100 μm. Dashed lines indicate the edge of colonies. (ii) Dose-response analysis of RA on spatial expression of anterior-posterior neural regulators shows a sequential posteriorization from the core to edge zones and a cell line-specific RA response. Single-cell levels of OTX2, GBX2, HOXB1, and HOXB4 expression are measured on day 8 of LSB+RA-induced differentiation and plotted against the distance from the edge. In contrast, HOXB9 and HOXD9, markers of the posterior spinal cord and trunk neural crest, were not detected in RA-treated cells (data not shown). (**G**) Gene expression of *RARA* and *RARB* in 6 PSC lines across different conditions.

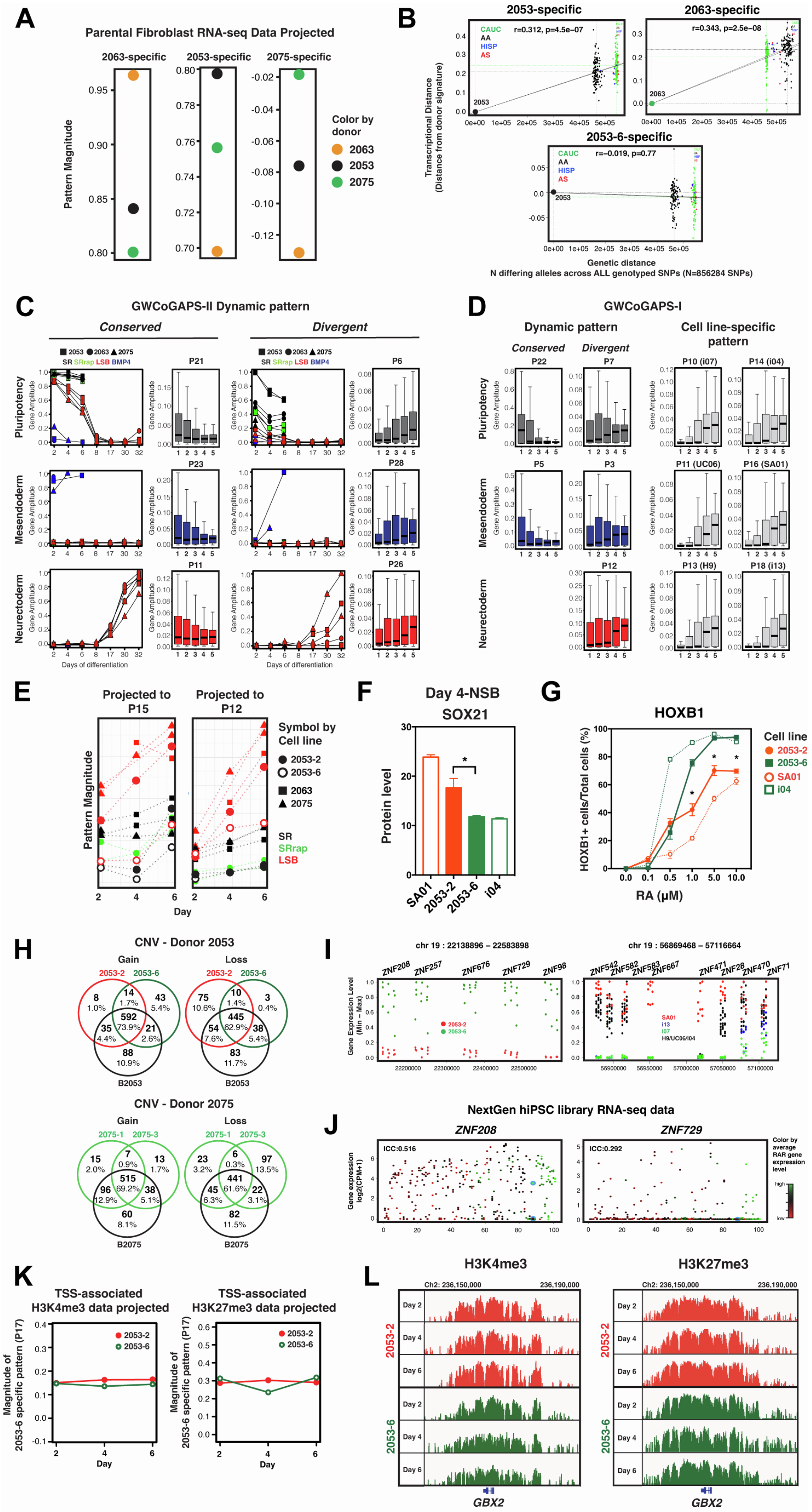
A
Parental Fibroblast RNA-seq Data Projected
2063-specific
2053-specific
2075-specific
Pattern Magnitude
Color by donor
2063
2053
2075
B
2053-specific
2063-specific
2053-6-specific
CAUC
AA
HISP
AS
r=0.312, p=4.5e−07
r=0.343, p=2.5e−08
r=−0.019, p=0.77
Transcriptional Distance (Distance from donor signature)
Genetic distance
N differing alleles across ALL genotyped SNPs (N=856284 SNPs)
C
GWCoGAPS-II Dynamic pattern
Conserved
Divergent
2053 2063 2075
SR SRrap LSB BMP4
Pluripotency
Mesendoderm
Neurectoderm
Gene Amplitude
Days of differentiation
P21
P6
P23
P28
P11
P26
D
GWCoGAPS-I
Dynamic pattern
Cell line-specific pattern
Conserved
Divergent
P22
P7
P10 (i07)
P14 (i04)
P5
P3
P11 (UC06)
P16 (SA01)
P12
P13 (H9)
P18 (i13)
E
Projected to P15
Projected to P12
Pattern Magnitude
Day
Symbol by Cell line
2053-2
2053-6
2063
2075
SR
SRrap
LSB
F
Day 4-NSB
SOX21
Protein level
SA01
2053-2
2053-6
i04
G
HOXB1
HOXB1+ cells/Total cells (%)
RA (μM)
Cell line
2053-2
2053-6
SA01
i04
H
CNV - Donor 2053
Gain
Loss
2053-2
2053-6
B2053
CNV - Donor 2075
2075-1
2075-3
B2075
I
chr 19 : 22138896 – 22583898
chr 19 : 56869468 – 57116664
Gene Expression Level (Min – Max)
ZNF208
ZNF257
ZNF676
ZNF729
ZNF98
ZNF542
ZNF582
ZNF583
ZNF667
ZNF471
ZNF28
ZNF470
ZNF71
SA01
i13
i07
H9/UC06/i04
J
NextGen hiPSC library RNA-seq data
ZNF208
ZNF729
ICC:0.516
ICC:0.292
Gene expression log2(CPM+1)
Color by average RAR gene expression level
high
low
K
TSS-associated H3K4me3 data projected
TSS-associated H3K27me3 data projected
Magnitude of 2053-6 specific pattern (P17)
2053-2
2053-6
Day
L
H3K4me3
H3K27me3
Ch2: 236,150,000
236,190,000
Day 2
Day 4
Day 6
2053-2
2053-6
GBX2

Figure S5. Genetic and epigenetic elements contribute to donor- and line-specific transcriptomic signatures, related to Figure 5. (**A**) Projection of parental fibroblast RNA-seq data into donor-specific patterns shows donor-specific transcriptomic signatures are highest in the parental fibroblasts of the corresponding donor. (**B**) Scatter plots showing the association between genetic similarity and the strength of donor-specific transcriptomic signatures across pair-wise comparisons with other donors. Each point is a comparison of the highlighted donor to another donor. Genetic distance between donors was quantified as the number of differing alleles across all genotyped single nucleotide polymorphisms (SNPs on the X-axis). The transcriptional distance was quantified as the strength of the donor-specific transcriptomic signature in other donors' tissue (Y-axis; same data depicted in the Y-axis of Figures 5Aii and 5Cii, centered such that the source donor has a value of zero). Pearson's correlation and p-values were calculated by omitting the donor data point at the origin. Solid black lines pass through the origin and each ethnicity's average Y-value, indicated as horizontal dashed lines. The ethnicity of donors is indicated by color: CAUC, Caucasian; AA, African American; HISP, Hispanic; AS, Asian. (**C**) Contribution of genes of 5 evolutionary eras (1, Ancient; 2, Animal; 3, Chordate; 4, Mammal; 5, Primate) to the conserved or divergent dynamic patterns of GWCoGAPS-II analysis. Dynamic patterns conserved across cell lines show high gene amplitudes in ancient genes, while dynamic patterns divergent across cell lines show higher gene amplitudes in newer genes. Comparison of the distribution of era 1 gene amplitudes to era 5 gene amplitudes by Wilcoxon rank sum test: $p>1e-16$ for all patterns shown. (**D**) Contribution of genes of 5 evolutionary eras to the conserved or divergent dynamic and cell line-specific patterns of GWCoGAPS-I analysis ($p<1e-16$ for all patterns shown). (**E**) Projection of RNA-seq data into the neural patterns P15 and P12. (**F**) Differential SOX21 levels in SA01, i04, and two 2053 lines. *, Comparison between 2053-2 and 2053-6 ($p<0.05$). n=3 independent experiments. (**G**) Two replicate lines from donor 2053 show differential responsiveness to RA. Number of HOXB1 expressing cells on day 8 in response to varying doses of RA in SA01, i04, and two 2053 lines. *, Comparison between 2053-2 and 2053-6 ($p<0.05$). n=3 independent experiments. (**H**) The Venn diagrams showing the copy number variants (CNVs) gained and lost in the two replicate hiPSC lines and the mature post-mortem brain tissue of donors 2053 and 2075 with respect to the reference genome. In all cases, the vast majority of differences from the reference genome are the same across both hiPSC lines and the brain tissues derived from each donor (B2053 and B2075), indicating little change in genomic DNA. A similar analysis for single nucleotide variants (SNVs) showed similar results (data not shown). (**I**) Differential expression of the clustered ZNF genes across the discordant hiPSC lines 2053-2 and 2053-6 (left) and across the 6 hPSC lines (right). Notably, differential expression of KRAB-ZNF genes was also observed between lines derived from the same donor, while genome sequencing revealed no detectable CNVs in these genes. Plots show the expression of each gene from its minimum to maximum in pluripotency, NSB/LSB, and BMP4 conditions for all lines. (**J**) Expression of *ZNF208* and *ZNF729*, two of the genes expressed specifically in line 2053-6 (Figure S6I) in the NextGen hiPSC data. Replicate lines from individual donors are displayed as points along a single X-axis value. Donors are ordered by their rank in the first principal component of this data and colored by RA-responsive gene expression. *ZNF208* shows high expression in most lines, with a minority of lines showing very low expression. In contrast, *ZNF729* showed no reads in most lines, and a few lines showed significant expression. While quite distinct from one another, the expression patterns of both genes are consistent with epigenetic regulation, i.e., divergent expression levels across multiple lines derived from the same donor. (**K**) Projection of H3K4me3 and H3K27me3 ChIP-seq data from lines 2053-2 and 2053-6 in SR into the 2053-6 line-specific pattern. Ranges of the Y axis of ChIP-seq projections represent the biggest variation observed within all the patterns for each histone modification as we have done with H3K9me3-projection in Figure 5E. (**L**) H3K4me3 and H3K27me3 ChIP-seq

data at the GBX2 locus in lines 2053-2 and 2053-6 in SR condition.

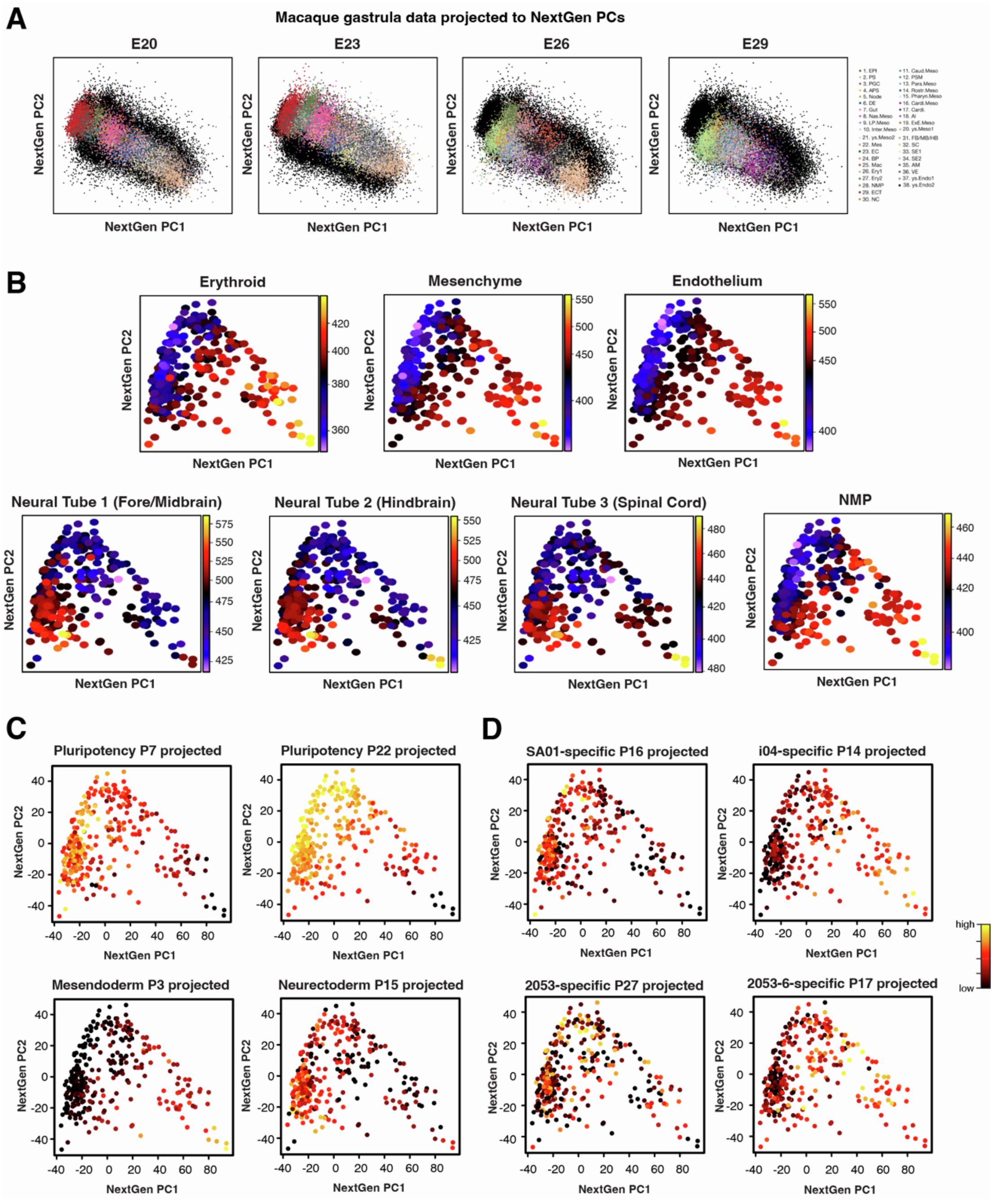

Figure S6. Early developmental bias and retinoic acid signaling define hPSC variation in the wider human population, related to Figure 6. (**A**) Projection of Carnegie stage CS8-11 cynomolgus macaque gastrula data (Zhai et al., 2022) into NextGen PC1 and PC2. (**B**) Average expression of lineage-specific gene sets defined by the transcriptomic signature of CS10 human embryo (Zeng et al., 2023) in the NextGen PSC lines. (**C**) Projection of GWCoGAPs-I dynamic patterns into PC1 and PC2 of NextGen hPSC RNA-seq data(Carcamo-Orive et al., 2017). (**D**) Projection of GWCoGAPs-I and -II cell line-specific patterns into PC1 and PC2 of NextGen hPSC RNA-seq data

Supplemental Table Legends (separate files)

Supplemental Table S1. Information on hPSC lines, RNA-seq data, and individual gene amplitudes for GWCoGAPS-I patterns, related to Figure 2.
Sheet 1: information on hPSC lines used, Sheet 2: summary of experimental times and conditions for RNA-seq data generation, Sheet 3: mapping and annotation details of RNA-seq, Sheet 4: individual gene amplitudes for GWCoGAPS-I patterns, Sheet 5: list of top 100 genes in 22 GWCoGAPS-I patterns,

Supplemental Table S2. Individual gene amplitudes for GWCoGAPS-II and genes ranked by their contribution to the line- and donor-specific patterns, related to Figure 5.
Submitting the top 250 genes from the line- and donor-specific patterns to the Enrichr webtool (https://maayanlab.cloud/Enrichr/enrich?dataset=3f14ebb0f1889f3a5acb6d940326bebb; https://maayanlab.cloud/Enrichr/enrich?dataset=cb12b784682d4aed17f5b17d6cdbee3b) revealed enrichment in 1) Co-expression with many KRAB-ZNF genes (p=7.8e-9 to p=5.8e-6) from the ARCHS4 database, 2) H3K9me3 (p=1.8e-19 to p=6.4e-7) and SETDB1 (p=9.7e-8) ChIP-seq peaks from the ENCODE Histone modifications and TF ChIP-seq databases, and 3) TRIM28/KAP1 protein-protein interactions from the BioPlex database (p=6.8e-6). These enrichments represent a canonical mechanism of transcriptional repression: KRAB-ZNF genes bind repetitive genomic DNA derived from TEs and TRIM28/KAP1, recruiting SETDB1 to deposit the H3K9me3. We also found enrichment of protocadherin (PCDH) genes among the GWCoGAPS gene amplitudes in line-and donor-specific patterns (p=2e-17 and p=0.00046, in GWCoGAPS-I and –II, respectively). Y chromosome genes in these lists serve as a control positive in that their expression patterns are described primarily as a combination of the male line-specific patterns. Genes in line- and donor-specific expression patterns can be explored in our NeMO Analytics portal, where examples of both epigenetic (e.g., ZNF genes in Figures S5I and S5J) and genetic (e.g., NOMO3) regulation can be observed in our hPSC lines and the NextGen Consortium lines: https://nemoanalytics.org/p?l=Kim2024&g=NOMO3.

Supplemental Table S3. **Normalized H3K9me3 levels in 2053-2 and 2053-6 lines in self-renewal**, related to Figure 5. In particular, *GBX2* and other hindbrain fate genes IRX1/2/4, ZIC1, and OLIG3 were highly represented in the 2053-6 line-specific signature and showed higher H3K9me3 levels in 2053-2. Although HOX genes were not expressed in SR condition, 28 HOX genes were among the top 100 with increased H3K9me3 levels in line 2053-2.

Supplemental Table S4. Gene sets used to explore pluripotency states and anterior-to-posterior bias in NextGen hiPSC library dataset, related to Figure 6.
Sheet 1: Gene weights of PC3 of mouse mid-streak embryo data. Sheet 2: Values of retinoic acid response genes in PC1 and PC2 of NextGen RNA-seq data. Sheet 3: Gene weights of PC1 of mouse ESC and EpiSC data.

Supplemental experimental procedures

hPSC culture and differentiation

For posterior neural differentiation, cells were cultured with Aggrewell medium (05893, Stem Cell Technology) for 2 days after ROCK inhibitor removal and then cultured with N2B27 medium supplemented with LDN193189 (100 nM, 04-0074, Stemgent) and SB431542 (2 μM, S4317, Sigma-Aldrich) for another 4 days. Retinoic acid (RA, R2625, Sigma-Aldrich) was added on day 4 to induce posterior neuroectoderm differentiation. For further differentiation to hindbrain-derived neurons, cells were cultured with neurobasal medium (21103-049, Life Technologies) supplemented with bovine Insulin (25 μg/ml, I6634, Sigma-Aldrich), B27 (17504-044, Life Technologies), human BDNF (10 ng/ml, 248-BD, R&D Systems) and human NT-3 (10 ng/ml, 267-N3, R&D Systems) for another 20 days. For inhibition of RA signaling, selective RARα antagonist BMS195614 (5 μM, 3660, Tocris) and RARβ antagonist LE135 (5 μM, 2021, Tocris) were additionally added to the neuroectoderm condition (LDN193189, 100 nM and SB431542, 2 μM) for 4 days.

Generation of hiPSC lines

Previously reported hiPSC lines i04, i07, and i13 (NIH-i4, NIH-i7, NIH-i13) are detailed in(Mallon *et al.*, 2013). The hiPSC lines (2075, 2053, and 2063) were reprogrammed using synthetic mRNAs (Oct4, Klf4, Sox2, c-Myc, Lin-28; OSKML), mRNA reprogramming kit (00-0071, Stemgent), and microRNA Booster kit (00-0073, Stemgent). Human fibroblasts (Donor 2075, 2053, and 2063) were seeded at 5 X 10^3 cells/cm^2 in a Matrigel-coated plate and cultured with DMEM medium supplemented with 10% FBS (Life Technologies) and 2 mM L-glutamine. After 24 hours (day 1), the medium was changed to Pluriton human NUFF-conditioned media with 300 ng/ml B18R protein. On days 1 and 5, the microRNA booster kit was used with the StemFect RNA transfection reagent kit (00-0069, Stemgent) to enhance reprogramming. On days 2-12, the OSKML RNAs were transfected. The mRNA reprogramming process was performed at 37°C in a 5% O_2 and CO_2 incubator. These lines were validated for pluripotency marker expression, trilineage differentiation potential, and normal karyotype, with karyotyping performed every 10 passages.

Generation of CRISPR/Cas9 mediated SOX21-KO hESC line

SOX21-KO hESC lines were generated using CRISPR/Cas9. SOX21-specific gRNAs were designed using the Optimized CRISPR Design-MIT for Sox21NHEJ4 (http://crispr.mit.edu/) and CHOPCHOP for Sox21NHEJ5 (https://chopchop.rc.fas.harvard.edu/). Oligonucleotides (CACCGCGGGCTCAGCGGCGCAAGA –top for Sox21NHEJ4; AAACTCTTGCGCCGCTGAGCCCGC –bottom for Sox21NHEJ4; CACCGGGTGTGGTCGCGGGCTCAG –top for Sox21NHEJ5; AAACCTGAGCCCGCGACCACACCC –bottom for Sox21NHEJ5) were cloned into pSpCas9(BB)-2A-Puro (px459; Addgene), producing plasmid pX459-Sox21NHEJ4 and pX459-Sox21NHEJ5, synthesized by Integrated DNA Technologies. SA01 hESCs were transfected with 2.5 μg of either plasmid using DNA-In Stem (MTI-Global stem, gifted from Dr. Jessee). Transfected cells were dissociated and plated into a 10 cm culture dish. After 48 hours of 0.5 μg/ml puromycin selection, hESC colonies were maintained for 10 days. Individual colonies were isolated and clonally expanded. Genomic DNA was isolated from each clonal line using Wizard Genomic DNA Purification Kit (Promega). The genomic region surrounding the CRISPR target site for SOX21 was amplified by PCR (KOD Xtreme Hot Start DNA Polymerase; EMD Millipore), and products were treated with SURVEYOR nuclease (SURVEYOR Mutation Detection Kit, Transgenomic) to detect CRISPR/Cas9-induced indel mutations. The PCR products were cloned

into pGEM®-T Easy Vector (Promega) and sequenced to confirm the genotypes. Knockout validation of SOX21 protein was performed by immunostaining.

Immunofluorescence

Cells were fixed with 4% paraformaldehyde for 10 min and permeabilized for 40 min using 0.1% Triton X-100 (Sigma-Aldrich) in PBS. Subsequently, cells were blocked with 10% donkey serum (Sigma-Aldrich) and incubated with primary antibodies overnight. Following primary antibodies and dilutions were used: TBXT (AF2085, R&D, 1:500), CDX2 (AM392, Biogenex), EOMES (ab23345, Abcam, 1:400), GATA3 (MAB6330, R&D, 1:200), GATA4 (AF2606, R&D, 1:400), GBX2 (AF4638, R&D, 1:200), HOXB1 (AF6318, R&D, 1:200), HOXB4 (ab133621, Abcam, 1:400), HOXB9 (ab66765, Abcam, 1:400), ID1 (AF4377, R&D, 1:200), ISLET1 (AF1837, R&D, 1:200), NANOG (AF1997, R&D, 1:200; Reprocell 1:200), OCT4A (MAB17591, R&D, 1:200), OLIG2 (AF2418, R&D, 1:200), OTX2 (AF1979, R&D, 1:200), PAX6 (PRB-278P, BioLegend, 1:500; AF8150, R&D, 1:200), PHOX2B (AF4940, R&D, 1:200), p-SMAD1/5 (9516, Cell Signaling Technology, 1:200), p-SMAD2/3 (8828, Cell Signaling Technology, 1:200), and TUJ1 (PRB-435P, BioLegend, 1:1000), SOX1 (AF3369, R&D, 1:400), SOX17 (AF1924, R&D, 1:500), SOX2 (AF2018, MAB2018, R&D, 1:200), SOX21 (AF3538, R&D, 1:200), SOX3 (GT15119, Neuromics, 1:200), and TUJ1 (MAB1195, R&D, 1:400). Secondary antibody incubation was performed with Alexa flour conjugated antibodies at dilution of 1:400 (Life Technologies). For direct immunostaining, primary antibodies were conjugated using Alexa fluor monoclonal antibody labeling kits (A20181, A20184, A20186, Life Technologies). Nuclei were counterstained with DAPI (Life Technologies).

High-content spatial analysis

Images were acquired with the Operetta (Perkin Elmer), analyzed in batch mode with custom building blocks on a Columbus server (Perkin Elmer), and visualized with Spotfire (Perkin Elmer). Spatial analysis of hPSC epithelium ('distance from the edge' measurement) was achieved using a custom Acapella script (Perkin Elmer) run in Columbus with the following commands; 1) stitch a montage from 3x3 user-defined contiguous overlapping fields captured with the 20x objective, 2) segment and binarize DAPI signal from individual nuclei to create nuclear objects, 3) segment and binarize DAPI signal from the cytoplasm surrounding each nucleus to create cytoplasmic objects. hPSCs show strong blue fluorescence arising from sequestration of retinyl esters in cytoplasmic lipid bodies(Muthusamy et al., 2014), 4) dilate nuclear objects to eliminate gaps between neighboring objects, 5) create super objects by filling holes containing less than 30 pixels, 6) segment super objects, 7) create a perimeter line at the edge of each super object, 8) calculate the minimum distance between the centroid of each nucleus and the closest super object perimeter, 9) report fluorescence signal from nucleus and cytoplasm for each object. For each cell, this script reports nuclear and cytoplasmic signals for all channels and a single measure of minimum distance to the closest perimeter of the epithelium. Using data visualization in Spotfire, median fluorescence signals from all cells were plotted corresponding to the distance from an edge of epithelium.

RNA-seq library preparation

Total RNA was extracted using the mirVana kit (Ambion). RNA quality control was performed using the Agilent 2100 Bioanalyzer System. RNA-seq libraries were constructed using Illumina mRNA sequencing sample Prep Kit (for Poly-A libraries) or TruSeq Stranded Total RNA RiboZero sample Prep Kit (for strand-specific libraries). Briefly, poly-A-containing mRNA molecules were purified or ribosomal RNAs were removed using RiboZero beads from ~ 800 ng DNase-treated total RNA. Following purification, the resulting RNA was fragmented into small pieces using

divalent cations under elevated temperature at 94°C for 2 min. The range of the fragment length was 130-290 bp, with a median length of 185 bp. Reverse transcriptase and random primers were used to copy the cleaved RNA fragments into first-strand cDNA. The second-strand cDNA was synthesized using DNA Polymerase I and RNase H. These cDNA fragments went through an end repair process using T4 DNA polymerase, T4 PNK, and Klenow DNA polymerase, the addition of a single 'A' base using Klenow exo (3' to 5' exo minus) and the ligation of Illumina PE adapters using T4 DNA Ligase. An index was inserted into Illumina adapters so that multiple samples could be sequenced in one lane of an 8-lane flow cell if necessary. The concentration of RNA was measured by Qubit (Life Technologies). The quality of the RNA-seq library was measured by LabChipGX (Caliper) using HT DNA 1K/12K/HiSens Labchip. The final cDNA libraries were sequenced using HiSeq 2000 (for Poly-A library preparation) or HiSeq 3000 (for RiboZero library preparation) for high-throughput DNA sequencing.

RNA-seq data processing

After the sequencing, the Illumina Real Time Analysis (RTA) module was used to perform image analysis and base calling, and the BCL Converter (CASAVA v1.8.2) was used to generate FASTQ files, which contain the sequence reads. The sequencing depth was over 80 million (40 million paired-end) mappable sequencing reads (Table S1). Read-level Q/C was performed by FastQC (v0.10.1). Pair-end reads of cDNA sequences were aligned back to the human genome (UCSC hg19 from Illumina iGenome) by the spliced read mapper TopHat (v2.0.4) with default option with "--mate-innder-dist 160" based on known transcripts of Ensembl Build GRCh37.75. For stranded RiboZero samples, TopHat used "--library-type fr-firststrand" option. The alignment statistics and Q/C were achieved by samtools (v0.1.18) and RSeQC (v2.3.5) to calculate quality control metrics on the resulting aligned reads, which provides useful information on mappability, uniformity of gene body coverage, insert length distributions and junction annotation, respectively. To achieve a gene-level expression profile, the properly paired and mapped reads were achieved by "samtools sort –n" option, and these reads were counted by htseq-count v0.5.3 (with intersection-strict mode and stranded option for RiboZero samples) according to gene annotation (Illumina iGenome), and RPKM was calculated. This provided 23,368 gene-level expression profiles.

Statistics for SOX21 protein level

To determine the effect of a cell line of origin on nuclear SOX21 protein levels in Figure 1B, we used a mixed model comparing the mean expression levels across cell lines while accounting for the correlation of expression levels within replicate experiments (a total of 5 independent experiments were conducted) with a random intercept, implemented in R using the lme4 library and the lmer() function: expression~ as.factor (line)*condition*day+(1| replicate). To test the effect of the line of origin, this model was compared to a second model with no line effect using anova().

Bioinformatic analyses

Principle component analysis was done using the prcomp() function in R. Agglomerative hierarchical clustering of genes using gene-level RPKM from RNA-seq data was performed using hclust() and cutree() with correlational distance (dist=1-r) in R. GWCoGAPS was run using default parameters as previously described(Fertig et al., 2012; Fertig et al., 2014; Fertig and Favorov, 2010; Stein-O'Brien et al., 2017), for a range of k patterns (k=22 selected) and uncertainty as 10% of the data. Briefly, whole transcriptomic data was parallelized into seven sets. GWCoGAPS decomposes a matrix of experimental observations, **D**—here, log2 RNA-seq RPKMs—with genes as rows and samples as columns, into two matrices, by the following equation.

$$\mathbf{D} \sim N(\mathbf{AP},\Sigma)$$

Where, **A** is the amplitude matrix indicating the strength of involvement of a given gene in each pattern, and **P** is the pattern matrix defining relationships (i.e., patterns) between samples. N and Σ are functions of each element of **AP** and represent the Normal distribution and the standard deviation, respectively. Projection of principal components and GWCoGAPS gene weights defines patterns of relationships between samples in new data associated with the gene expression signatures of the patterns from the primary data. These were achieved using the default projectR function in the projectR package as previously described(Fertig *et al.*, 2014). Enrichment was calculated via either the calcCoGAPSStat function in the CoGAPS Bioconductor package or the geneSetTest function in the limma Bioconductor package in R. ANOVAs were used to assess the association of each GWCoGAPS pattern with treatment, time, and cell line of origin, using lm() and summary() in R: lm(pattern~treatment*day+line). No filtering was applied for the NMF decomposition. All genes in the dataset were used in the GWCoGAPS analysis. This choice was made for multiple reasons. First, the RNA-seq data are from bulk in vitro cell samples that yielded ample high-quality starting RNAs sequenced at >79 M reads per sample (mean=127 M). The signal-to-noise ratio of this deeply sequenced bulk data is very high, especially compared to the currently popular scRNA-seq, which often requires heavy filtering of lowly expressed genes. Second, the GWCoGAPS NMF algorithm can effectively extract patterns from an extremely broad range of gene expression levels(Fertig *et al.*, 2014; Stein-O'Brien *et al.*, 2017; Stein-O'Brien et al., 2019).

Gene age estimation

Gene age was estimated by phylostratigraphy that uses protein sequence similarity scored by BLASTP to find the minimal evolutionary age of protein-coding genes(Domazet-Loso et al., 2007; Weber et al., 2020). For each protein, the National Center for Biotechnology Information (NCBI) nonredundant database was used to find the most distant species in which a sufficiently similar protein sequence exists. We estimated the minimal evolutionary age of a gene as the age of the ancestor of the query species, human in this study, and the most distant species harboring a sufficiently similar sequence. To find the most distant species, we used the NCBI taxonomy for every species and estimated the timing of lineage divergence events with TimeTree(Kumar et al., 2017). As in other studies, we used the e-value threshold of 10^{-3} to detect sequence similarity by BLASTP(Neme and Tautz, 2016; Vakirlis et al., 2020; Weber *et al.*, 2020). For all human protein sequences, we filtered the sequences for a minimal length of 40 amino acids and a maximal length of 4,000 amino acids and kept only one protein isoform per gene (the longest and evolutionary oldest). We counted the number of genes in each phylostratum (PS), from the most ancient (PS 1, Cellular organisms) to the most recent (PS 31, *Homo sapiens*). We aggregated gene counts from individual phylostrata into five broad evolutionary eras: Ancient (PS 1 to 3, Cellular organisms to Opisthokonta; 4290 to 2101 millions of years ago (MYA)); Animal (PS 4 to 7, Metazoa to Deuterostomia, 2101 to 747 MYA); Chordate (PS 8 to 17, Chordate to Amniota, 747 to 320 MYA); Mammal (PS 18 to 22, Mammalia to Euarchontoglires, 320 to 91 MYA); Primate (PS 23 to 31, Primates to *Homo sapiens*, 91 Mya to present).

ChIP-seq

The cells were crosslinked in 1% formaldehyde for 10 min at room temperature with constant agitation, followed by quenching with 125 mM glycine for 5 min. Nuclei were collected on days 2, 4, and 6 in self-renewal. Chromatin was fragmented with micrococcal nuclease (MNase) until the majority of DNA was in the range of 200-700 base pairs. Chromatin was incubated with an H3K9me3 antibody (13969, Cell signaling) at 4°C overnight, with constant agitation. Antibody-bound chromatin was immunoprecipitated with Protein G Dynabeads for 1 hour at 4°C, with

constant agitation. Protein-DNA complexes were eluted from Dynabeads, and the DNA was reverse crosslinked with 0.3 M NaCl with 4 h incubation at 65°C. Free DNA was purified with QIAquick Gel Extraction Kit (28706X4, Qiagen). ChIP-seq libraries were generated using TruSeq ChIP Library Preparation Kit (Illumina, IP-202-1012) and the libraries were sequenced on Illumina HiSeq 3000. ChIP-seq raw reads were aligned to the human genome assembly hg19 using Burrows-Wheeler Aligner (BWA), and peak calling was done using MACS2.1.1. To understand H3K9me3 occupancy in promotors, ChIP-seq reads within +/-3kb of TSS were counted using bedtools.

Supplemental References

Burke, E.E., Chenoweth, J.G., Shin, J.H., Collado-Torres, L., Kim, S.K., Micali, N., Wang, Y., Colantuoni, C., Straub, R.E., Hoeppner, D.J., et al. (2020). Dissecting transcriptomic signatures of neuronal differentiation and maturation using iPSCs. Nature communications *11*, 462. 10.1038/s41467-019-14266-z.

Carcamo-Orive, I., Hoffman, G.E., Cundiff, P., Beckmann, N.D., D'Souza, S.L., Knowles, J.W., Patel, A., Papatsenko, D., Abbasi, F., Reaven, G.M., et al. (2017). Analysis of Transcriptional Variability in a Large Human iPSC Library Reveals Genetic and Non-genetic Determinants of Heterogeneity. Cell stem cell *20*, 518-532.e519. 10.1016/j.stem.2016.11.005.

Choi, J., Lee, S., Mallard, W., Clement, K., Tagliazucchi, G.M., Lim, H., Choi, I.Y., Ferrari, F., Tsankov, A.M., Pop, R., et al. (2015). A comparison of genetically matched cell lines reveals the equivalence of human iPSCs and ESCs. Nature biotechnology *33*, 1173-1181. 10.1038/nbt.3388.

Domazet-Loso, T., Brajkovic, J., and Tautz, D. (2007). A phylostratigraphy approach to uncover the genomic history of major adaptations in metazoan lineages. Trends in genetics : TIG *23*, 533-539. 10.1016/j.tig.2007.08.014.

Fertig, E.J., Ren, Q., Cheng, H., Hatakeyama, H., Dicker, A.P., Rodeck, U., Considine, M., Ochs, M.C., and Chung, C.H. (2012). Gene expression signatures modulated by epidermal growth factor receptor activation and their relationship to cetuximab resistance in head and neck squamous cell carcinoma. BMC genomics *13*.

Fertig, E.J., Stein-O'Brien, G., Jaffe, A., and Colantuoni, C. (2014). Pattern identification in time-course gene expression data with the CoGAPS matrix factorization. Methods Mol Biol *1101*, 87-112. 10.1007/978-1-62703-721-1_6.

Fertig, E.J.D., J., and Favorov, A.V.P., G. Ochs, M. F. (2010). CoGAPS: an R/C++ package to identify patterns and biological process activity in transcriptomic data. Bioinformatics *26*, 2792-2793. 10.1093/bioinformatics/btq503.

Kumar, S., Stecher, G., Suleski, M., and Hedges, S.B. (2017). TimeTree: A Resource for Timelines, Timetrees, and Divergence Times. Mol Biol Evol *34*, 1812-1819. 10.1093/molbev/msx116.

Kyttala, A., Moraghebi, R., Valensisi, C., Kettunen, J., Andrus, C., Pasumarthy, K.K., Nakanishi, M., Nishimura, K., Ohtaka, M., Weltner, J., et al. (2016). Genetic Variability Overrides the Impact

of Parental Cell Type and Determines iPSC Differentiation Potential. Stem Cell Reports *6*, 200-212. 10.1016/j.stemcr.2015.12.009.

Mallon, B.S., Chenoweth, J.G., Johnson, K.R., Hamilton, R.S., Tesar, P.J., Yavatkar, A.S., Tyson, L.J., Park, K., Chen, K.G., Fann, Y.C., and McKay, R.D. (2013). StemCellDB: the human pluripotent stem cell database at the National Institutes of Health. Stem cell research *10*, 57-66. 10.1016/j.scr.2012.09.002.

Muthusamy, T., Mukherjee, O., Menon, R., Megha, P.B., and Panicker, M.M. (2014). A method to identify and isolate pluripotent human stem cells and mouse epiblast stem cells using lipid body-associated retinyl ester fluorescence. Stem Cell Reports *3*, 169-184. 10.1016/j.stemcr.2014.05.004.

Neme, R., and Tautz, D. (2016). Fast turnover of genome transcription across evolutionary time exposes entire non-coding DNA to de novo gene emergence. eLife *5*, e09977. 10.7554/eLife.09977.

Pijuan-Sala, B., Griffiths, J.A., Guibentif, C., Hiscock, T.W., Jawaid, W., Calero-Nieto, F.J., Mulas, C., Ibarra-Soria, X., Tyser, R.C.V., Ho, D.L.L., et al. (2019). A single-cell molecular map of mouse gastrulation and early organogenesis. Nature *566*, 490-495. 10.1038/s41586-019-0933-9.

Stein-O'Brien, G.L., Carey, J.L., Lee, W.S., Considine, M., Favorov, A.V., Flam, E., Guo, T., Li, S., Marchionni, L., Sherman, T., et al. (2017). PatternMarkers & GWCoGAPS for novel data-driven biomarkers via whole transcriptome NMF. Bioinformatics, 1-3. 10.1093/bioinformatics/btx058.

Stein-O'Brien, G.L., Clark, B.S., Sherman, T., Zibetti, C., Hu, Q., Sealfon, R., Liu, S., Qian, J., Colantuoni, C., Blackshaw, S., et al. (2019). Decomposing Cell Identity for Transfer Learning across Cellular Measurements, Platforms, Tissues, and Species. Cell Syst *8*, 395-411 e398. 10.1016/j.cels.2019.04.004.

Vakirlis, N., Carvunis, A.R., and McLysaght, A. (2020). Synteny-based analyses indicate that sequence divergence is not the main source of orphan genes. eLife *9*. 10.7554/eLife.53500.

Weber, J.A., Park, S.G., Luria, V., Jeon, S., Kim, H.M., Jeon, Y., Bhak, Y., Jun, J.H., Kim, S.W., Hong, W.H., et al. (2020). The whale shark genome reveals how genomic and physiological properties scale with body size. Proceedings of the National Academy of Sciences of the United States of America *117*, 20662-20671. 10.1073/pnas.1922576117.

Zhai, J., Guo, J., Wan, H., Qi, L., Liu, L., Xiao, Z., Yan, L., Schmitz, D.A., Xu, Y., Yu, D., et al. (2022). Primate gastrulation and early organogenesis at single-cell resolution. Nature *612*, 732-738. 10.1038/s41586-022-05526-y.
